# Supplementary material for: Clinical assessment of pelvic floor and abdominal muscles 3 months post partum: an inter-rater reliability study
Source: BMJ Open. 2021 Sep 2;11(9):e049082. doi: 10.1136/bmjopen-2021-049082 (PMC8413957; doi:10.1136/bmjopen-2021-049082)
Supplement: Supplementary data [file bmjopen-2021-049082supp001.pdf]

Supplement 1: Clinical assessment of pelvic floor and abdominal muscles three months postpartum: An inter-rater reliability study

Clinical assessment protocol: Diastasis recti abdominis

Due to the lack of information about how to apply the jaws of the caliper while measuring the diastasis recti abdominis, we conducted a pilot trial with ten participants before starting the study. The inside and the outside jaws of the caliper were used in random order.

The outside jaws of the caliper are designed to measure the outer boundaries of a firm subject. In the setting of this study, this would be the outer boundaries of the linea alba. The inside jaws of the caliper are designed to measure the inner boundaries of a firm subject, which in the present study would be the inner edges of the rectus abdominis. We found a mean difference of −2.32 mm (SD 2.47) between the inside and the outside jaws. Moreover, measuring with the inside jaws caused more discomfort for the participant, and more insecurity for the investigator regarding the subjective estimation of how much to draw in the inner edge of the rectus abdominis. Thus, it was decided to palpate the outer edges of the linea alba with the fingers, and to measure this distance with the outside jaws (Figure 1).

Figure 1. *Application of the caliper for diastasis recti abdominis measurement*

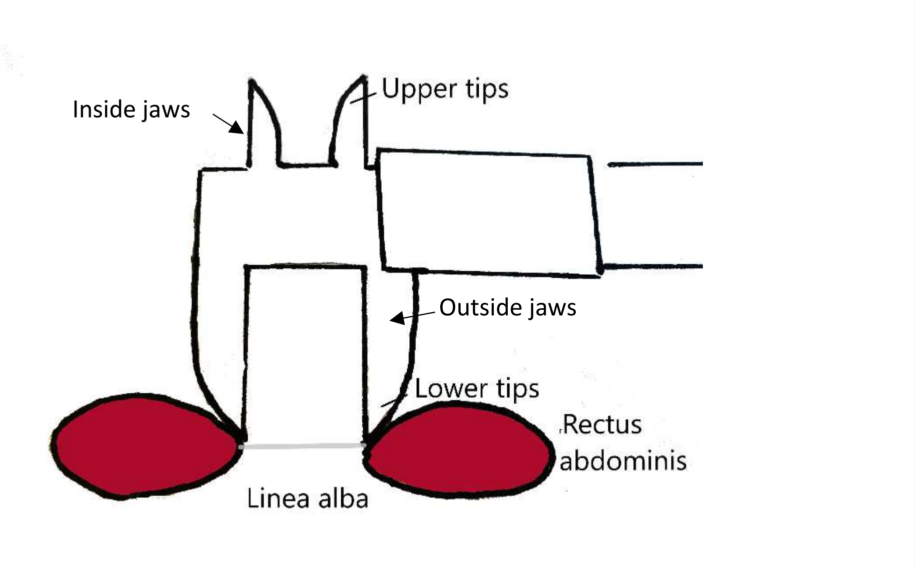

Supplement 1: Clinical assessment of pelvic floor and abdominal muscles three months postpartum: An inter-rater reliability study

Two months after start of the study in September 2018, the assessing physiotherapists expressed a strong uncertainty regarding the right technique for using the caliper. A pre-analysis of the data showed low-to-negative ICC values and large differences between the measurements. Thus, we conducted an additional training for all centers. The six physiotherapists underwent a calibration process in form of after each other assessing ten person`s abdominal muscles, comparing their results and discussing their technique. At this point in time, rehabilitation center 3 had not yet started their assessments. In the additional training, the assessing physiotherapists identified three important confounders while measuring:

1. Accurate lift of the head (2–3 cm) was an important factor for correct measurement. With less lift, there was no contraction in the abdominal muscles and it was almost impossible to find the inner edges of the *musculus rectus abdominis* and with a higher lift the distance between the two bellies of the *musculus recti abdominis* was decreasing which is in line with the study of Mota et al showing that an abdominal crunch is narrowing the inter-recti distance.[1]
2. Pre-activation in the deeper abdominal muscles impeded the measurement. Due to this the participants were encouraged to relax before lifting the head.
3. It was easier to palpate the junction between the rectus abdominis and linea alba during the eccentric movement of lowering the head.

<u>After the additional training, the assessment protocol was set as follows:</u>

The diastasis recti abdominis was assessed with the participant in the supine position on a flat bench, with the legs flexed, and their arms held at their side. The assessing physiotherapist used a water-soluble marker to mark the three measurement points: at the umbilicus, and at 4.5 cm above and 4.5 cm below the umbilicus.[2,3] For accurate assessment of the activated

abdominal muscles, the participant had to lift her head 2–3 cm from the bench. Before the assessment began, the physiotherapist assured that the participant correctly lifted her head 2–3 cm, which was trained by several repetitions.

Diastasis recti abdominis width: The diastasis recti abdominis width was defined as the measured distance between the two parts of the rectus abdominis.

- Clinical assessment: The participant was asked to lift her head and then slowly lower it. As seen in other studies[4,5], this concentric and eccentric movement is helping the physiotherapist to palpate the outer edges of the linea alba with their index and middle finger, without examination gloves. To avoid pre-activation of the deeper abdominal muscle, the participants were asked to relax before performing the trained head lift of 2–3 cm. During this movement -resulting in an activation of the musculus rectus abdominis- the physiotherapist identified the distance between the two parts of the rectus abdominis with her fingers and measured this felt distance using the caliper (Image 1a). The same procedure was conducted at all three measurement points.

***Image 1. a)** Measuring the diastasis recti abdominis width with the caliper. **b)** Observation of diastasis recti abdominis bulging*

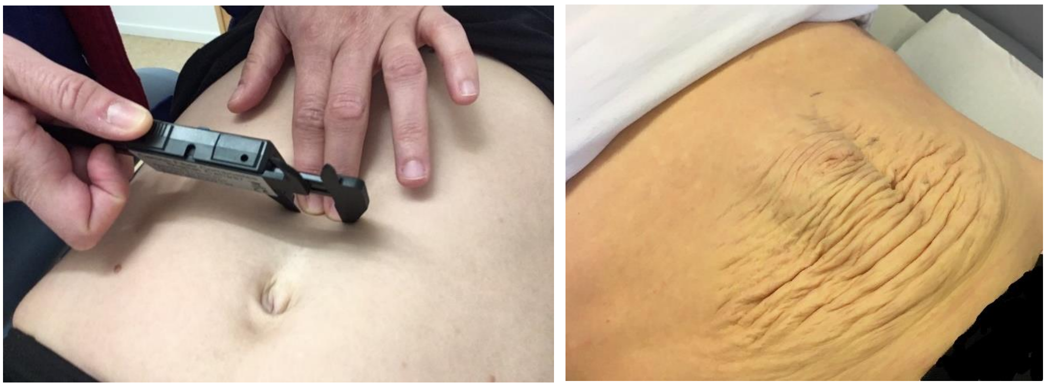

Supplement 1: Clinical assessment of pelvic floor and abdominal muscles three months postpartum: An inter-rater reliability study

Diastasis recti abdominis depth: The diastasis recti abdominis depth was defined as the palpated tension in the linea alba during a head lift.

- Clinical assessment: The participant was asked to repeat the exact same head lift of 2–3 cm. At all three measurement points, the physiotherapist palpated the tension in the linea alba (without adding pressure), and rated it as “good resistance at all points”, “resistance in the depth at measurement point x”, or “bottomless resistance at measurement point x”.

Diastasis recti abdominis bulging: Diastasis recti abdominis bulging was defined as midline bulge on exertion[6], which is also seen in Image 1b.

- Clinical assessment: The participant performed a 3-step sit-up test.[7] The participant was laying supine with straight legs on the flat bench. The test was rated as 0 if the participant was not able to perform a sit-up, as 1 if the participant was able to raise the upper torso to 40-degree angle from the bench with straight and secured legs, arms at the side of the body. The test was rated as 2 if the participant was able to perform the same task with the hands held behind the head and as 3 if the participant was able to perform a sit-up with the hands behind her head and hips and knees flexed and not secured. The participant had to hold the sit-up position for 5 seconds. During this test, the physiotherapist observed whether the linea alba bulged during the attempt to do a sit-up. Diastasis recti abdominis bulging was defined as midline bulge on exertion[6], which is also seen in Image 1b.

References:

1. Mota P, Pascoal AG, Carita AI, Bo K. The Immediate Effects on Inter-rectus Distance of Abdominal Crunch and Drawing-in Exercises During Pregnancy and the Postpartum Period. *J. Orthop. Sports Phys. Ther.* 2015;45(10):781-788.

2. Chiarello CM, Falzone LA, McCaslin KE, Patel MN, Ulery KR. The effects of an exercise program on diastasis recti abdominis in pregnant women. *Journal of Women's Health Physical Therapy.* 2005;29(1):11-16.
3. Chiarello CM, McAuley JA. Concurrent validity of calipers and ultrasound imaging to measure interrecti distance. *The Journal of orthopaedic and sports physical therapy.* 2013;43(7):495-503.
4. Parker MA, Millar LA, Dugan SA. Diastasis Rectus Abdominis and Lumbo-Pelvic Pain and Dysfunction-Are They Related? *Journal of Women's Health Physical Therapy.* 2009;33(2):15-22.
5. Dalal K, Kaur A, Mitra M. Correlation between Diastasis Rectus Abdominis and Lumbopelvic Pain and Dysfunction. *Indian Journal of Physiotherapy and Occupational Therapy.* 2014;8(1):210-214.
6. Lo T, Candido G, P J. Diastasis of the Recti abdominis in pregnancy: risk factors and treatment. *Physiother. Can.* 1999;51(1):32-44.
7. Hills NF, Graham RB, McLean L. Comparison of Trunk Muscle Function Between Women With and Without Diastasis Recti Abdominis at 1 Year Postpartum. *Phys. Ther.* 2018;98(10):891.
