# Supplementary material for: Clinical assessment of pelvic floor and abdominal muscles 3 months post partum: an inter-rater reliability study
Source: BMJ Open. 2021 Sep 2;11(9):e049082. doi: 10.1136/bmjopen-2021-049082 (PMC8413957; doi:10.1136/bmjopen-2021-049082)
Supplement: Supplementary data [file bmjopen-2021-049082supp002.pdf]

Supplement 2: Clinical assessment of pelvic floor and abdominal muscles three months postpartum: An inter-rater reliability study

Clinical assessment protocol: Pelvic floor muscles

The pelvic floor muscles were assessed with the participant in the supine position, with the legs flexed and slightly abducted on a plinth, and a pillow under the head. Participants were assessed by observation and digital palpation. During observation of pelvic floor muscle contraction, the physiotherapist stood beside the bench, supporting the participant's legs with her hands and observing the movement of the perineum:

- **Involuntary contraction**: The participant was asked to cough forcefully; the movement of the perineum were rated as downwards/ perineal in-drawing/ no movement
- **Voluntary contraction**, the participant was given the verbal cue "contract your pelvic floor muscles like you want to prevent the escape of gas/urine". The movement of the perineum was rated as downwards/ perineal in-drawing/ no movement

Vaginal palpation of the pelvic floor muscles was performed by physiotherapists using examinations gloves and water-based lubricant. The index and middle finger were inserted 2–3 cm into the vagina with the palmar side directed to the caudal part of the vagina, palpating the pubovisceral portion of the levator ani.[1]

- **Involuntary contraction**: The participant was asked to forcefully cough three times. The absence or presence of a correct contraction, defined as a squeeze around the pelvic openings and an inward lift,[2] were rated.
- **Maximal voluntary contraction,** the participant was asked to contract the pelvic floor muscles. If the physiotherapist felt a correct contraction, the participant was encouraged to activate their pelvic floor muscles "as strong and as long you can". Of three maximal voluntary contractions, the strongest was rated on a 6-point

Supplement 2: Clinical assessment of pelvic floor and abdominal muscles three months postpartum: An inter-rater reliability study

modified Oxford Scale. The scale was modified for the study with the aim to assess both the lifting and the squeezing component of the pelvic floor muscle function. The onset of the lifting component in the contraction were set as a criterion for being rated ≥3 on the scale (Figure 2).

***Figure 1**. The modified Oxford Scale used in this study*

0	No contraction
1	Partial contraction, “flicker”
2	Weak contraction, no correct lift
3	Good contraction, definite lift
4	Maximum contraction with lift, not able to hold longer than 5 seconds
5	Maximum contraction with lift, able to hold ___ seconds

The participants rested 15 seconds between the contractions. If a participant, despite several attempts and verbal cues, failed to squeeze and lift and was instead straining, the participant´s results were excluded from the statistical analysis of maximal voluntary contraction and pelvic floor muscle endurance.

- **Pelvic floor muscle endurance**, after 15 seconds of rest, the participant was asked to contract the pelvic floor muscles for as long as possible at approximately 50% of the previous contraction strength. Pelvic floor muscle endurance was rated as positive if the participant was able to hold this contraction for longer than 30 seconds.

Supplement 2: Clinical assessment of pelvic floor and abdominal muscles three months postpartum: An inter-rater reliability study

- **Voluntary relaxation**, the participant was given the verbal cue “try to relax your pelvic floor muscles, let the vagina get larger and go downwards”. This function was rated as absent, partial, or complete.

References:

1. Kearney R, Miller JM, DeLancey JOL. Interrater reliability and physical examination of the pubovisceral portion of the levator ani muscle, validity comparisons using MR imaging. Neurourol. Urodyn. 2006;25(1):50-54.
2. Bo K, Sherburn M. Evaluation of female pelvic-floor muscle function and strength. Phys. Ther. 2005;85(3):269-282.
