# Supplementary material for: Clinical assessment of pelvic floor and abdominal muscles 3 months post partum: an inter-rater reliability study
Source: BMJ Open. 2021 Sep 2;11(9):e049082. doi: 10.1136/bmjopen-2021-049082 (PMC8413957; doi:10.1136/bmjopen-2021-049082)
Supplement: Author's manuscript [file bmjopen-2021-049082.draft_revisions.pdf]

BMJ Open

BMJ Open is committed to open peer review. As part of this commitment we make the peer review history of every article we publish publicly available.

When an article is published we post the peer reviewers' comments and the authors' responses online. We also post the versions of the paper that were used during peer review. These are the versions that the peer review comments apply to.

The versions of the paper that follow are the versions that were submitted during the peer review process. They are not the versions of record or the final published versions. They should not be cited or distributed as the published version of this manuscript.

BMJ Open is an open access journal and the full, final, typeset and author-corrected version of record of the manuscript is available on our site with no access controls, subscription charges or pay-per-view fees (http://bmjopen.bmj.com).

If you have any questions on BMJ Open's open peer review process please email info.bmjopen@bmj.com

BMJ Open

Clinical assessment of pelvic floor and abdominal muscles three months postpartum: A reliability study

Journal:	*BMJ Open*
Manuscript ID	bmjopen-2021-049082
Article Type:	Original research
Date Submitted by the Author:	27-Jan-2021

Complete List of Authors:	Vesting, Sabine; Göteborgs universitet Institutionen för neurovetenskap och fysiologi, Department of Health and Rehabilitation<br>Olsen, Monika Fagevik; Göteborgs universitet Institutionen för neurovetenskap och fysiologi<br>Gutke, Annelie; Göteborgs universitet Institutionen för neurovetenskap och fysiologi<br>Rembeck, Gun; University of Gothenburg School of Public Health and Community Medicine<br>Larsson, Maria; Göteborgs universitet Institutionen för neurovetenskap och fysiologi

Keywords:	Maternal medicine < OBSTETRICS, Urinary incontinences < UROLOGY, REHABILITATION MEDICINE, Urogynaecology < GYNAECOLOGY

SCHOLARONE™ Manuscripts

I, the Submitting Author has the right to grant and does grant on behalf of all authors of the Work (as defined in the below author licence), an exclusive licence and/or a non-exclusive licence for contributions from authors who are: i) UK Crown employees; ii) where BMJ has agreed a CC-BY licence shall apply, and/or iii) in accordance with the terms applicable for US Federal Government officers or employees acting as part of their official duties; on a worldwide, perpetual, irrevocable, royalty-free basis to BMJ Publishing Group Ltd ("BMJ") its licensees and where the relevant Journal is co-owned by BMJ to the co-owners of the Journal, to publish the Work in this journal and any other BMJ products and to exploit all rights, as set out in our licence.

The Submitting Author accepts and understands that any supply made under these terms is made by BMJ to the Submitting Author unless you are acting as an employee on behalf of your employer or a postgraduate student of an affiliated institution which is paying any applicable article publishing charge ("APC") for Open Access articles. Where the Submitting Author wishes to make the Work available on an Open Access basis (and intends to pay the relevant APC), the terms of reuse of such Open Access shall be governed by a Creative Commons licence – details of these licences and which Creative Commons licence will apply to this Work are set out in our licence referred to above.

Other than as permitted in any relevant BMJ Author's Self Archiving Policies, I confirm this Work has not been accepted for publication elsewhere, is not being considered for publication elsewhere and does not duplicate material already published. I confirm all authors consent to publication of this Work and authorise the granting of this licence.

For peer review only

Clinical assessment of pelvic floor and abdominal muscles three months postpartum: A reliability study

Sabine Vesting, MSc [1, 2] 0000-0002-6523-1833

Annelie Gutke, PhD [1] 0000-0003-3484-9769

Monika Fagevik Olsén, PhD 1, [3+4] 0000-0002-0207-4105

Gun Rembeck, PhD [5-7] 0000-0003-0097-5150

Maria E.H. Larsson, PhD [1, 8] 0000-0002-1225-9736

Author information:

1 Department of Health and Rehabilitation, Unit of Physiotherapy, Institute of Neuroscience and Physiology, Sahlgrenska Academy, University of Gothenburg, Gothenburg, Sweden.

2 Närhälsan Gibraltar Rehabilitation, Gothenburg, Sweden.

3 Department of Physical Therapy and Occupational Therapy, Sahlgrenska University Hospital, Sweden

4 Department of Gastrosurgical Research & Education, Sahlgrenska Academy, Gothenburg University, Gothenburg, Sweden.

5 Region Västra Götaland, Research and Development Centre Södra Älvsborg, Borås, Sweden

6 Region Västra Götaland, Regionhälsan Borås Youth Centre, Borås, Sweden

7 Primary Health Care, School of Public Health and Community Medicine, Institute of Medicine, Sahlgrenska Academy, University of Gothenburg, Gothenburg, Sweden.

8 Region Västra Götaland Education, Research and Development Primary Health Care, Gothenburg, Sweden.

Corresponding author: Sabine Vesting, MSc., University of Gothenburg, Box 430, 405 30 Gothenburg, Sweden, mobile phone 0046-722095484, sabine.vesting@gu.se

Word count: Main document (3980), Abstract (300), Tables (5)

For peer review only - http://bmjopen.bmj.com/site/about/guidelines.xhtml

ABSTRACT

Objectives: Evaluation of the reliability of clinical assessment methods for pelvic floor muscles and diastasis recti abdominis after pregnancy.

Design: A multi-center reliability study.

Setting: Three primary care rehabilitation centers in West Sweden.

Participants: A total of 222 participants were recruited via advertising at Swedish maternity care units and social media. Eligibility for participation included female gender, ≥18 years, at maximum three months after childbirth, with the ability to understand and respond in Swedish. Exclusion criteria were chronic pelvic girdle pain and/or low back pain and/or pelvic floor tear grade III/IV. At each center, two physiotherapists assessed the 222 women according to a standardized protocol in random order.

Outcome measures: Reliability of the assessment of pelvic floor muscle function (involuntary and voluntary contraction and voluntary relaxation) and diastasis recti abdominis (width, depth and bulging).

Results: Vaginal palpation of maximal voluntary contraction revealed a kappa value of 0.69 [95% CI 0.62; 0.76]. Assessments of involuntary contraction, and voluntary relaxation yielded inconsistent results, with slight-to-moderate weighted kappa values ranging from 0.10–0.51. After two months of training in applying this method, diastasis recti abdominis width measured by caliper yielded an ICC value of 0.83 [95% CI 0.76; 0.87]. Assessments of diastasis recti abdominis depth and bulging showed moderate kappa values, with reservation for some inconsistency between the centers.

Conclusions: Vaginal palpation of pelvic floor muscle strength is a reliable method for the postpartum muscle assessment. Additional research is needed to identify reliable assessment method for other pelvic floor muscle functions like involuntary contraction and voluntary

For peer review only - http://bmjopen.bmj.com/site/about/guidelines.xhtml

For peer review only

relaxation. With some training, a caliper is a reliable instrument for measuring the postpartum diastasis recti abdominis width. This study is the first of its kind showing moderate inter-reliability of the assessment of diastasis recti abdominis bulging.

Trial registration: Registered at clinicaltrial.gov, October 2018, ClinicalTrials.gov Identifier NCT03703804.

Keywords: childbirth, caliper, diastasis recti abdominis, inter-rater, palpation

STRENGTHS AND LIMITATIONS

1. This study is adding new assessment methods to already known ones - testing a complete set of different muscle assessments methods postpartum for the use in primary care.
2. This study is adding two novel methods and scales for the assessment of diastasis recti abdominis depth and bulging.
3. The need of training and experience in the assessment of the diastasis recti abdominis were underestimated before start of the study.
4. Further validation of these methods by comparing the results with technical devices like ultrasound and pressure measurement, as well as testing them for their clinical relevance i.e. their association to pain and dysfunction, is a necessary next step.
5. The multi-center approach comparing results of different physiotherapists at three primary care rehabilitation centers allows a generalization of our results.

INTRODUCTION

The pelvic floor and abdominal muscles are parts of the human core, i.e., the anatomic and functional center of the human body.[1] Their function and activation play various roles in securing spine stability[2-4], posture[3], and continence.[5,6] In healthy women, these muscles work

For peer review only - http://bmjopen.bmj.com/site/about/guidelines.xhtml

together in voluntary or reflexive co-contractions.[7] During pregnancy and childbirth, these muscles are greatly stretched, resulting in postpartum muscular alterations,[8,9] which can lead to feelings of insecurity.

Women are increasingly seeking help and advice regarding postpartum core muscle changes from physiotherapists at primary health care centers. Childbirth is associated with pelvic floor traumas, such as perineal tears and levator ani injuries, which can lead to incontinence, pelvic organ prolapse, and decreased quality of life.[10,11] Another postpartum concern is a persistent separation of the two parts of the rectus abdominis, termed a diastasis recti abdominis. At 12 months postpartum, 33% of women exhibit a diastasis recti abdominis greater than the width of two fingers.[12] A diastasis recti abdominis is reportedly correlated with impaired quality of life, negative body image, and abdominal pain.[13,14]

Physiotherapists use various methods to assess the pelvic floor muscles and diastasis recti abdominis after pregnancy,[15,16] however, there is currently no gold standard. Clinically, vaginal palpation and observation are common methods to assess different pelvic floor muscle functions after childbirth. The international incontinence society defined that the pelvic floor muscle can be involuntarily and voluntarily contracted and can also be voluntarily relaxed.[17] However, there are no standardized assessment protocols or rating scales in the postpartum care, yet. One study reported the use of a Delphi scheme to identify the optimal protocol for assessing different pelvic floor muscle functions and tested their inter-rater reliability,[18] but these assessments were not performed in postpartum women.

Ultrasound assessment is the most reliable and valid method for measurement of the diastasis recti abdominis width.[19] However, most women who are concerned about their diastasis recti abdominis seek help at primary healthcare centers, where ultrasound is seldom available. About 96% of American physiotherapists specialized in women's health assess the diastasis recti abdominis width using the finger-width method,[12,20] which is imprecise due to

For peer review only - http://bmjopen.bmj.com/site/about/guidelines.xhtml

finger-width variations[21] and has weaker inter-rater reliability than instrumental assessment methods.[19] Less than 2% of American physiotherapists use calipers for the assessment of postpartum women.[20] Diastasis recti abdominis width assessment using a caliper is reported to be nearly as accurate as ultrasound assessment,[19,22] although the inter-rater reliability of this method has been tested in just one study.[23]

An experimental study shows that the tendon between the two parts of the rectus abdominis—the linea alba—exhibits a deviant behavior in a curl-up movement in women with diastasis recti abdominis, [24] these findings are strengthened by another similar study.[25] Both research groups argue that the diastasis recti abdominis depth and bulging also are relevant parameters in the diastasis recti abdominis assessment. These studies were performed using assessment by ultrasound [24] and shear-wave elastography.[25] However, there are currently no validated and clinically applicable assessment methods or rating scales for the parameters of diastasis recti abdominis depth and bulging.

In the present study, we aimed to evaluate the reliability of different aspects of the clinical assessment of pelvic floor muscle and diastasis recti abdominis using observation, calipers, and palpation at three months postpartum.

MATERIAL AND METHODS

Study design and subjects

In total, 222 women from the Region Västra Götaland, Sweden were included to this reliability study. Based on the guidelines of Ko & Li, we aimed to assess at least 30 participants at each center.[26] Assessments were conducted at three primary care rehabilitation centers. The study was registered at clinicaltrial.gov, October 2018, with the Identifier NCT03703804 and at the Research and Development Centre Gothenburg and Södra Bohuslän with the registration

For peer review only - http://bmjopen.bmj.com/site/about/guidelines.xhtml

number 243071. All included women gave their written informed consent to participate. The study protocol was approved by the Swedish ethical review authority in Gothenburg in April 2018 (Dnr 088-18) and registered with the local data protection service, Närhälsan Gothenburg, in January 2019.

The women were invited to participate via advertising at antenatal and childcare centers, and social media. Inclusion criteria were age of ≥18 years, vaginal delivery or caesarean section within the past three months, and the ability to understand and respond in Swedish. Exclusion criteria were chronic pelvic girdle pain and/or low back pain (defined as self-reported pelvic or low back pain for over three months, not related to the last pregnancy) and/or pelvic floor tear grade III/IV.

The participants were contacted and booked for assessment at one of the three primary care rehabilitation centers in the Västra Götaland region within three months after giving birth. Assessments were performed by six physiotherapists—two at each primary care rehabilitation center. Prior to the assessments, the participants completed a questionnaire about their age, body mass index (BMI), mode of delivery, number of delivered children, self-reported pelvic floor tears, most recent baby's birth weight, and the birth weights of previous children (if applicable).

The assessing physiotherapists had each completed a four-day (or longer) course in pelvic floor muscle assessment and treatment methods, and all had between one and nine years of experience in assessing pelvic floor muscles. All included physiotherapists were novices at measuring the diastasis recti abdominis by caliper, and on using the rating scales for depth and bulging. During the design phase of this study, four hours of training in diastasis recti abdominis measurement was planned.

For peer review only - http://bmjopen.bmj.com/site/about/guidelines.xhtml

Due to an insecurity among the physiotherapists in assessing and measuring the diastasis recti abdominis in the beginning of the study, the first two months of the study (63 measurements) were used for training and validation of the right technique for using the caliper and assessing depth and bulging. After these two months, the physiotherapists at all centers underwent additional training, comparing their results and discussing their technique. The whole validation process and the final assessment protocol after validation is available in Supplement 1.

Patient and public involvement

Patients and the public were not actively involved in the initial design of the study. However, the application of the caliper was tested in a pilot trial and the opinions of the participants about the application were considered for the design of the final assessment protocol (Supplement 1).

Clinical assessment of the pelvic floor muscles

The pelvic floor muscles were assessed with the participant in supine position, with the legs flexed and slightly abducted on a plinth, and a pillow under the head. The assessed pelvic floor muscle functions were involuntary and voluntary contraction (by observation and vaginal palpation) and maximal voluntary contraction, pelvic floor muscle endurance and voluntary relaxation (by vaginal palpation). The detailed assessment protocol is shown in Supplement 2.

Clinical assessment of the diastasis recti abdominis

The assessment of the diastasis recti abdominis was conducted in the same position as described above for the clinical assessment of the pelvic floor muscles. The physiotherapists assessed diastasis recti abdominis width using an electronic digital caliper (150 mm, carbon fiber, accuracy ±0.2 mm, 24 se Sverige AB, Kalmar, Sweden). Diastasis recti abdominis depth and bulging were assessed by observation and palpation. The step-wise explanation of caliper application and assessment of diastasis recti abdominis is explained in Supplement 1.

For peer review only - http://bmjopen.bmj.com/site/about/guidelines.xhtml

Upon completion of the assessment, the participants rested for 30 minutes in a sitting or lying position. After the 30-minute rest, the second physiotherapist conducted the same assessment as described above. The two assessing physiotherapists were blinded to each other's findings and were not allowed to talk about their assessments

Statistical analysis

Statistical analyses were performed using IBM SPSS statistical package version 25 (SPSS Inc., Chicago, IL). Descriptive statistics are presented as mean and standard deviation (SD)for ratio data, and as number and percentage for nominal and ordinal data. To calculate statistically significant differences between the three rehabilitation centers, we used the one-way ANOVA test for interval and ratio data, and the Kruskal-Wallis test for ordinal data. A p value of ≤0.05 was regarded as statistically significant.

All pelvic floor muscles functional measures were rated on ordinal scales, except for pelvic floor muscle endurance and involuntary contraction by palpation. Diastasis recti abdominis depth was also rated on an ordinal scale. Ratings on ordinal scales were evaluated by Cohen's weighted kappa values. Pelvic floor muscle endurance, involuntary contraction by palpation, and diastasis recti abdominis bulging were rated on nominal scales, and these ratings were evaluated by Cohen's kappa values. For interpretation of kappa values, we used the categories of Landis and Koch: <0.2, slight; 0.21–0.40, fair; 0.41–0.60, moderate; 0.61–0.80, substantial; and 0.81–1.0, almost perfect agreement.[27] Percentage agreement was calculated and presented for all nominal and ordinal data, and <60% agreement was defined as faulty agreement.[28]

For assessment of diastasis recti abdominis width, a continuous scale (in mm) was used. To evaluate the inter-rater reliability of the assessments on a continuous scale, we calculated the intraclass correlation coefficient (ICC) and 95% confidence interval (CI). ICC values were calculated in SPSS based on absolute agreement and a 2-way mixed effects model.

For peer review only - http://bmjopen.bmj.com/site/about/guidelines.xhtml

ICC values of <0.50 indicate poor reliability, 0.50–0.75 indicate moderate reliability, 0.75–0.90 good reliability, and values of >0.90 indicate excellent reliability [26].

To further evaluate reliability, we calculated the standard error of measurements ($SEM = SD \times \sqrt{(1 - ICC)}$), which represent the typical error in a single measurement and the minimal detectable change ($MDC = SEM \times 1.96 \times \sqrt{2}$). For calculation of the standard error of measurements (SEM), we used the standard deviation (SD) from the scores of all subjects. SEM and MDC values are presented in mm.

RESULTS

A total of 222 women were assessed, with measurements conducted from September 2018 through February 2020. The mean age of the participants was 33.1 years (SD ±3.3) and the majority of women (61%) had delivered one child (Table 1). The participating women at primary care rehabilitation center 3 were significantly younger than the women at primary care rehabilitation center 2 and had significantly more children and more vaginal deliveries than the participants at primary care rehabilitation centers 1 and 2.

For peer review only - http://bmjopen.bmj.com/site/about/guidelines.xhtml

Table 1. Characteristics of the participating women at three months postpartum (N=222)

	Total (n = 222)	Primary care rehabilitation center 1 (n = 90)	Primary care rehabilitation center 2 (n = 103)	Primary care rehabilitation center 3 (n = 29)
	Mean ±SD	*Mean* ±SD	*Mean* ±SD	*Mean* ±SD
**Age in years**	33.1 ±3.3	32.6 ±3.5	33.8 ±2.9	32.0 ±3.8
**BMI**	24.5 ±3.0	24.4 ±3.1	24.3 ±2.9	25.1 ±3.2
**Neonatal birth weight**	3574.5 ±507.0	3517.7 ±533.7	3604.7 ±489.2	3641.1 ±484.0
	n (%)	n (%)	n (%)	n (%)
**Delivery mode**				
C-section	28 (13)	9 (10)	16 (16)	3 (10)
Vaginal delivery	194 (87)	81 (90)	87(85)	26 (90)
**Number of children**				
1	137 (61)	51 (57)	75 (73)	11 (3)
2	74 (33)	33 (37)	26 (25)	15 (50)
3	9 (4)	7 (6)	1 (1)	1 (3)
4 or more	3 (1)	0 (0)	1 (1)	2 (7)
**Self-reported perineal tear**				
No tear	54 (24)	24 (27)	23 (22)	7 (24)
First-degree perineal tear	52 (23	16 (18)	24 (23)	12 (41)
Second-degree perineal tear /episiotomy	74 (33)	33 (38)	35 (34)	6 (20)

n, number; BMI, body mass index; SD, standard deviation.

For peer review only

Clinical assessment of the pelvic floor muscles

The evaluation of maximal voluntary contraction showed substantial agreement (weighted kappa value, 0.69 [95 % CI 0.62 to 0.76]), and assessment of pelvic floor muscle endurance showed moderate agreement (kappa value, 0.49 [95% CI 0.37 to 0.61]) (Table 2). Seven participants (3.3%) were excluded from the analyses of maximal voluntary contraction and

For peer review only - http://bmjopen.bmj.com/site/about/guidelines.xhtml

pelvic floor muscle endurance due to incorrect pelvic floor muscle contraction (straining). Assessment of voluntary contraction by observation showed moderate agreement, with a weighted kappa of 0.45 [95% CI 0.28 to 0.62]. Among all assessments, about 89% were rated as “perineal inward movement”.

Table 2. *Results of the clinical assessment of pelvic floor muscles via observation and vaginal palpation*

Parameters	Total group (n = 222)		Primary care rehabilitation center 1 (n = 90)		Primary care rehabilitation center 2 (n = 103)		Primary care rehabilitation center 3 (n = 29)	
	Kappa [95% CI]	PA %	Kappa [95% CI]	PA %	Kappa [95% CI]	PA %	Kappa [95% CI]	PA %
**Voluntary contraction**								
Observation	0.45 [0.28; 0.62]	90	0.40 [0.11; 0.70]	91	0.55 [0.33; 0.77]	90	0.29 [−0.13; 0.70]	86
Vaginal palpation								
MVC[a]	0.69 [0.62; 0.76]	71	0.70 [0.58; 0.82]	77	0.59 [0.46; 0.71]	67	0.67 [0.50; 0.84]	62
PFM endurance[a]	0.49 [0.37; 0.61]	74	0.68 [0.53; 0.83]	84	0.27 [0.09; 0.45]	65	0.55 [0.20; 0.90]	83
**Involuntary contraction**								
Observation	0.10 [−0.02; 0.22]	57	0.20 [−0.01; 0.42]	77	0.11 [−0.05; 0.27]	48	0.38 [0.10; 0.67]	68
Vaginal palpation	0.51 [0.37; 0.65]	85	0.26 [-0.04; 0.56]	87	0.47 [0.25; 0.69]	85	0.47 [0.14; 0.80]	75
**Voluntary relaxation**								
Vaginal palpation	0.26 [0.15; 0.37]	57	0.30 [0.16; 0.50]	63	−0.08 [−0.23; 0.07]	45	0.56 [0.07; 1.02]	89

n, number; CI, confidence interval; PA, percentage agreement; MVC, maximal voluntary contraction; PFM, pelvic floor muscle.

reduced number of participants due to incorrect pelvic floor muscle contraction (straining): total group (*n* = 215), primary care rehabilitation center 1 (*n* = 88), primary care rehabilitation center 2 (*n* = 98), primary care rehabilitation center 3 (*n* = 29).

For peer review only - http://bmjopen.bmj.com/site/about/guidelines.xhtml

The assessment of involuntary contraction by observation exhibited slight agreement (weighted kappa value, 0.10 [95% CI −0.02 to 0.22]). About 70% of participants were rated as “downward movement”, and 9–11% as upward movement. Fair-to-moderate kappa values were found for evaluation of involuntary contraction by palpation. Over 80% of participants were rated as “absence of correct contraction”.

Ratings of voluntary relaxation showed large variations between different primary care rehabilitation centers, with weighted kappa values ranging from −0.08 to 0.56. The application of the scale significantly differed between primary care rehabilitation center 3 and primary care rehabilitation centers 1 + 2. At primary care rehabilitation center 3, the physiotherapists rated 25 of 29 assessments as showing complete voluntary relaxation. In contrast, at primary care rehabilitation centers 1 + 2, 10–12% of participants were rated as absent voluntary relaxation, 66% as partly relaxed, and 20–24% as complete voluntary relaxation. Primary care rehabilitation center 2 showed a negative kappa value of −0.08, indicating an agreement worse than expected or no agreement.[28]

Clinical assessment of the diastasis recti abdominis

Diastasis recti abdominis width, depth, and bulging were assessed in 159 women. The mean diastasis recti abdominis widths at three months postpartum was 25.9 mm at the umbilicus (Table 3). The diastasis recti abdominis widths measured at rehabilitation center 3 were significantly wider compared to rehabilitation centers 1 and 2.

For peer review only - http://bmjopen.bmj.com/site/about/guidelines.xhtml

Table 3. *Diastasis recti abdominis width at three months postpartum (in mm) measured with a caliper*

	Total group (n = 159)	Primary care rehabilitation center 1 (n = 61)	Primary care rehabilitation center 2 (n = 69)	Primary care rehabilitation center 3 (n = 29)	*p* [b+c]
	Mean ±SD<br>95% CI	*Mean* ±SD<br>95% CI	*Mean* ±SD<br>95% CI	*Mean* ±SD<br>95% CI	
Above the umbilicus	22.0 ±7.4<br>[20.9; 23.2]	19.8 ±5.3<br>[18.5; 21.2]	20.6 ±4.6<br>[19.5; 21.7]	29.9 ±10.9<br>[25.8; 34.1]	<0.01
At the umbilicus	25.9 ±7.2<br>[24.8; 27.1]	24.1 ±6.5<br>[22.5; 25.8]	24.7 ±5.2<br>[23.5; 25.9]	32.8 ±9.0<br>[29.3; 36.2]	<0.01
Below the umbilicus	19.6 ±7.3<br>[18.4; 20.7]	14.8 ±5.8<br>[13.3; 16.2]	21.1 ±4.6<br>[20.0; 22.2]	26.1 ±9.0<br>[22.7; 29.6]	<0.01

n, number; SD, standard deviation.

b) *p* value of primary care rehabilitation center 1 vs Primary care rehabilitation center 2 vs Primary care rehabilitation center 1.

c) *p* value by one-way ANOVA test.

The measurement of diastasis recti abdominis width with a caliper showed good reliability when measured at the umbilicus and 4.5 cm below the umbilicus, and moderate reliability at 4.5 cm above the umbilicus (Table 4). For the total group, the SEM was between 4.05–4.75 mm, and the MDC was 11.23–13.17 mm. Sub-analysis of the different primary care rehabilitation centers revealed two negative outliers. At primary care rehabilitation center 2, assessment at 4.5 cm below the umbilicus showed an ICC value of 0.51 [95% CI: 0.20 to 0.70], which is at the lower boundary of the definition for moderate reliability. Assessment at 4.5 cm

For peer review only - http://bmjopen.bmj.com/site/about/guidelines.xhtml

above the umbilicus at primary care rehabilitation center 3 showed much lower ICC values compared to the other values. An ICC value of 0.40 indicates low reliability.

Table 4. *Results of the clinical assessment of diastasis recti abdominis width with a caliper (in mm)*

Parameters	Total group (*n* = 159)	Primary care rehabilitation center 1 (*n* = 61)	Primary care rehabilitation center 2 (*n* = 69)	Primary care rehabilitation center 3 (*n* = 29)
	ICC [95% CI] SEM; MDC	ICC [95% CI] SEM; MDC	ICC [95% CI] SEM; MDC	ICC [95% CI] SEM; MDC
Above the umbilicus	0.73 [0.63; 0.80] 4.75; 13.17	0.78 [0.63; 0.87] 5.32; 14.75	0.60 [0.36; 0.75] 3.46; 9.59	0.40 [−0.32; 0.72] 8.30; 23.01
At the umbilicus	0.83 [0.76; 0.87] 4.05; 11.23	0.85 [0.75; 0.91] 3.29; 9.12	0.62 [0.39; 0.77] 4.34; 12.03	0.82 [0.61; 0.91] 4.93; 13.66
Below the umbilicus	0.80 [0.72; 0.85] 4.40; 12.20	0.75 [0.58; 0.85] 3.64; 10.09	0.51 [0.20; 0.70] 4.03; 11.17	0.74 [0.43; 0.88] 6.16; 17.07

n, number; ICC, intraclass correlation coefficient; CI, confidence interval; SEM, standard error of measurements; MDC, minimal detectable change.

The assessment of diastasis recti abdominis depth showed fair-to-moderate weighted kappa values ranging from 0.34–0.43 (Table 5). In the assessment of the diastasis recti abdominis depth, 21% were assessed as "good resistance at all points", 67% as "resistance in the depth", and 12% as "bottomless". Assessment of diastasis recti abdominis bulging in the 3-step sit-up test showed moderate agreement (kappa value, 0.51 [95% CI 0.29 to 0.73]). The values primary care rehabilitation center 2 and 3 were lower than at primary care rehabilitation center and showed wide ranges in the 95% CI. Among the participants, about 81% were rated as "no bulging", 12% as "diastasis recti abdominis bulging", and 7% as "cannot assess".

For peer review only - http://bmjopen.bmj.com/site/about/guidelines.xhtml

Table 5. Results of the clinical assessment of diastasis recti abdominis depth and bulging via observation and palpation

	Total Group (*n* = 159)		Primary care rehabilitation center 1 (*n* = 61)		Primary care rehabilitation center 2 (*n* = 69)		Primary care rehabilitation center 3 (*n* = 29)	
	Kappa [95% CI]	PA %	Kappa [95% CI]	PA %	Kappa [95% CI]	PA %	Kappa [95% CI]	PA %
**Depth**	0.43 [0.29; 0.56]	69	0.37 [0.15; 0.59]	70	0.34 [0.11; 0.58]	71	0.36 [0.04; 0.69]	62
**Bulging**[d]	0.51 [0.29; 0.73]	88	0.77 [0.52; 1.02]	94	0.35 [0.04; 0.66]	83	0.36 [-0.16; 0.88]	88

n, number; CI, confidence interval; PA, percentage agreement.

b) The physiotherapists had the rating option of "cannot assess", and these assessments were excluded: Total group (*n*= 137); primary care rehabilitation center (*n* = 52); primary care rehabilitation center 2 (*n*= 59); and primary care rehabilitation center 3 (*n* = 26).

DISCUSSION

The main findings of this study are that physiotherapists managing women's health in primary care have reliable methods available to assess voluntary pelvic floor muscle contraction by vaginal palpation and diastasis recti abdominis width by caliper three months postpartum. On the other hand, the assessment of involuntary contraction by observation and voluntary relaxation had kappa values with slight-to-fair agreement. Data with such low agreement are not useful for clinical practice or research.[28] Further investigations are needed to improve the clinical applicability and reliable assessment of these functions.

Clinical assessment of the pelvic floor muscles

Our present results showed weighted kappa values of 0.59–0.70 for the assessment of maximal voluntary contraction, which are higher compared to previous studies.[29-31] One explanation could be differences between the rating scales used for this muscle function. For example, in two prior studies,[29,30] only the squeezing and not the lifting component of the contraction was rated. Devreese *at al* discussed the importance of the lifting component of pelvic floor muscle strength, showing that incontinent women showed less inward movement than continent

For peer review only - http://bmjopen.bmj.com/site/about/guidelines.xhtml

women.[32] Furthermore, the differences to our results could also be related to differences in the study population and smaller sample sizes in previous studies.

The assessment of pelvic floor muscle endurance showed moderate reliability. However, there were several inconsistencies in the pelvic floor muscle endurance data. Assessment of pelvic floor muscle endurance at primary care rehabilitation center 2 showed only fair reliability, with a kappa value of 0.27 [95% CI 0.09 to 0.45]. Our present results are lower compared with the findings of Devreese *et al.*.[32] Notably, in the study of Devreese *et al*, a contraction longer than 10 seconds was rated as positive rather than a contraction longer than 30 seconds, as in the present study. Additionally, their study population was older and not three months postpartum. It may be more difficult to assess the exact point of time when the contraction is subsiding in a longer time period. It is also possible that postpartum women have, on average, a weaker contraction, making it more difficult to assess pelvic floor muscle endurance. Further research is needed to decide whether a pelvic floor muscle endurance of 10 seconds or 30 seconds is more clinically relevant.

Voluntary contraction via observation showed fair-to-moderate weighted kappa values. A prior MRI study reported that the average inward movement of the perineum is about 1 cm while sitting, and it is more than 2 cm in the supine position, according to Kegel in 1952.[33] It could be assumed that this large movement would be easy to observe, and a higher kappa value was expected. A previous study reported high inter-rater reliability in the observation of inward perineum movement.[18] Correspondingly, another study showed that inward perineal movement could be observed with a kappa value of 0.91 among continent women, and 0.93 among incontinent women- [32]

As factors other than pelvic floor muscle strength may contribute to urinary leakage postpartum,[34] it is important to assess other aspects of pelvic floor muscle function. Unfortunately, in our present study, we did not find a reliable method for assessing involuntary

For peer review only - http://bmjopen.bmj.com/site/about/guidelines.xhtml

contraction by observation, and we demonstrated inconsistent findings for the assessment of involuntary contraction by palpation. Accordingly, another study reported only fair inter-rater reliability for the assessment of involuntary contraction by observation and palpation.[18] Further studies are needed to develop improved methods for the assessment and rating of these pelvic floor muscle functions in clinical practice.

When prescribing postpartum pelvic floor muscle training, it must be considered that some women have overactive, and possibly painful pelvic floor muscles. [35] It remains unclear whether women with hypertonic pelvic floor muscles should be advised to do pelvic floor muscle training. In clinical practice, physiotherapists recommend an individualized approach. Our present results showed that the rating of voluntary relaxation had slight-to-fair inter-rater reliability. Slieker-ten *et al.* [18] reported similar findings. Another study used a five-step rating scale for relaxation after contraction, and reported a correlation of 0.34 between two raters.[35] Even, Slieker-ten *et al.* recommend the addition of more rating steps to the scale, e.g., incomplete relaxation in their discussion. Regardless of whether women with hypertonic pelvic floor muscles need more support in pelvic floor muscle training or the recommendation of no pelvic floor muscle training, there remains a need for better methods of assessing this condition.

Clinical assessment of the diastasis recti abdominis

Our results from the present study showed moderate-to-good reliability in measuring diastasis recti abdominis width using a caliper, after two months of training and calibrating of the method. The characteristics of the diastasis recti abdominis at three months postpartum measured with the caliper (Table 3) were comparable with the diastasis recti abdominis characteristics after pregnancy measured by ultrasound,[36] indicating the measured values in our present study are true values for this population. However, the two months of training and calibrating of the method via discussion and additional training were important components of this study. One bias identified in our additional training was that the accurate head lift of just

For peer review only - http://bmjopen.bmj.com/site/about/guidelines.xhtml

2–3 cm was an important factor. This observation is supported by the study of Mota *et al.* [37] which showed that the distance between the two parts of the rectus abdominis decreased during a sit-up movement.

There was some inconsistencies in the data, with two outliers at primary care rehabilitation centers 2 + 3. The SEM and MDC were higher at primary care rehabilitation center 3 compared to primary care rehabilitation centers 1 + 2 (Table 3). The MDC measured at 4.5 cm above the umbilicus was over 2 cm, raising doubt about whether these results have any clinical relevance. At primary care rehabilitation center 3, fewer than 30 participants were recruited during the study period of over one year. These results indicate that in addition to training and experience, some continuity in measuring the diastasis recti abdominis width with a caliper is necessary for reliable assessment. It is also possible that it is more difficult to measure a wider diastasis recti abdominis, considering that the participants at primary care rehabilitation center 3 had significantly larger diastasis recti abdominis widths.

The SEM in our present study was about 4–5 mm and the MDC was about 11-13 mm, which is higher than in the comparable study of Benjamin *et al.*[23] However, just two participants showed an inter-recti distance greater than 22 mm in their study, in our study the mean diastasis recti abdominis width was 25.9 mm. Furthermore, Benjamin *et al* is not discussing how much experience and training the two investigating physiotherapists had in measuring diastasis recti abdominis width with a caliper.

We found fair-to-moderate weighted kappa values for the assessment of diastasis recti abdominis depth. To the best of our knowledge, there is no other study with which to compare these results. About 63–66% of the assessments were rated as "resistance in the depth". The investigating physiotherapists hypothesized that "resistance in the depth" was felt and assessed as soon as the participants did not activate their deeper abdominal muscles during the head lift. Accordingly, Lee and Hodges[24] described the increased tension in the linea alba

For peer review only - http://bmjopen.bmj.com/site/about/guidelines.xhtml

caused by activation of the deep abdominal muscles. Future studies must more precisely define the pre-activation of the deep abdominal muscles in the assessment of diastasis recti abdominis depth. Similar considerations are raised about abdominal muscle pre-activation and diastasis recti abdominis bulging during a sit-up curl.

Strengths and limitations

One strength of this study was the large sample size and the quantity of different aspects of muscle function. A comparable study assessing different aspects of pelvic floor muscle function in women with and without pelvic floor disorders included only 41 participants.[18] A review about diastasis recti abdominis assessment methods included studies with 20–106 participants, and these studies only examined diastasis recti abdominis width.[19] Another strength of this study was that we were able to perform the same tests at three different centers in different parts of west Sweden. This makes our results transferable for different physiotherapists using the same methods.

The present study also had several limitations. One was that we lacked access to the participants' delivery records. Thus, our analysis of statistically significant differences associated with age, BMI, mode of delivery, pelvic floor tearing, and highest birth weight were based on self-reported data from the participants. Another issue was the uneven cell distribution seen in over 50% of the rating scales tested in this study. In the literature, it is controversial whether a low kappa value can be explained by uneven cell distribution or low prevalence of a condition. [38]

Another limitation of this study was the initial assumption that four hours of training in measuring the diastasis recti abdominis width with a caliper would be enough training for the six assessing physiotherapists. Due to this misjudgment of complexity using this method, a total of 63 measurements had to be excluded. On the other hand, many different obstacles and sources of error for the measurements could be found through this initial phase

For peer review only - http://bmjopen.bmj.com/site/about/guidelines.xhtml

and the added training. It would have been even better to validate the caliper measurements against ultrasound measurements. However, the aim of this study was to evaluate clinical applicable methods, which are easy to use and inexpensive tools for daily practice.

Future research

For a next step, we must investigate the extent to which these values are clinically relevant for postpartum women—for example, if the assessment outcomes are associated with pain and dysfunction. Furthermore, we must determine a cut-off point for diastasis recti abdominis severity relative to pain and dysfunction. It will also be important to determine what training advice should be given to postpartum women based on the results of these examinations.

There also remains a need for further research on how to assess and rate involuntary contraction by observation and palpation, and voluntary relaxation, in the clinical assessment of women after pregnancy. Furthermore, additional research is needed regarding the assessment of the diastasis recti abdominis, in terms of depth and bulging of the linea alba in movement.

CONCLUSION

Women are increasingly demanding assessment of their pelvic floor muscles and diastasis recti abdominis after pregnancy. Our present results revealed moderate-to-substantial reliability for the assessment of maximal voluntary contraction and pelvic floor muscle endurance by vaginal palpation, considering both the lifting and squeezing component of the contraction. Furthermore, diastasis recti abdominis width can be measured by caliper, with a standard error of measurements of 4–5 mm and a minimal detectable change of about 1.2 cm. However, assessment using this instrument requires some training and experience.

For peer review only - http://bmjopen.bmj.com/site/about/guidelines.xhtml

Acknowledgements

We thank Lisa Altvall, Ulrika Hansson, Anna-Lena Magnberg, Vanesa Rufete Bernal, Ute Jesberg, and Johanna Ekberg, who assessed the 222 women in the current study.

Competing Interests

The authors report no conflict of interest.

Funding statement

This study was funded by the Local Research and Development Council Gothenburg and Södra Bohuslän (Grant Number VGFOUGSB-762581) and The Healthcare Board, Region Västra Götaland (Grant Number VGFOUREG-751101).

Availability of data and materials

The dataset from this study is available via a reasonable request to the corresponding author.

Author statement

Each author of this paper meets the criteria for authorship. SV, MFO, AG, GR, and MEHL designed the study. SV collected the data, analyzed and performed a first interpretation of the results, and drafted the article. MFO, AG, GR, and MEHL participated in the final interpretation of the results, and critically revised the article for important intellectual content. The final manuscript was seen and approved by all authors.

Consent for publication

The individuals shown in Image a+b gave their written informed consent to have these images published in a scientific publication.

For peer review only

List of Abbreviations

CI: Confidence interval; BMI: Body mass index; ICC: Intraclass correlation coefficient; MDC: Minimal detectable change; PA: Percentage agreement; SD: Standard deviation; SEM: Standard error of measurements

REFERENCES

1. Bliss LS, Teeple P. Core Stability: The Centerpiece of Any Training Program. *Curr. Sports Med. Rep.* 2005;4(3):179-183.
2. Sahrmann S. Diagnosis and treatment of movement impairment syndromes. St. Louis, Mo.: Mosby; 2002.
3. Hodges PW, Sapsford R, Pengel LHM. Postural and respiratory functions of the pelvic floor muscles. *Neurourol. Urodyn.* 2007;26(3):362-371.
4. Hodges PW, Eriksson AE, Shirley D, Gandevia SC. Intra-abdominal pressure increases stiffness of the lumbar spine. *J. Biomech.* 2005;38(9):1873-1880.
5. DeLancey JO. Structural support of the urethra as it relates to stress urinary incontinence: the hammock hypothesis. *Am. J. Obstet. Gynecol.* 1994;170(6):1713-1720; discussion 1720-1713.
6. Moser H, Leitner M, Baeyens JP, Radlinger L. Pelvic floor muscle activity during impact activities in continent and incontinent women: a systematic review. *Int Urogynecol J.* 2018;29(2):179-196.
7. Sapsford RR, Hodges PW, Richardson CA, Cooper DH, Markwell SJ, Jull GA. Co-activation of the abdominal and pelvic floor muscles during voluntary exercises. *Neurourol. Urodyn.* 2001;20(1):31.

For peer review only

8. Fernandes da Mota PG, Pascoal AG, Carita AI, Bo K. Prevalence and risk factors of diastasis recti abdominis from late pregnancy to 6 months postpartum, and relationship with lumbo-pelvic pain. *Man. Ther.* 2015;20(1):200-205.

9. Ashton-Miller JA, DeLancey JOL. On the Biomechanics of Vaginal Birth and Common Sequelae. *Annual Review of Biomedical Engineering.* 2009;11(1):163-176.

10. Cyr M-P, Kruger J, Wong V, Dumoulin C, Girard I, Morin M. Pelvic floor morphometry and function in women with and without puborectalis avulsion in the early postpartum period. *Am. J. Obstet. Gynecol.* 2017;216(3):274.e271-274.e278.

11. Memon H, Handa VL. Pelvic floor disorders following vaginal or cesarean delivery. *Curr. Opin. Obstet. Gynecol.* 2012;24(5):349-354.

12. Sperstad JB, Tennfjord MK, Hilde G, Ellström-Engh M, Bø K. Diastasis recti abdominis during pregnancy and 12 months after childbirth: prevalence, risk factors and report of lumbopelvic pain. *British Journal of Sports Medicine.* 2016.

13. Keshwani N, Mathur S, McLean L. Relationship Between Interrectus Distance and Symptom Severity in Women With Diastasis Recti Abdominis in the Early Postpartum Period. *Phys. Ther.* 2018;98(3):182-190.

14. Vieillefosse S, Conté C, Deffieux X. Symptom associated with diastasis recti in the early postpartum period. *European Journal of Obstetrics & Gynecology and Reproductive Biology.* 2019;234:e133-e133.

15. Keeler J, Albrecht M, Eberhardt L, Horn L, Donnelly C, Lowe D. Diastasis Recti Abdominis: A Survey of Women's Health Specialists for Current Physical Therapy Clinical Practice for Postpartum Women. *Journal of Women's Health Physical Therapy.* 2012;36(3):131-142.

16. Bo K, Sherburn M. Evaluation of female pelvic-floor muscle function and strength. *Phys. Ther.* 2005;85(3):269-282.

For peer review only - http://bmjopen.bmj.com/site/about/guidelines.xhtml

17. Messelink B, Benson T, Berghmans B, et al. Standardization of terminology of pelvic floor muscle function and dysfunction: report from the pelvic floor clinical assessment group of the International Continence Society. *Neurourol. Urodyn.* 2005;24(4):374-380.

18. Slieker-Ten Hove MCP, Pool-Goudzwaard AL, Eijkemans MJC, Steegers-Theunissen RPM, Burger CW, Vierhout ME. Face validity and reliability of the first digital assessment scheme of pelvic floor muscle function conform the new standardized terminology of the International Continence Society. *Neurourol. Urodyn.* 2009;28(4):295-300.

19. van de Water ATM, Benjamin DR. Measurement methods to assess diastasis of the rectus abdominis muscle (DRAM): A systematic review of their measurement properties and meta-analytic reliability generalisation. *Man. Ther.* 2016;21:41-53.

20. Keeler J, Albrecht M, Eberhardt L, Horn L, Donnelly C, Lowe D. Diastasis Recti Abdominis: A Survey of Women's Health Specialists for Current Physical Therapy Clinical Practice for Postpartum Women. *Journal of Women's Health Physical Therapy.* 2012;36(3):131-142.

21. Bursch SG. Interrater reliability of diastasis recti abdominis measurement. *Phys Ther.* 1987;67(7):1077-1079.

22. Barbosa S, de Sa RA, Coca Velarde LG. Diastasis of rectus abdominis in the immediate puerperium: correlation between imaging diagnosis and clinical examination. *Archives of gynecology and obstetrics.* 2013;288(2):299-303.

23. Benjamin DR, Frawley HC, Shields N, Georgiou C, Taylor NF. Establishing measurement properties in the assessment of inter-recti distance of the abdominal muscles in a postnatal women. *Musculoskeletal science & practice.* 2020;49:102202.

For peer review only

For peer review only - http://bmjopen.bmj.com/site/about/guidelines.xhtml

24. Lee D, Hodges PW. Behavior of the Linea Alba During a Curl-up Task in Diastasis Rectus Abdominis: An Observational Study. *The Journal of orthopaedic and sports physical therapy.* 2016;46(7):580.

25. Beamish N, Green N, Nieuwold E, McLean L. Differences in Linea Alba Stiffness and Linea Alba Distortion Between Women With and Without Diastasis Recti Abdominis: The Impact of Measurement Site and Task. *The Journal of orthopaedic and sports physical therapy.* 2019;49(9):656-665.

26. Koo TK, Li MY. A Guideline of Selecting and Reporting Intraclass Correlation Coefficients for Reliability Research. *J. Chiropr. Med.* 2016;15(2):155-163.

27. Landis JR, Koch GG. A One-Way Components of Variance Model for Categorical Data. *Biometrics.* 1977;33(4):671-679.

28. McHugh ML. Interrater reliability: the kappa statistic. *Biochemia medica.* 2012;22(3):276-282.

29. Bø K, Finckenhagen HB. Vaginal palpation of pelvic floor muscle strength: inter-test reproducibility and comparison between palpation and vaginal squeeze pressure. *Acta Obstet. Gynecol. Scand.* 2001;80(10):883-887.

30. Ferreira CH, Barbosa PB, de Oliveira Souza F, Antonio FI, Franco MM, Bo K. Inter-rater reliability study of the modified Oxford Grading Scale and the Peritron manometer. *Physiotherapy.* 2011;97(2):132-138.

31. Navarro Brazalez B, Torres Lacomba M, de la Villa P, et al. The evaluation of pelvic floor muscle strength in women with pelvic floor dysfunction: A reliability and correlation study. *Neurourol. Urodyn.* 2018;37(1):269-277.

32. Devreese A, Staes F, De Weerdt W, et al. Clinical evaluation of pelvic floor muscle function in continent and incontinent women. *Neurourol. Urodyn.* 2004;23(3):190-197.

For peer review only - http://bmjopen.bmj.com/site/about/guidelines.xhtml

33. Bo K, Lilleas F, Talseth T, Hedland H. Dynamic MRI of the pelvic floor muscles in an upright sitting position. *Neurourol. Urodyn.* 2001;20(2):167-174.

34. Fritel X, Ringa V, Quiboeuf E, Fauconnier A. Female urinary incontinence, from pregnancy to menopause: a review of epidemiological and pathophysiological findings. *Acta Obstet. Gynecol. Scand.* 2012;91(8):901-910.

35. Reissing ED, Brown C, Lord MJ, Binik YM, Khalifé S. Pelvic floor muscle functioning in women with vulvar vestibulitis syndrome. *Journal of Psychosomatic Obstetrics & Gynecology.* 2005;26(2):107-113.

36. Mota P, Pascoal AG, Carita AI, Bo K. Normal width of the inter-recti distance in pregnant and postpartum primiparous women. *Musculoskeletal science & practice.* 2018;35:34-37.

37. Mota P, Pascoal AG, Carita AI, Bo K. The Immediate Effects on Inter-rectus Distance of Abdominal Crunch and Drawing-in Exercises During Pregnancy and the Postpartum Period. *J. Orthop. Sports Phys. Ther.* 2015;45(10):781-788.

38. Vach W. The dependence of Cohen's kappa on the prevalence does not matter. *J. Clin. Epidemiol.* 2005;58(7):655-661.

For peer review only

For peer review only - http://bmjopen.bmj.com/site/about/guidelines.xhtml

Supplement 1: Clinical assessment of pelvic floor and abdominal muscles three months postpartum: A reliability study

Clinical assessment protocol: Diastasis recti abdominis

Due to the lack of information about how to apply the jaws of the caliper while measuring the diastasis recti abdominis, we conducted a pilot trial with ten participants before starting the study. The inside and the outside jaws of the caliper were used in random order.

The outside jaws of the caliper are designed to measure the outer boundaries of a firm subject. In the setting of this study, this would be the outer boundaries of the linea alba. The inside jaws of the caliper are designed to measure the inner boundaries of a firm subject, which in the present study would be the inner edges of the rectus abdominis. We found a mean difference of −2.32 mm (SD 2.47) between the inside and the outside jaws. Moreover, measuring with the inside jaws caused more discomfort for the participant, and more insecurity for the investigator regarding the subjective estimation of how much to draw in the inner edge of the rectus abdominis. Thus, it was decided to palpate the outer edges of the linea alba with the fingers, and to measure this distance with the outside jaws (Figure 1).

For peer review only

Supplement 1: Clinical assessment of pelvic floor and abdominal muscles three months postpartum: A reliability study

Figure 1. *Application of the caliper for diastasis recti abdominis measurement*

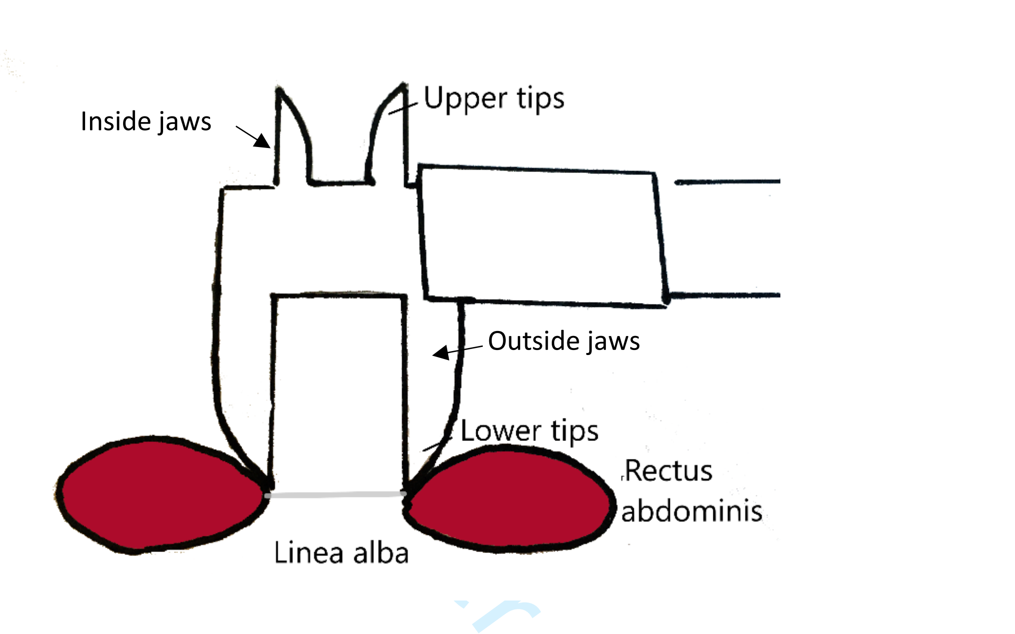

Two months after start of the study in September 2018, the assessing physiotherapists expressed a strong uncertainty regarding the right technique for using the caliper. A pre-analysis of the data showed low-to-negative ICC values and large differences between the measurements. Thus, we conducted an additional training for all centers. The six physiotherapists underwent a calibration process in form of after each other assessing ten person`s abdominal muscles, comparing their results and discussing their technique. At this point in time, rehabilitation center 3 had not yet started their assessments. In the additional training, the assessing physiotherapists identified three important confounders while measuring:

1. Accurate lift of the head (2–3 cm) was an important factor for correct measurement.
2. Pre-activation in the abdominal muscles impeded the measurement.

Supplement 1: Clinical assessment of pelvic floor and abdominal muscles three months postpartum: A reliability study

3. It was easier to palpate the junction between the rectus abdominis and linea alba during the eccentric movement of lowering the head.

After the additional training, the assessment protocol was set as follows:

The diastasis recti abdominis was assessed with the participant in the supine position, with the legs flexed, on a plinth. The assessing physiotherapist used a water-soluble marker to mark the three measurement points: at the umbilicus, and at 4.5 cm above and 4.5 cm below the umbilicus.[1,2] For accurate assessment, the participant had to lift her head 2–3 cm from the plinth, with no pillow. Before the assessment began, the physiotherapist assured that the participant correctly lifted her head 2–3 cm, which was trained by several repetitions.

- **Diastasis recti abdominis width:** The participant was asked to lift her head and then slowly lower it. During this concentric and eccentric movement, the physiotherapist palpated the outer edges of the linea alba with their index and middle finger, without examination gloves. Next, the participant was instructed to relax her muscles and perform the trained head lift of 2–3 cm. During this movement, the physiotherapist identified the distance between the two parts of the rectus abdominis with her fingers and measured this felt distance using the caliper (Image 1a). The same procedure was conducted at all three measurement points.

For peer review only

Supplement 1: Clinical assessment of pelvic floor and abdominal muscles three months postpartum: A reliability study

Image 1. a) *Measuring the diastasis recti abdominis width with the caliper.* ***b)*** *Observation of diastasis recti abdominis bulging*

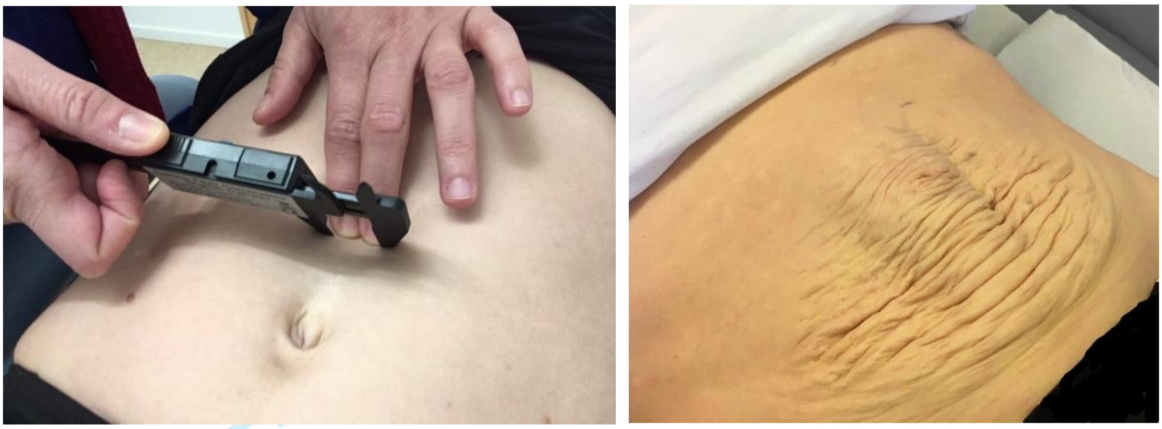

- **Diastasis recti abdominis depth:** The participant was asked to repeat the exact same head lift of 2–3 cm. At all three measurement points, the physiotherapist palpated the resistance in the linea alba, and rated it as "good resistance at all points", "resistance in the depth at measurement point x", or "bottomless resistance at measurement point x".
- **Diastasis recti abdominis bulging:** The participant performed a 3-step sit-up test, as previously described.[3] During this test, the physiotherapist observed whether the linea alba bulged during the movement (Image 1b).

References:

1. Chiarello CM, Falzone LA, McCaslin KE, Patel MN, Ulery KR. The effects of an exercise program on diastasis recti abdominis in pregnant women. Journal of Women's Health Physical Therapy. 2005;29(1):11-16.
2. Chiarello CM, McAuley JA. Concurrent validity of calipers and ultrasound imaging to measure interrecti distance. The Journal of orthopaedic and sports physical therapy. 2013;43(7):495-503.

For peer review only

Supplement 1: Clinical assessment of pelvic floor and abdominal muscles three months postpartum: A reliability study

3. Hills NF, Graham RB, McLean L. Comparison of Trunk Muscle Function Between Women With and Without Diastasis Recti Abdominis at 1 Year Postpartum. Phys. Ther. 2018;98(10):891.

For peer review only

Supplement 2: Clinical assessment of pelvic floor and abdominal muscles three months postpartum: A reliability study

Clinical assessment protocol: Pelvic floor muscles

The pelvic floor muscles were assessed with the participant in the supine position, with the legs flexed and slightly abducted on a plinth, and a pillow under the head. Participants were assessed by observation and digital palpation. During observation of pelvic floor muscle contraction, the physiotherapist stood beside the plinth, holding the participant's legs and observing the movement of the perineum:

- **Involuntary contraction**: The participant was asked to cough forcefully; the movement of the perineum were rated as downwards/ perineal in-drawing/ no movement
- **Voluntary contraction**, the participant was given the verbal cue "contract your pelvic floor muscles like you want to prevent the escape of gas/urine". The movement of the perineum was rated as downwards/ perineal in-drawing/ no movement

Vaginal palpation of the pelvic floor muscles was performed by physiotherapists using examinations gloves and water-based lubricant. The index and middle finger were inserted 2–3 cm into the vagina with the palmar side directed to the caudal part of the vagina, palpating the pubovisceral portion of the levator ani.[1]

- **Involuntary contraction**: The participant was asked to forcefully cough three times. The absence or presence of a correct contraction, defined as a squeeze around the pelvic openings and an inward lift,[2] were rated.
- **Maximal voluntary contraction,** the participant was asked to contract the pelvic floor muscles. If the physiotherapist felt a correct contraction, the participant was

For peer review only

Supplement 2: Clinical assessment of pelvic floor and abdominal muscles three months postpartum: A reliability study

encouraged to activate their pelvic floor muscles "as strong and as long you can". Of three maximal voluntary contractions, the strongest was rated on a 6-point modified Oxford Scale. The scale was modified for the study with the aim to assess both the lifting and the squeezing component of the pelvic floor muscle function. The onset of the lifting component in the contraction were set as a criterion for being rated ≥3 on the scale (Figure 2).

***Figure 1**. The modified Oxford Scale used in this study*

**0**	No contraction
**1**	Partial contraction, "flicker"
**2**	Weak contraction, no correct lift
**3**	Good contraction, definite lift
**4**	Maximum contraction with lift, not able to hold longer than 5 seconds
**5**	Maximum contraction with lift, able to hold ___ seconds

The participants rested 15 seconds between the contractions. If a participant, despite several attempts and verbal cues, failed to squeeze and lift and was instead straining, the participant´s results were excluded from the statistical analysis of maximal voluntary contraction and pelvic floor muscle endurance.

- **Pelvic floor muscle endurance**, after 15 seconds of rest, the participant was asked to contract the pelvic floor muscles for as long as possible at approximately 50% of the previous contraction strength. Pelvic floor muscle endurance was rated as

For peer review only

positive if the participant was able to hold this contraction for longer than 30 seconds.

- **Voluntary relaxation**, the participant was given the verbal cue “try to relax your pelvic floor muscles, let the vagina get larger and go downwards”. This function was rated as absent, partial, or complete.

For peer review only

References:

1. Kearney R, Miller JM, DeLancey JOL. Interrater reliability and physical examination of the pubovisceral portion of the levator ani muscle, validity comparisons using MR imaging. Neurourol. Urodyn. 2006;25(1):50-54.
2. Bo K, Sherburn M. Evaluation of female pelvic-floor muscle function and strength. Phys. Ther. 2005;85(3):269-282.

Section & Topic	No	Item	Reported on page #
**TITLE OR ABSTRACT**			
	**1**	Identification as a study of diagnostic accuracy using at least one measure of accuracy (such as sensitivity, specificity, predictive values, or AUC)	1
**ABSTRACT**			2
	**2**	Structured summary of study design, methods, results, and conclusions (for specific guidance, see STARD for Abstracts)	
**INTRODUCTION**			
	**3**	Scientific and clinical background, including the intended use and clinical role of the index test	3-5
	**4**	Study objectives and hypotheses	5
**METHODS**			
Study design	**5**	Whether data collection was planned before the index test and reference standard were performed (prospective study) or after (retrospective study)	5
Participants	**6**	Eligibility criteria	6
	**7**	On what basis potentially eligible participants were identified (such as symptoms, results from previous tests, inclusion in registry)	6
	**8**	Where and when potentially eligible participants were identified (setting, location and dates)	6
	**9**	Whether participants formed a consecutive, random or convenience series	6
Test methods	**10a**	Index test, in sufficient detail to allow replication	7, supplement 1+2
	**10b**	Reference standard, in sufficient detail to allow replication	N/A
	**11**	Rationale for choosing the reference standard (if alternatives exist)	N/A
	**12a**	Definition of and rationale for test positivity cut-offs or result categories of the index test, distinguishing pre-specified from exploratory	N/A
	**12b**	Definition of and rationale for test positivity cut-offs or result categories of the reference standard, distinguishing pre-specified from exploratory	N/A
	**13a**	Whether clinical information and reference standard results were available to the performers/readers of the index test	17
	**13b**	Whether clinical information and index test results were available to the assessors of the reference standard	N/A
Analysis	**14**	Methods for estimating or comparing measures of diagnostic accuracy	8-9
	**15**	How indeterminate index test or reference standard results were handled	8-9
	**16**	How missing data on the index test and reference standard were handled	N/A
	**17**	Any analyses of variability in diagnostic accuracy, distinguishing pre-specified from exploratory	8-9
	**18**	Intended sample size and how it was determined	5
**RESULTS**			
Participants	**19**	Flow of participants, using a diagram	N/A
	**20**	Baseline demographic and clinical characteristics of participants	10
	**21a**	Distribution of severity of disease in those with the target condition	N/A
	**21b**	Distribution of alternative diagnoses in those without the target condition	10
	**22**	Time interval and any clinical interventions between index test and reference standard	N/A
Test results	**23**	Cross tabulation of the index test results (or their distribution) by the results of the reference standard	N/A
	**24**	Estimates of diagnostic accuracy and their precision (such as 95% confidence intervals)	11, 14-15
	**25**	Any adverse events from performing the index test or the reference standard	10
**DISCUSSION**			
	**26**	Study limitations, including sources of potential bias, statistical uncertainty, and generalisability	16, 18-19
	**27**	Implications for practice, including the intended use and clinical role of the index test	20
**OTHER INFORMATION**			
	**28**	Registration number and name of registry	5-6
	**29**	Where the full study protocol can be accessed	21
	**30**	Sources of funding and other support; role of funders	21

For peer review only

STARD 2015

AIM

STARD stands for “Standards for Reporting Diagnostic accuracy studies”. This list of items was developed to contribute to the completeness and transparency of reporting of diagnostic accuracy studies. Authors can use the list to write informative study reports. Editors and peer-reviewers can use it to evaluate whether the information has been included in manuscripts submitted for publication.

EXPLANATION

A **diagnostic accuracy study** evaluates the ability of one or more medical tests to correctly classify study participants as having a **target condition.** This can be a disease, a disease stage, response or benefit from therapy, or an event or condition in the future. A medical test can be an imaging procedure, a laboratory test, elements from history and physical examination, a combination of these, or any other method for collecting information about the current health status of a patient.

The test whose accuracy is evaluated is called **index test.** A study can evaluate the accuracy of one or more index tests. Evaluating the ability of a medical test to correctly classify patients is typically done by comparing the distribution of the index test results with those of the **reference standard**. The reference standard is the best available method for establishing the presence or absence of the target condition. An accuracy study can rely on one or more reference standards.

If test results are categorized as either positive or negative, the cross tabulation of the index test results against those of the reference standard can be used to estimate the **sensitivity** of the index test (the proportion of participants *with* the target condition who have a positive index test), and its **specificity** (the proportion *without* the target condition who have a negative index test). From this cross tabulation (sometimes referred to as the contingency or “2x2” table), several other accuracy statistics can be estimated, such as the positive and negative **predictive values** of the test. Confidence intervals around estimates of accuracy can then be calculated to quantify the statistical **precision** of the measurements.

If the index test results can take more than two values, categorization of test results as positive or negative requires a **test positivity cut-off**. When multiple such cut-offs can be defined, authors can report a receiver operating characteristic (ROC) curve which graphically represents the combination of sensitivity and specificity for each possible test positivity cut-off. The **area under the ROC curve** informs in a single numerical value about the overall diagnostic accuracy of the index test.

The **intended use** of a medical test can be diagnosis, screening, staging, monitoring, surveillance, prediction or prognosis. The **clinical role** of a test explains its position relative to existing tests in the clinical pathway. A replacement test, for example, replaces an existing test. A triage test is used before an existing test; an add-on test is used after an existing test.

Besides diagnostic accuracy, several other outcomes and statistics may be relevant in the evaluation of medical tests. Medical tests can also be used to classify patients for purposes other than diagnosis, such as staging or prognosis. The STARD list was not explicitly developed for these other outcomes, statistics, and study types, although most STARD items would still apply.

DEVELOPMENT

This STARD list was released in 2015. The 30 items were identified by an international expert group of methodologists, researchers, and editors. The guiding principle in the development of STARD was to select items that, when reported, would help readers to judge the potential for bias in the study, to appraise the applicability of the study findings and the validity of conclusions and recommendations. The list represents an update of the first version, which was published in 2003.

More information can be found on http://www.equator-network.org/reporting-guidelines/stard.

For peer review only - http://bmjopen.bmj.com/site/about/guidelines.xhtml

BMJ Open

Clinical assessment of pelvic floor and abdominal muscles three months postpartum: An inter-rater reliability study

Journal:	*BMJ Open*
Manuscript ID	bmjopen-2021-049082.R1
Article Type:	Original research
Date Submitted by the Author:	23-Jun-2021
Complete List of Authors:	Vesting, Sabine; Göteborgs universitet Institutionen för neurovetenskap och fysiologi, Department of Health and Rehabilitation<br>Olsen, Monika Fagevik; Göteborgs universitet Institutionen för neurovetenskap och fysiologi, Department of Health and Rehabilitation<br>Gutke, Annelie; Göteborgs universitet Institutionen för neurovetenskap och fysiologi, Department of Health and Rehabilitation<br>Rembeck, Gun; University of Gothenburg School of Public Health and Community Medicine<br>Larsson, Maria; Göteborgs universitet Institutionen för neurovetenskap och fysiologi, Department of Health and Rehabilitation
**Primary Subject Heading**:	Obstetrics and gynaecology
Secondary Subject Heading:	Rehabilitation medicine
Keywords:	Maternal medicine < OBSTETRICS, Urinary incontinences < UROLOGY, REHABILITATION MEDICINE, Urogynaecology < GYNAECOLOGY

SCHOLARONE™
Manuscripts

I, the Submitting Author has the right to grant and does grant on behalf of all authors of the Work (as defined in the below author licence), an exclusive licence and/or a non-exclusive licence for contributions from authors who are: i) UK Crown employees; ii) where BMJ has agreed a CC-BY licence shall apply, and/or iii) in accordance with the terms applicable for US Federal Government officers or employees acting as part of their official duties; on a worldwide, perpetual, irrevocable, royalty-free basis to BMJ Publishing Group Ltd ("BMJ") its licensees and where the relevant Journal is co-owned by BMJ to the co-owners of the Journal, to publish the Work in this journal and any other BMJ products and to exploit all rights, as set out in our licence.

The Submitting Author accepts and understands that any supply made under these terms is made by BMJ to the Submitting Author unless you are acting as an employee on behalf of your employer or a postgraduate student of an affiliated institution which is paying any applicable article publishing charge ("APC") for Open Access articles. Where the Submitting Author wishes to make the Work available on an Open Access basis (and intends to pay the relevant APC), the terms of reuse of such Open Access shall be governed by a Creative Commons licence – details of these licences and which Creative Commons licence will apply to this Work are set out in our licence referred to above.

Other than as permitted in any relevant BMJ Author's Self Archiving Policies, I confirm this Work has not been accepted for publication elsewhere, is not being considered for publication elsewhere and does not duplicate material already published. I confirm all authors consent to publication of this Work and authorise the granting of this licence.

For peer review only

Clinical assessment of pelvic floor and abdominal muscles three months postpartum: An inter-rater reliability study

Sabine Vesting, MSc [1, 2] 0000-0002-6523-1833

Monika Fagevik Olsén, PhD [1, 3+4] 0000-0002-0207-4105

Annelie Gutke, PhD [1] 0000-0003-3484-9769

Gun Rembeck, PhD [5-7] 0000-0003-0097-5150

Maria E.H. Larsson, PhD [1, 8] 0000-0002-1225-9736

Author information:

1 Department of Health and Rehabilitation, Unit of Physiotherapy, Institute of Neuroscience and Physiology, Sahlgrenska Academy, University of Gothenburg, Gothenburg, Sweden.

2 Närhälsan Gibraltar Rehabilitation, Gothenburg, Sweden.

3 Department of Physical Therapy and Occupational Therapy, Sahlgrenska University Hospital, Sweden

4 Department of Gastrosurgical Research & Education, Sahlgrenska Academy, Gothenburg University, Gothenburg, Sweden.

5 Region Västra Götaland, Research and Development Centre Södra Älvsborg, Borås, Sweden

6 Region Västra Götaland, Regionhälsan Borås Youth Centre, Borås, Sweden

7 Primary Health Care, School of Public Health and Community Medicine, Institute of Medicine, Sahlgrenska Academy, University of Gothenburg, Gothenburg, Sweden.

8 Region Västra Götaland Education, Research and Development Primary Health Care, Gothenburg, Sweden.

Corresponding author: Sabine Vesting, MSc., University of Gothenburg, Box 430, 405 30 Gothenburg, Sweden, mobile phone 0046-722095484, sabine.vesting@gu.se

Word count: Main document (4580), Abstract (299), Tables (5)

For peer review only

ABSTRACT

Objectives: Evaluation of the inter-rater reliability of clinical assessment methods for pelvic floor muscles and diastasis recti abdominis postpartum.

Design: A multi-center inter-rater reliability study.

Setting: Three primary care rehabilitation centers in Sweden.

Participants: A total of 222 participants were recruited via advertising at Swedish maternity care units and social media. Eligibility for participation included female gender, ≥18 years, at maximum three months after childbirth. Exclusion criteria were chronic pelvic girdle pain and/or low back pain and/or pelvic floor tear grade III/IV. At each center, 2 physiotherapists, with training and experience in pelvic floor assessment, assessed the 222 women according to a standardized protocol in random order.

Outcome measures: Inter-rater reliability of the assessment of pelvic floor muscle function (involuntary and voluntary contraction and voluntary relaxation) and diastasis recti abdominis (width, depth, and bulging).

Results: Vaginal palpation of maximal voluntary contraction revealed a kappa value of 0.69 [95% CI 0.62; 0.76]. Assessments of involuntary contraction, and voluntary relaxation yielded inconsistent results, with slight-to-moderate weighted kappa values ranging from 0.10–0.51. After two months of training in applying this method, diastasis recti abdominis width measured at the umbilicus by caliper yielded an ICC value of 0.83 [95% CI 0.76; 0.87]. Assessments of diastasis recti abdominis depth and bulging showed moderate kappa values, with reservation for some inconsistency between the centers.

Conclusions: Vaginal palpation of pelvic floor muscle strength is a reliable method for the postpartum muscle assessment. Additional research is needed to identify reliable assessment method for other pelvic floor muscle functions like involuntary contraction and voluntary

For peer review only - http://bmjopen.bmj.com/site/about/guidelines.xhtml

relaxation. With some training, a caliper is a reliable instrument for measuring the postpartum diastasis recti abdominis width. This study provides novel thoughts about how to measure diastasis recti abdominis depth and bulging.

Trial registration: Registered at clinicaltrial.gov, October 2018, ClinicalTrials.gov Identifier NCT03703804.

Keywords: childbirth, caliper, diastasis recti abdominis, inter-rater, palpation, pelvic floor muscles

STRENGTHS AND LIMITATIONS

1. This study provides a detailed description of clinically applicable assessment methods for postpartum muscle assessment in primary care.
2. This study is raising novel thoughts about the question how diastasis recti abdominis depth and bulging could be assessed postpartum.
3. The need of training and experience in the clinical assessment of the diastasis recti abdominis was underestimated before start of the study.
4. The used assessment methods were not validated for example by comparing the results with technical devices like ultrasound and/or pressure measurement.
5. The assessment methods were tested at three primary care rehabilitation centers, adding training and experience and maybe even continuity as important aspects for a reliable assessment

INTRODUCTION

Both the pelvic floor and the abdominal muscles are part of the stabilization system for the pelvic and spine[1] as well as the continence system.[2] Pregnancy and childbirth cause alterations in these muscle groups. During a vaginal delivery, the pelvic floor muscles overstretches up to

For peer review only - http://bmjopen.bmj.com/site/about/guidelines.xhtml

three times.[3] It takes approximately six months until muscles, nerves and the connective tissue are recovered from a vaginal delivery.[4,5] Childbirth is associated with pelvic floor traumas, such as perineal tears and levator ani injuries, which can lead to incontinence, pelvic organ prolapse, and decreased quality of life.[6,7] Another postpartum muscle alteration is a persistent separation of the two parts of the rectus abdominis, termed a diastasis recti abdominis. At 12 months postpartum, 33% of women exhibit a diastasis recti abdominis greater than the width of two fingers.[8] A diastasis recti abdominis is reportedly correlated with impaired quality of life, negative body image, and abdominal pain.[9,10]

Women are increasingly seeking help and advice regarding postpartum muscle alterations from physiotherapists at primary health care centers. Physiotherapists use various methods to assess the pelvic floor muscles and diastasis recti abdominis after pregnancy,[11,12] however, there is currently no gold standard. Clinically, vaginal palpation and observation are common methods to assess different pelvic floor muscle functions after childbirth. The International Continence Society defined that the pelvic floor muscle can be involuntarily and voluntarily contracted and can also be voluntarily relaxed.[13] However, there are no standardized assessment protocols or rating scales in the postpartum care, yet. One study reported the use of a Delphi scheme to identify the optimal protocol for assessing different pelvic floor muscle functions and tested their inter-rater reliability,[14] but these assessments were not performed in postpartum women.

Ultrasound assessment is the most reliable and valid method for measurement of the diastasis recti abdominis width.[15] However, most women who are concerned about their diastasis recti abdominis seek help at primary healthcare centers, where ultrasound is seldom available. About 96% of American physiotherapists specialized in women's health assess the diastasis recti abdominis width using the finger-width method,[8,16] which is imprecise due to finger-width variations[17] and has weaker inter-rater reliability than instrumental assessment

For peer review only - http://bmjopen.bmj.com/site/about/guidelines.xhtml

methods.[15] Less than 2% of American physiotherapists use calipers for the assessment of postpartum women.[16] Diastasis recti abdominis width assessment using a caliper is reported to be nearly as accurate as ultrasound assessment,[15,18] although the inter-rater reliability of this method has been tested in just one study.[19]

An experimental study shows that the tendon between the two parts of the rectus abdominis—the linea alba— has less tension in a curl-up movement in women with diastasis recti abdominis. [20] These findings of Lee and Hodges were strengthened by a further study measuring the tension and stiffness in the linea alba.[21] Both research groups argue that the tension in the linea alba is an important factor for the load transfer in the core and might be one of the key components to explain why women with diastasis recti abdominis experience functional impairments. However, these studies were experimental, and their hypothesis is not validated yet. The assessments in these studies were performed by ultrasound [20] and shear-wave elastography.[21] For clinical practice, there are currently no reliable and clinically applicable assessment methods or rating scales for linea alba tension or stiffness. Hypothetical, the tension in the linea alba could be palpated, the inability to control intraabdominal pressure could be observed as diastasis recti abdominis bulging.[22]

In the present study, we aimed to evaluate the inter-rater reliability of different aspects of the clinical assessment of pelvic floor muscle and diastasis recti abdominis using observation, calipers, and palpation at three months postpartum.

MATERIAL AND METHODS

Study design and subjects

In total, 222 women from the Region Västra Götaland, Sweden were included to this reliability study. Based on the guidelines of Ko & Li, we aimed to assess at least 30 participants at each center.[23] Assessments were conducted at three primary care rehabilitation centers. The study

For peer review only - http://bmjopen.bmj.com/site/about/guidelines.xhtml

was registered at clinicaltrial.gov, October 2018, with the Identifier NCT03703804 and at the Research and Development Centre Gothenburg and Södra Bohuslän with the registration number 243071. All included women gave their written informed consent to participate. The study protocol was approved by the Swedish ethical review authority in Gothenburg in April 2018 (Dnr 088-18) and registered with the local data protection service, Närhälsan Gothenburg, in January 2019. This study is in line with the Helsinki declaration and the STARD (Standards for Reporting Diagnostic accuracy studies) checklist was used to report this study about assessment methods.

The women were invited to participate via advertising at antenatal and childcare centers, and social media. Inclusion criteria were age of ≥18 years, vaginal delivery or caesarean section within the past three months, and the ability to understand and respond in Swedish. Exclusion criteria were chronic pelvic girdle pain and/or low back pain (defined as self-reported pelvic or low back pain for over three months, not related to the last pregnancy) and/or pelvic floor tear grade III/IV.

The participants were contacted and booked for assessment at one of the three primary care rehabilitation centers in the Västra Götaland region between eight to twelve weeks after giving birth. Assessments were performed by six physiotherapists—two at each primary care rehabilitation center. Prior to the assessments, the participants completed a questionnaire about their age, body mass index (BMI), mode of delivery, number of delivered children, self-reported pelvic floor tears, most recent baby's birth weight, and the birth weights of previous children (if applicable).

The assessing physiotherapists had each completed a four-day (or longer) course in pelvic floor muscle assessment and treatment methods. The physiotherapists at primary care rehabilitation center 1 had two and nine years' experience in pelvic floor muscle assessment at start of the study, the physiotherapists at primary care rehabilitation center 2 had one and three

For peer review only - http://bmjopen.bmj.com/site/about/guidelines.xhtml

years' experience and the physiotherapists at primary care rehabilitation center 3 had both one year experience in pelvic floor muscle assessment at start of the study. All six physiotherapists work at primary care rehabilitation centers, part- of fulltime with women´s health. They had experience in palpating the diastasis recti abdominis with the finger-width method. However, similar to the low numbers of American physiotherapists using the caliper,[11] caliper measurement was new to all rates as it is unusual in Sweden and diastasis recti abdominis assessment is not part of the Swedish physiotherapy education. Therefore, all included physiotherapists were novices at measuring the diastasis recti abdominis by caliper, and on using the rating scales for depth and bulging. During the design phase of this study, four hours of training in diastasis recti abdominis measurement was planned.

Due to an insecurity among the physiotherapists in assessing and measuring the diastasis recti abdominis in the beginning of the study, the first two months of the study (63 measurements) were used for training and validation of the right technique for using the caliper and assessing depth and bulging. After these two months, the physiotherapists at all centers underwent additional training, comparing their results and discussing their technique. The whole validation process and the final assessment protocol after validation is available in Supplement 1.

Patient and public involvement

Patients and the public were not actively involved in the initial design of the study. However, the application of the caliper was tested in a pilot trial and the opinions of the participants about the application were considered for the design of the final assessment protocol (Supplement 1).

Clinical assessment of the pelvic floor muscles

The pelvic floor muscles were assessed with the participant in supine position on a flat bench, with the legs flexed and slightly abducted. The participants had a pillow under the head. The

For peer review only - http://bmjopen.bmj.com/site/about/guidelines.xhtml

assessed pelvic floor muscle functions were involuntary and voluntary contraction (by observation and vaginal palpation) and maximal voluntary contraction, pelvic floor muscle endurance and voluntary relaxation (by vaginal palpation). The detailed assessment protocol is shown in Supplement 2.

Clinical assessment of the diastasis recti abdominis

The assessment of the diastasis recti abdominis was conducted in supine position on a flat bench, without a pillow. The participants were in hook-lying position with their arms resting at their sides. The physiotherapists assessed diastasis recti abdominis width using an electronic digital caliper (150 mm, carbon fiber, accuracy ±0.2 mm, 24 se Sverige AB, Kalmar, Sweden). Diastasis recti abdominis depth and bulging were assessed by observation and palpation. All final measures or ratings were results of single assessments to avoid a fatigue effect. The step-wise explanation of caliper application and assessment of diastasis recti abdominis is explained in Supplement 1.

Upon completion of the assessment, the participants rested for 30 minutes in a sitting or lying position. After the 30-minute rest, the second physiotherapist conducted the same assessment as described above. The two physiotherapists at each site were assessing in random order, were blinded to each other's findings, and not allowed to talk about their assessments. The physiotherapist were also blinded to the participants background information.

Statistical analysis

Statistical analyses were performed using IBM SPSS statistical package version 25 (SPSS Inc., Chicago, IL). Descriptive statistics are presented as mean and standard deviation (SD)for ratio data, and as number and percentage for nominal and ordinal data. To calculate statistically significant differences between the three rehabilitation centers, we used the one-way ANOVA

For peer review only - http://bmjopen.bmj.com/site/about/guidelines.xhtml

test for ratio data, and the Kruskal-Wallis test for ordinal data and chi-squared test for categorical data. A p value of ≤0.05 was regarded as statistically significant.

All pelvic floor muscles functional measures were rated on ordinal scales, except for pelvic floor muscle endurance and involuntary contraction by palpation. Diastasis recti abdominis depth was also rated on an ordinal scale. Ratings on ordinal scales were evaluated by Cohen's weighted kappa values. Pelvic floor muscle endurance, involuntary contraction by palpation, and diastasis recti abdominis bulging were rated on nominal scales, and these ratings were evaluated by Cohen's kappa values. For interpretation of kappa values, we used the categories of Landis and Koch: <0.2, slight; 0.21–0.40, fair; 0.41–0.60, moderate; 0.61–0.80, substantial; and 0.81–1.0, almost perfect agreement.[24] Percentage agreement was calculated and presented for all nominal and ordinal data, and <60% agreement was defined as faulty agreement.[25]

For assessment of diastasis recti abdominis width, a continuous scale (in mm) was used. To evaluate the inter-rater reliability of the assessments on a continuous scale, we calculated the intraclass correlation coefficient (ICC) and 95% confidence interval (CI). ICC values were calculated in SPSS based on absolute agreement and a 2-way mixed effects model. ICC values of <0.50 indicate poor reliability, 0.50–0.75 indicate moderate reliability, 0.75–0.90 good reliability, and values of >0.90 indicate excellent reliability [23].

To further evaluate reliability, we calculated the standard error of measurements ($SEM = SD \times \sqrt{(1 - ICC)}$), which represent the typical error in a single measurement and the minimal detectable change ($MDC = SEM \times 1.96 \times \sqrt{2}$). For calculation of the standard error of measurements (SEM), we used the standard deviation (SD) from the scores of all subjects. SEM and MDC values are presented in mm.

For peer review only - http://bmjopen.bmj.com/site/about/guidelines.xhtml

RESULTS

A total of 222 women were assessed, with measurements conducted from September 2018 through February 2020. The mean age of the participants was 33.1 years (SD ±3.3) and the majority of women (61%) had delivered one child (Table 1). The participating women at primary care rehabilitation center 3 were significantly younger than the women at primary care rehabilitation center 2 and had significantly more children than the participants at primary care rehabilitation centers 1 and 2.

For peer review only

For peer review only - http://bmjopen.bmj.com/site/about/guidelines.xhtml

Table 1. Characteristics of the participating women at three months postpartum (N=222)

	Total (n = 222)	Primary care rehabilitation center 1 (n = 90)	Primary care rehabilitation center 2 (n = 103)	Primary care rehabilitation center 3 (n = 29)	*p* [a]
	Mean ±SD	*Mean* ±SD	*Mean* ±SD	*Mean* ±SD	
**Age in years**	33.1 ±3.3	32.6 ±3.5	33.8 ±2.9	32.0 ±3.8	**0.01**
**BMI**	24.5 ±3.0	24.4 ±3.1	24.3 ±2.9	25.1 ±3.2	0.42
**Neonatal birth weight**	3574.5 ±507.0	3517.7 ±533.7	3604.7 ±489.2	3641.1 ±484.0	0.59
	n (%)	n (%)	n (%)	n (%)	
**Delivery mode**					0.48
C-section	28 (13)	9 (10)	16 (16)	3 (10)	
Vaginal delivery	194 (87)	81 (90)	87(85)	26 (90)	
**Number of children**					**<0.01**
1	137 (61)	51 (57)	75 (73)	11 (30)	
2	74 (33)	33 (37)	26 (25)	15 (50)	
3	9 (4)	7 (6)	1 (1)	1 (3)	
4 or more	3 (1)	0 (0)	1 (1)	2 (7)	
**Self-reported perineal tear**					0.33
No tear	54 (24)	24 (27)	23 (22)	7 (24)	
First-degree perineal tear	52 (23	16 (18)	24 (23)	12 (41)	
Second-degree perineal tear /episiotomy	74 (33)	33 (38)	35 (34)	6 (20)	

n, number; BMI, body mass index; SD, standard deviation.

a) *p* value by one-way ANOVA test for ratio data, Kruskal-Wallis test for ordinal data and chi-squared test for categorical data.

Clinical assessment of the pelvic floor muscles

The evaluation of maximal voluntary contraction showed substantial agreement (weighted kappa value, 0.69 [95 % CI 0.62 to 0.76]), and assessment of pelvic floor muscle endurance showed moderate agreement (kappa value, 0.49 [95% CI 0.37 to 0.61]) (Table 2). Seven participants (3.3%) were excluded from the analyses of maximal voluntary contraction and

For peer review only - http://bmjopen.bmj.com/site/about/guidelines.xhtml

pelvic floor muscle endurance due to incorrect pelvic floor muscle contraction (straining). Assessment of voluntary contraction by observation showed moderate agreement, with a weighted kappa of 0.45 [95% CI 0.28 to 0.62]. Among all assessments, about 89% were rated as “perineal inward movement”.

Table 2. *Results of the clinical assessment of pelvic floor muscles via observation and vaginal palpation*

Parameters	Total group (n = 222)		Primary care rehabilitation center 1 (n = 90)		Primary care rehabilitation center 2 (n = 103)		Primary care rehabilitation center 3 (n = 29)	
	Kappa [95% CI]	PA %	Kappa [95% CI]	PA %	Kappa [95% CI]	PA %	Kappa [95% CI]	PA %
**Voluntary contraction**								
**Observation**	0.45 [0.28; 0.62]	90	0.40 [0.11; 0.70]	91	0.55 [0.33; 0.77]	90	0.29 [−0.13; 0.70]	86
**Vaginal palpation**								
**MVC**[b]	0.69 [0.62; 0.76]	71	0.70 [0.58; 0.82]	77	0.59 [0.46; 0.71]	67	0.67 [0.50; 0.84]	62
**PFM Endurance**[b]	0.49 [0.37; 0.61]	74	0.68 [0.53; 0.83]	84	0.27 [0.09; 0.45]	65	0.55 [0.20; 0.90]	83
**Involuntary contraction**								
**Observation**	0.10 [−0.02; 0.22]	57	0.20 [−0.01; 0.42]	77	0.11 [−0.05; 0.27]	48	0.38 [0.10; 0.67]	68
**Vaginal palpation**	0.51 [0.37; 0.65]	85	0.26 [-0.04; 0.56]	87	0.47 [0.25; 0.69]	85	0.47 [0.14; 0.80]	75
**Voluntary relaxation**								
**Vaginal palpation**	0.26 [0.15; 0.37]	57	0.30 [0.16; 0.50]	63	−0.08 [−0.23; 0.07]	45	0.56 [0.07; 1.02]	89

n, number; CI, confidence interval; PA, percentage agreement; MVC, maximal voluntary contraction; PFM, pelvic floor muscle.

b) reduced number of participants due to incorrect pelvic floor muscle contraction (straining): total group (*n* = 215), primary care rehabilitation center 1 (*n* = 88), primary care rehabilitation center 2 (*n* = 98), primary care rehabilitation center 3 (*n* = 29).

For peer review only

For peer review only - http://bmjopen.bmj.com/site/about/guidelines.xhtml

The assessment of involuntary contraction by observation exhibited slight agreement (weighted kappa value, 0.10 [95% CI −0.02 to 0.22]). About 70% of participants were rated as “downward movement”, and 9–11% as upward movement. Fair-to-moderate kappa values were found for evaluation of involuntary contraction by palpation. Over 80% of participants were rated as “absence of correct contraction”.

Ratings of voluntary relaxation showed large variations between different primary care rehabilitation centers, with weighted kappa values ranging from −0.08 to 0.56. The application of the scale significantly differed between primary care rehabilitation center 3 and primary care rehabilitation centers 1 + 2. At primary care rehabilitation center 3, the physiotherapists rated 25 of 29 assessments as showing complete voluntary relaxation. In contrast, at primary care rehabilitation centers 1 + 2, 10–12% of participants were rated as absent voluntary relaxation, 66% as partly relaxed, and 20–24% as complete voluntary relaxation. Primary care rehabilitation center 2 showed a negative kappa value of −0.08, indicating an agreement worse than expected or no agreement.[25]

Clinical assessment of the diastasis recti abdominis

Diastasis recti abdominis width, depth, and bulging were assessed in 159 women. The mean diastasis recti abdominis widths at three months postpartum was 25.9 mm at the umbilicus (Table 3). The diastasis recti abdominis widths measured at rehabilitation center 3 were significantly wider at all three measure points compared to rehabilitation centers 1 and 2 (p value<0.01). The DRA widths measured at the umbilicus was significantly wider in women with more than one child at rehabilitation center1 (p value= 0.04) but not at rehabilitation center 2+3.

For peer review only - http://bmjopen.bmj.com/site/about/guidelines.xhtml

Table 3. *Diastasis recti abdominis width at three months postpartum (in mm) measured with a caliper*

	Above the umbilicus<br>*Mean* ±SD<br>95% CI	At the umbilicus<br>*Mean* ±SD<br>95% CI	Below the umbilicus<br>*Mean* ±SD<br>95% CI
Total group (n = 159)	22.0 ±7.4<br>[20.9; 23.2]	25.9 ±7.2<br>[24.8; 27.1]	19.6 ±7.3<br>[18.4; 20.7]
Primipara (n=97)	20.73 ±6.0<br>[19.5;21.9]	24.3±5.7<br>[23.1; 25.4]	19.0 ±9.9<br>[17.6; 20.4]
Multipara (n= 62)	24.1±8.9<br>[21.9; 26.4]	28.7±8.5<br>[26.5; 30.8]	20.6±7.9<br>[18.6; 22.6]
Primary care rehabilitation center 1 (n = 61)	19.8 ±5.3<br>[18.5; 21.2]	24.1 ±6.5<br>[22.5; 25.8]	14.8 ±5.8<br>[13.3; 16.2]
Primipara (n=35)	18.7 ±4.5<br>[17.2; 20.3]	22.6 ±5.1<br>[20.9; 24.4]	13.7±5.1<br>[12.0; 15.5]
Multipara (n= 26)	21.3 ±6.0<br>[18.8; 23.9]	26.1 ±7.6<br>[23.1; 29.5]	16.2 ±6.4<br>[13.5; 18.8]
Primary care rehabilitation center 2 (n = 69)	20.6 ±4.6<br>[19.5; 21.7]	24.7 ±5.2<br>[23.5; 25.9]	21.1 ±4.6<br>[20.0; 22.2]
Primipara (n=51)	20.4 ±4.5<br>[19.1; 21.7]	24.3 ±4.6<br>[23.0; 25.6]	21.3 ±4.2<br>[20.1; 22.5]
Multipara (n= 18)	21.3 ±4.9<br>[18.9; 23.8]	25.9±6.6<br>[22.6; 29.1]	20.4 ±5.8<br>[17.6; 23.3]
Primary care rehabilitation center 3 (n = 29)	29.9 ±10.9<br>[25.8; 34.1]	32.8 ±9.0<br>[29.3; 36.2]	26.1 ±9.0<br>[22.7; 29.6]
Primipara (n=11)	28.6 ±9.4<br>[22.3; 34.9]	29.5 ±9.2<br>[23.3; 35.7]	25.0 ±11.0<br>[17.6; 32.4]
Multipara (n= 18)	30.7 ±11.9<br>[24.8; 36.6]	34.8 ±8.5<br>[30.5; 39.0]	26.9 ±7.8<br>[23.0; 30.7]

n, number; SD, standard deviation.

For peer review only

The measurement of diastasis recti abdominis width with a caliper showed good reliability when measured at the umbilicus and 4.5 cm below the umbilicus, and moderate reliability at 4.5 cm above the umbilicus (Table 4). For the total group, the SEM was between 4.05–4.75 mm, and the MDC was 11.23–13.17 mm. Sub-analysis of the different primary care rehabilitation centers revealed two negative outliers. At primary care rehabilitation center 2, assessment at 4.5 cm below the umbilicus showed an ICC value of 0.51 [95% CI: 0.20 to 0.70], which is at the lower boundary of the definition for moderate reliability. Assessment at 4.5 cm above the umbilicus at primary care rehabilitation center 3 showed much lower ICC values compared to the other values. An ICC value of 0.40 indicates low reliability.

Table 4. *Results of the clinical assessment of diastasis recti abdominis width with a caliper (in mm)*

Parameters	Total group (*n* = 159)	Primary care rehabilitation center 1 (*n* = 61)	Primary care rehabilitation center 2 (*n* = 69)	Primary care rehabilitation center 3 (*n* = 29)
	ICC [95% CI] SEM; MDC	ICC [95% CI] SEM; MDC	ICC [95% CI] SEM; MDC	ICC [95% CI] SEM; MDC
**Above the umbilicus**	0.73 [0.63; 0.80] 4.75; 13.17	0.78 [0.63; 0.87] 5.32; 14.75	0.60 [0.36; 0.75] 3.46; 9.59	0.40 [−0.32; 0.72] 8.30; 23.01
**At the umbilicus**	0.83 [0.76; 0.87] 4.05; 11.23	0.85 [0.75; 0.91] 3.29; 9.12	0.62 [0.39; 0.77] 4.34; 12.03	0.82 [0.61; 0.91] 4.93; 13.66
**Below the umbilicus**	0.80 [0.72; 0.85] 4.40; 12.20	0.75 [0.58; 0.85] 3.64; 10.09	0.51 [0.20; 0.70] 4.03; 11.17	0.74 [0.43; 0.88] 6.16; 17.07

n, number; ICC, intraclass correlation coefficient; CI, confidence interval; SEM, standard error of measurements; MDC, minimal detectable change.

The assessment of diastasis recti abdominis depth showed fair-to-moderate weighted kappa values ranging from 0.34–0.43 (Table 5). In the assessment of the diastasis recti abdominis depth, 21% were assessed as “good resistance at all points”, 67% as “resistance

For peer review only - http://bmjopen.bmj.com/site/about/guidelines.xhtml

in the depth", and 12% as "bottomless". Assessment of diastasis recti abdominis bulging in the 3-step sit-up test showed moderate agreement (kappa value, 0.51 [95% CI 0.29 to 0.73]). The values primary care rehabilitation center 2 and 3 were lower than at primary care rehabilitation center and showed wide ranges in the 95% CI. Among the participants, about 81% were rated as "no bulging", 12% as "diastasis recti abdominis bulging", and 7% as "cannot assess".

Table 5. *Results of the clinical assessment of diastasis recti abdominis depth and bulging via observation and palpation*

	Total Group (*n* = 159)		Primary care rehabilitation center 1 (*n* = 61)		Primary care rehabilitation center 2 (*n* = 69)		Primary care rehabilitation center 3 (*n* = 29)	
	Kappa [95% CI]	PA %	Kappa [95% CI]	PA %	Kappa [95% CI]	PA %	Kappa [95% CI]	PA %
**Depth**	0.43 [0.29; 0.56]	69	0.37 [0.15; 0.59]	70	0.34 [0.11; 0.58]	71	0.36 [0.04; 0.69]	62
**Bulging**[c]	0.51 [0.29; 0.73]	88	0.77 [0.52; 1.02]	94	0.35 [0.04; 0.66]	83	0.36 [-0.16; 0.88]	88

n, number; CI, confidence interval; PA, percentage agreement.

c) The physiotherapists had the rating option of "cannot assess", and these assessments were excluded: Total group (*n*= 137); primary care rehabilitation center (*n* = 52); primary care rehabilitation center 2 (*n*= 59); and primary care rehabilitation center 3 (*n* = 26).

DISCUSSION

The main findings of this study are that physiotherapists managing women's health in primary care have reliable methods available to assess voluntary pelvic floor muscle contraction by vaginal palpation and measure diastasis recti abdominis width by caliper three months postpartum. On the other hand, the assessment of involuntary contraction by observation and voluntary relaxation had kappa values with slight-to-fair agreement. Data with such low agreement are not useful for clinical practice or research.[25] Further investigations are needed to improve the clinical applicability and reliable assessment of these functions.

For peer review only - http://bmjopen.bmj.com/site/about/guidelines.xhtml

Clinical assessment of the pelvic floor muscles

Our present results showed weighted kappa values of 0.59–0.70 for the assessment of maximal voluntary contraction, which are higher compared to previous studies.[26-28] One explanation could be differences between the rating scales used for this muscle function. For example, in two prior studies,[26,27] only the squeezing and not the lifting component of the contraction was rated. Devreese *at al* discussed the importance of the lifting component of pelvic floor muscle strength, showing that incontinent women showed less inward movement than continent women.[29] Furthermore, the differences to our results could also be related to differences in the study population and smaller sample sizes in previous studies.

The assessment of pelvic floor muscle endurance showed moderate reliability. However, there were several inconsistencies in the pelvic floor muscle endurance data. Assessment of pelvic floor muscle endurance at primary care rehabilitation center 2 showed only fair reliability, with a kappa value of 0.27 [95% CI 0.09 to 0.45]. Our present results are lower compared with the findings of Devreese *et al*..[29] Notably, in the study of Devreese *et al*, a contraction longer than 10 seconds was rated as positive rather than a contraction longer than 30 seconds, as in the present study. Additionally, their study population was older and not three months postpartum. It may be more difficult to assess the exact point of time when the contraction is subsiding in a longer time period. It is also possible that postpartum women have, on average, a weaker contraction, making it more difficult to assess pelvic floor muscle endurance. Further research is needed to decide whether a pelvic floor muscle endurance of 10 seconds or 30 seconds is more clinically relevant.

Voluntary contraction via observation showed fair-to-moderate weighted kappa values. A prior MRI study reported that the average inward movement of the perineum is about 1 cm while sitting, and it is more than 2 cm in the supine position, according to Kegel in 1952.[30] It could be assumed that this large movement would be easy to observe, and a higher kappa

For peer review only - http://bmjopen.bmj.com/site/about/guidelines.xhtml

value was expected. A previous study reported high inter-rater reliability in the observation of inward perineum movement.[14] Correspondingly, another study showed that inward perineal movement could be observed with a kappa value of 0.91 among continent women, and 0.93 among incontinent women- [29]

As factors other than pelvic floor muscle strength may contribute to urinary leakage postpartum,[31] it is important to assess other aspects of pelvic floor muscle function. Unfortunately, in our present study, we did not find a reliable method for assessing involuntary contraction by observation, and we demonstrated inconsistent findings for the assessment of involuntary contraction by palpation. Accordingly, another study reported only fair inter-rater reliability for the assessment of involuntary contraction by observation and palpation.[14] Further studies are needed to develop improved methods for the assessment and rating of these pelvic floor muscle functions in clinical practice.

When prescribing postpartum pelvic floor muscle training, it must be considered that some women have overactive, and possibly painful pelvic floor muscles. [32] It remains unclear whether women with hypertonic pelvic floor muscles should be advised to do pelvic floor muscle training. In clinical practice, physiotherapists recommend an individualized approach. Our present results showed that the rating of voluntary relaxation had slight-to-fair inter-rater reliability. Slieker-ten *et al.* [14] reported similar findings. Another study used a five-step rating scale for relaxation after contraction, and reported a correlation of 0.34 between two raters.[32] Even, Slieker-ten *et al.* recommend the addition of more rating steps to the scale, e.g., incomplete relaxation in their discussion. Regardless of whether women with hypertonic pelvic floor muscles need more support in pelvic floor muscle training or the recommendation of no pelvic floor muscle training, there remains a need for better methods of assessing this condition.

For peer review only - http://bmjopen.bmj.com/site/about/guidelines.xhtml

Clinical assessment of the diastasis recti abdominis

Our results from the present study showed moderate-to-good reliability in measuring diastasis recti abdominis width using a caliper, after two months of training and calibrating of the method. The characteristics of the diastasis recti abdominis at three months postpartum measured with the caliper at rehabilitation centre 1+2 were comparable with the diastasis recti abdominis characteristics after pregnancy measured by ultrasound,[33] indicating these values are true values for this population. However, the two months of training and calibrating of the method via discussion and additional training were important components of this study. One bias identified in our additional training was that the accurate head lift of just 2–3 cm was an important factor. This observation is supported by the study of Mota *et al*. [34] which showed that the distance between the two parts of the rectus abdominis decreased during a sit-up movement.

There was some inconsistencies in the data, with two outliers at primary care rehabilitation centers 2 + 3. The SEM and MDC were higher at primary care rehabilitation center 3 compared to primary care rehabilitation centers 1 + 2 (Table 4). The MDC measured at 4.5 cm above the umbilicus was over 2 cm, raising doubt about whether these results have any clinical relevance. Taking into concern, that the measured DRA width at primary care rehabilitation centers 3 are significantly wider than at primary care rehabilitation centers 1+2, seen in both primi- and multiparous women, questions about if it is more difficult to measure a wider diastasis recti abdominis or if the measures are not accurate have to been raised. Comparing the results from rehabilitation center 3 with the normal values for primiparous women of Mota el al. [33], there is an indication for residual problems with test performance even after retraining. At primary care rehabilitation center 3, fewer than 30 participants were recruited during the study period of over one year. These results indicate that in addition to training and experience, some continuity in measuring the diastasis recti abdominis width with a caliper is necessary for reliable assessment.

For peer review only - http://bmjopen.bmj.com/site/about/guidelines.xhtml

The SEM in our present study was about 4–5 mm and the MDC was about 11-13 mm, which is higher than in the comparable study of Benjamin *et al.*[19] However, just two participants showed an inter-recti distance greater than 22 mm in their study, in our study the mean diastasis recti abdominis width was 25.9 mm. Furthermore, Benjamin *et al* is not discussing how much experience and training the two investigating physiotherapists had in measuring diastasis recti abdominis width with a caliper.

We found fair-to-moderate weighted kappa values for the assessment of diastasis recti abdominis depth. To the best of our knowledge, there is no other study with which to compare these results. About 63–66% of the assessments were rated as "resistance in the depth". The investigating physiotherapists hypothesized that "resistance in the depth" was felt and assessed as soon as the participants did not activate their deeper abdominal muscles during the head lift. Accordingly, Lee and Hodges[20] described the increased tension in the linea alba caused by activation of the deep abdominal muscles. If this is true, the assessment of diastasis recti abdominis depth is an assessment of the tension in the linea alba due to *musculus transversus abdominis* activation during a head lift. A next step could be to correlate the depth with the width- with the hypothesis that transversus abdominis activation increases the width[34] and decreases the depth. Future studies must more precisely define the pre-activation of the deep abdominal muscles in the assessment of diastasis recti abdominis depth. Similar considerations have to be raised about abdominal muscle pre-activation and diastasis recti abdominis bulging during a sit-up curl. Before implications of this methods to clinical practice, further validation of the assessments of diastasis recti abdominis depth and bulging, for example a correlation between ultrasound/ shear-wave elastography and palpation, is needed

Strengths and limitations

One strength of this study was the large sample size and the quantity of different aspects of muscle function. A comparable study assessing different aspects of pelvic floor muscle function

For peer review only - http://bmjopen.bmj.com/site/about/guidelines.xhtml

in women with and without pelvic floor disorders included only 41 participants.[14] A review about diastasis recti abdominis assessment methods included studies with 20–106 participants, and these studies only examined diastasis recti abdominis width.[15] Another strength of this study was that we were able to perform the same tests at three different centers in different parts of west Sweden. This makes our results transferable for different physiotherapists using the same methods.

The present study also had several limitations. One was that we lacked access to the participants' delivery records. Thus, the descriptive statistics and the calculate statistically significant differences between the three rehabilitation centers regarding age, BMI, mode of delivery, pelvic floor tearing, and highest birth weight were based on self-reported data from the participants. Another issue was the uneven cell distribution seen in over 50% of the rating scales tested in this study. In the literature, it is controversial whether a low kappa value can be explained by uneven cell distribution or low prevalence of a condition. [35]

Another limitation but also a strength of this study was the initial assumption that four hours of training in measuring the diastasis recti abdominis width with a caliper would be enough training for the six assessing physiotherapists. Due to this misjudgment of complexity using this method, a total of 63 measurements had to be excluded. On the other hand, many different obstacles, and sources of error for the measurements could be found through this initial phase and the added training. Due to the troubleshooting around the right application and procedure while measuring the diastasis recti abdominis with a caliper, the procedure of this study might not 100% comparable with other studies. However, there are just a few existing studies measuring by caliper[18,36-39], several measurement positions like resting, in a crunch position and in a modified curl-up were used, no consent about the right application method is found yet. In the training sessions to this study, we found it almost impossible to measure in resting, even if other studies[38,39] described the measurement in resting as reliable methods. At

For peer review only - http://bmjopen.bmj.com/site/about/guidelines.xhtml

the same time, we could see that as higher the head lift/shoulder lift as less widths was measurable which is in line with a study showing that an abdominal crunch is narrowing the inter-recti distance.[34] Our intention was to find an active position without missing the actual width of the diastasis recti abdominis. It would have been even better to validate these caliper measurements against ultrasound measurements. However, the aim of this study was to evaluate clinical applicable methods, which are easy to use and inexpensive tools for daily practice. Validation via ultrasound could be a next step.

Future research

For a next step, we must investigate the extent to which these values are clinically relevant for postpartum women—for example, if the assessment outcomes are associated with pain and dysfunction. Furthermore, we must determine a cut-off point for diastasis recti abdominis severity relative to pain and dysfunction. It will also be important to determine what training advice should be given to postpartum women based on the results of these examinations.

There also remains a need for further research on how to assess and rate involuntary contraction by observation and palpation, and voluntary relaxation, in the clinical assessment of women after pregnancy. Furthermore, additional research is needed regarding the assessment of the diastasis recti abdominis, in terms of depth and bulging of the linea alba in movement.

CONCLUSION

Women are increasingly requesting assessment of their pelvic floor muscles and diastasis recti abdominis after pregnancy. Our present results revealed moderate-to-substantial reliability for the assessment of maximal voluntary contraction and pelvic floor muscle endurance by vaginal palpation, considering both the lifting and squeezing component of the contraction. Furthermore, diastasis recti abdominis width can be measured by caliper, with a standard error

For peer review only - http://bmjopen.bmj.com/site/about/guidelines.xhtml

of measurements of 4–5 mm and a minimal detectable change of about 1.2 cm. However, assessment using this instrument requires some training and experience.

Acknowledgements

We thank Lisa Altvall, Ulrika Hansson, Anna-Lena Magnberg, Vanesa Rufete Bernal, Ute Jesberg, and Johanna Ekberg, who assessed the 222 women in the current study.

Competing Interests

The authors report no conflict of interest.

Funding statement

This study was funded by the Local Research and Development Council Gothenburg and Södra Bohuslän (Grant Number VGFOUGSB-762581) and The Healthcare Board, Region Västra Götaland (Grant Number VGFOUREG-751101).

Availability of data and materials

The dataset from this study is available via a reasonable request to the corresponding author.

Author statement

Each author of this paper meets the criteria for authorship. SV, MFO, AG, GR, and MEHL designed the study. SV collected the data, analyzed, and performed a first interpretation of the results, and drafted the article. MFO, AG, GR, and MEHL participated in the final interpretation of the results, and critically revised the article for important intellectual content. The final manuscript was seen and approved by all authors.

Consent for publication

The individuals shown in Image a+b gave their written informed consent to have these images published in a scientific publication.

For peer review only - http://bmjopen.bmj.com/site/about/guidelines.xhtml

For peer review only

List of Abbreviations

CI: Confidence interval; BMI: Body mass index; ICC: Intraclass correlation coefficient; MDC: Minimal detectable change; PA: Percentage agreement; SD: Standard deviation; SEM: Standard error of measurements

REFERENCES

1. Hodges PW, Sapsford R, Pengel LHM. Postural and respiratory functions of the pelvic floor muscles. *Neurourol. Urodyn.* 2007;26(3):362-371.
2. Smith MD, Coppieters MW, Hodges PW. Postural response of the pelvic floor and abdominal muscles in women with and without incontinence. *Neurourol. Urodyn.* 2007;26(3):377-385.
3. Lien K-C, Mooney B, DeLancey JOL, Ashton-Miller JA. Levator ani muscle stretch induced by simulated vaginal birth. *Obstet. Gynecol.* 2004;103(1):31-40.
4. Staer-Jensen J, Siafarikas F, Hilde G, Benth JS, Bo K, Engh ME. Postpartum recovery of levator hiatus and bladder neck mobility in relation to pregnancy. *Obstet. Gynecol.* 2015;125(3):531-539.
5. South MMT, Stinnett SS, Sanders DB, Weidner AC. Levator ani denervation and reinnervation 6 months after childbirth. *Am. J. Obstet. Gynecol.* 2009;200(5):519.e511-519.e517.
6. Cyr M-P, Kruger J, Wong V, Dumoulin C, Girard I, Morin M. Pelvic floor morphometry and function in women with and without puborectalis avulsion in the early postpartum period. *Am. J. Obstet. Gynecol.* 2017;216(3):274.e271-274.e278.
7. Memon H, Handa VL. Pelvic floor disorders following vaginal or cesarean delivery. *Curr. Opin. Obstet. Gynecol.* 2012;24(5):349-354.
8. Sperstad JB, Tennfjord MK, Hilde G, Ellström-Engh M, Bø K. Diastasis recti abdominis during pregnancy and 12 months after childbirth:

For peer review only

prevalence, risk factors and report of lumbopelvic pain. *British Journal of Sports Medicine.* 2016.

9. Keshwani N, Mathur S, McLean L. Relationship Between Interrectus Distance and Symptom Severity in Women With Diastasis Recti Abdominis in the Early Postpartum Period. *Phys. Ther.* 2018;98(3):182-190.

10. Vieillefosse S, Conté C, Deffieux X. Symptom associated with diastasis recti in the early postpartum period. *European Journal of Obstetrics & Gynecology and Reproductive Biology.* 2019;234:e133-e133.

11. Keeler J, Albrecht M, Eberhardt L, Horn L, Donnelly C, Lowe D. Diastasis Recti Abdominis: A Survey of Women's Health Specialists for Current Physical Therapy Clinical Practice for Postpartum Women. *Journal of Women's Health Physical Therapy.* 2012;36(3):131-142.

12. Bo K, Sherburn M. Evaluation of female pelvic-floor muscle function and strength. *Phys. Ther.* 2005;85(3):269-282.

13. Messelink B, Benson T, Berghmans B, et al. Standardization of terminology of pelvic floor muscle function and dysfunction: report from the pelvic floor clinical assessment group of the International Continence Society. *Neurourol. Urodyn.* 2005;24(4):374-380.

14. Slieker-Ten Hove MCP, Pool-Goudzwaard AL, Eijkemans MJC, Steegers-Theunissen RPM, Burger CW, Vierhout ME. Face validity and reliability of the first digital assessment scheme of pelvic floor muscle function conform the new standardized terminology of the International Continence Society. *Neurourol. Urodyn.* 2009;28(4):295-300.

15. van de Water ATM, Benjamin DR. Measurement methods to assess diastasis of the rectus abdominis muscle (DRAM): A systematic review of their measurement properties and meta-analytic reliability generalisation. *Man. Ther.* 2016;21:41-53.

16. Keeler J, Albrecht M, Eberhardt L, Horn L, Donnelly C, Lowe D. Diastasis Recti Abdominis: A Survey of Women's Health Specialists for

For peer review only - http://bmjopen.bmj.com/site/about/guidelines.xhtml

Current Physical Therapy Clinical Practice for Postpartum Women. *Journal of Women's Health Physical Therapy.* 2012;36(3):131-142.

17. Bursch SG. Interrater reliability of diastasis recti abdominis measurement. *Phys Ther.* 1987;67(7):1077-1079.
18. Barbosa S, de Sa RA, Coca Velarde LG. Diastasis of rectus abdominis in the immediate puerperium: correlation between imaging diagnosis and clinical examination. *Archives of gynecology and obstetrics.* 2013;288(2):299-303.
19. Benjamin DR, Frawley HC, Shields N, Georgiou C, Taylor NF. Establishing measurement properties in the assessment of inter-recti distance of the abdominal muscles in a postnatal women. *Musculoskeletal science & practice.* 2020;49:102202.
20. Lee D, Hodges PW. Behavior of the Linea Alba During a Curl-up Task in Diastasis Rectus Abdominis: An Observational Study. *The Journal of orthopaedic and sports physical therapy.* 2016;46(7):580.
21. Beamish N, Green N, Nieuwold E, McLean L. Differences in Linea Alba Stiffness and Linea Alba Distortion Between Women With and Without Diastasis Recti Abdominis: The Impact of Measurement Site and Task. *The Journal of orthopaedic and sports physical therapy.* 2019;49(9):656-665.
22. Lee DG, Lee LJ, McLaughlin L. Stability, continence and breathing: The role of fascia following pregnancy and delivery. *J. Bodyw. Mov. Ther.* 2008;12(4):333-348.
23. Koo TK, Li MY. A Guideline of Selecting and Reporting Intraclass Correlation Coefficients for Reliability Research. *J. Chiropr. Med.* 2016;15(2):155-163.
24. Landis JR, Koch GG. A One-Way Components of Variance Model for Categorical Data. *Biometrics.* 1977;33(4):671-679.
25. McHugh ML. Interrater reliability: the kappa statistic. *Biochemia medica.* 2012;22(3):276-282.

For peer review only - http://bmjopen.bmj.com/site/about/guidelines.xhtml

26. Bø K, Finckenhagen HB. Vaginal palpation of pelvic floor muscle strength: inter-test reproducibility and comparison between palpation and vaginal squeeze pressure. *Acta Obstet. Gynecol. Scand.* 2001;80(10):883-887.

27. Ferreira CH, Barbosa PB, de Oliveira Souza F, Antonio FI, Franco MM, Bo K. Inter-rater reliability study of the modified Oxford Grading Scale and the Peritron manometer. *Physiotherapy.* 2011;97(2):132-138.

28. Navarro Brazalez B, Torres Lacomba M, de la Villa P, et al. The evaluation of pelvic floor muscle strength in women with pelvic floor dysfunction: A reliability and correlation study. *Neurourol. Urodyn.* 2018;37(1):269-277.

29. Devreese A, Staes F, De Weerdt W, et al. Clinical evaluation of pelvic floor muscle function in continent and incontinent women. *Neurourol. Urodyn.* 2004;23(3):190-197.

30. Bo K, Lilleas F, Talseth T, Hedland H. Dynamic MRI of the pelvic floor muscles in an upright sitting position. *Neurourol. Urodyn.* 2001;20(2):167-174.

31. Fritel X, Ringa V, Quiboeuf E, Fauconnier A. Female urinary incontinence, from pregnancy to menopause: a review of epidemiological and pathophysiological findings. *Acta Obstet. Gynecol. Scand.* 2012;91(8):901-910.

32. Reissing ED, Brown C, Lord MJ, Binik YM, Khalifé S. Pelvic floor muscle functioning in women with vulvar vestibulitis syndrome. *Journal of Psychosomatic Obstetrics & Gynecology.* 2005;26(2):107-113.

33. Mota P, Pascoal AG, Carita AI, Bo K. Normal width of the inter-recti distance in pregnant and postpartum primiparous women. *Musculoskeletal science & practice.* 2018;35:34-37.

34. Mota P, Pascoal AG, Carita AI, Bo K. The Immediate Effects on Inter-rectus Distance of Abdominal Crunch and Drawing-in Exercises During

For peer review only - http://bmjopen.bmj.com/site/about/guidelines.xhtml

Pregnancy and the Postpartum Period. *J. Orthop. Sports Phys. Ther.* 2015;45(10):781-788.

35. Vach W. The dependence of Cohen's kappa on the prevalence does not matter. *J. Clin. Epidemiol.* 2005;58(7):655-661.

36. Dalal K, Kaur A, Mitra M. Correlation between Diastasis Rectus Abdominis and Lumbopelvic Pain and Dysfunction. *Indian Journal of Physiotherapy and Occupational Therapy.* 2014;8(1):210-214.

37. Parker MA, Millar LA, Dugan SA. Diastasis Rectus Abdominis and Lumbo-Pelvic Pain and Dysfunction-Are They Related? *Journal of Women's Health Physical Therapy.* 2009;33(2):15-22.

38. Chiarello CM, McAuley JA. Concurrent Validity of Calipers and Ultrasound Imaging to Measure Interrecti Distance. *J. Orthop. Sports Phys. Ther.* 2013;43(7):495-503.

39. Boxer S, Jones S. Intra-rater reliability of rectus abdominis diastasis measurement using dial calipers. *Aust. J. Physiother.* 1997;43(2):109-114.

For peer review only

Supplement 1: Clinical assessment of pelvic floor and abdominal muscles three months postpartum: An inter-rater reliability study

Clinical assessment protocol: Diastasis recti abdominis

Due to the lack of information about how to apply the jaws of the caliper while measuring the diastasis recti abdominis, we conducted a pilot trial with ten participants before starting the study. The inside and the outside jaws of the caliper were used in random order.

The outside jaws of the caliper are designed to measure the outer boundaries of a firm subject. In the setting of this study, this would be the outer boundaries of the linea alba. The inside jaws of the caliper are designed to measure the inner boundaries of a firm subject, which in the present study would be the inner edges of the rectus abdominis. We found a mean difference of −2.32 mm (SD 2.47) between the inside and the outside jaws. Moreover, measuring with the inside jaws caused more discomfort for the participant, and more insecurity for the investigator regarding the subjective estimation of how much to draw in the inner edge of the rectus abdominis. Thus, it was decided to palpate the outer edges of the linea alba with the fingers, and to measure this distance with the outside jaws (Figure 1).

Figure 1. *Application of the caliper for diastasis recti abdominis measurement*

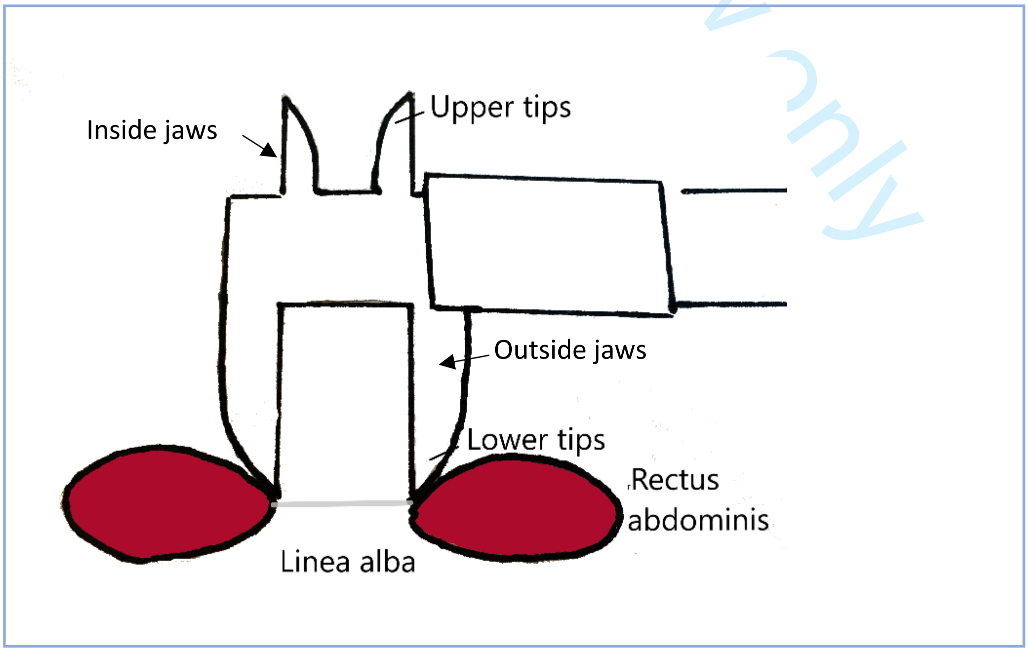

For peer review only

Supplement 1: Clinical assessment of pelvic floor and abdominal muscles three months postpartum: An inter-rater reliability study

Two months after start of the study in September 2018, the assessing physiotherapists expressed a strong uncertainty regarding the right technique for using the caliper. A pre-analysis of the data showed low-to-negative ICC values and large differences between the measurements. Thus, we conducted an additional training for all centers. The six physiotherapists underwent a calibration process in form of after each other assessing ten person`s abdominal muscles, comparing their results and discussing their technique. At this point in time, rehabilitation center 3 had not yet started their assessments. In the additional training, the assessing physiotherapists identified three important confounders while measuring:

1. Accurate lift of the head (2–3 cm) was an important factor for correct measurement. With less lift, there was no contraction in the abdominal muscles and it was almost impossible to find the inner edges of the *musculus rectus abdominis* and with a higher lift the distance between the two bellies of the *musculus recti abdominis* was decreasing which is in line with the study of Mota et al showing that an abdominal crunch is narrowing the inter-recti distance.[1]
2. Pre-activation in the deeper abdominal muscles impeded the measurement. Due to this the participants were encouraged to relax before lifting the head.
3. It was easier to palpate the junction between the rectus abdominis and linea alba during the eccentric movement of lowering the head.

<u>After the additional training, the assessment protocol was set as follows:</u>

The diastasis recti abdominis was assessed with the participant in the supine position on a flat bench, with the legs flexed, and their arms held at their side. The assessing physiotherapist used a water-soluble marker to mark the three measurement points: at the umbilicus, and at 4.5 cm above and 4.5 cm below the umbilicus.[2,3] For accurate assessment of the activated

For peer review only - http://bmjopen.bmj.com/site/about/guidelines.xhtml

Supplement 1: Clinical assessment of pelvic floor and abdominal muscles three months postpartum: An inter-rater reliability study

abdominal muscles, the participant had to lift her head 2–3 cm from the bench. Before the assessment began, the physiotherapist assured that the participant correctly lifted her head 2–3 cm, which was trained by several repetitions.

- **Diastasis recti abdominis width:** The participant was asked to lift her head and then slowly lower it. As seen in other studies[4,5], this concentric and eccentric movement is helping the physiotherapist to palpate the outer edges of the linea alba with their index and middle finger, without examination gloves. To avoid pre-activation of the deeper abdominal muscle, the participants were asked to relax before performing the trained head lift of 2–3 cm. During this movement -resulting in an activation of the musculus rectus abdominis- the physiotherapist identified the distance between the two parts of the rectus abdominis with her fingers and measured this felt distance using the caliper (Image 1a). The same procedure was conducted at all three measurement points.

Image 1. a) *Measuring the diastasis recti abdominis width with the caliper.* ***b)*** *Observation of diastasis recti abdominis bulging*

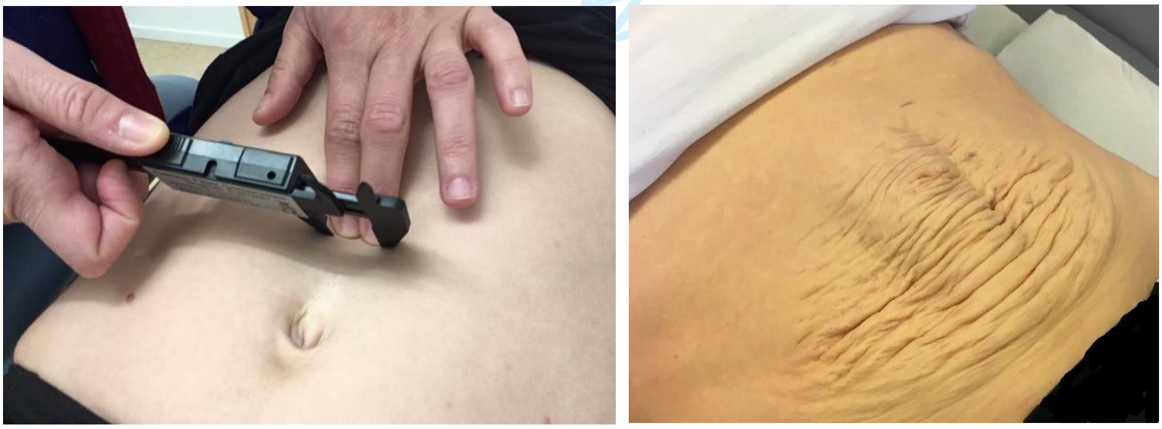

- **Diastasis recti abdominis depth:** The participant was asked to repeat the exact same head lift of 2–3 cm. At all three measurement points, the physiotherapist palpated the tension in the linea alba (without adding pressure), and rated it as “good resistance at

For peer review

For peer review only - http://bmjopen.bmj.com/site/about/guidelines.xhtml

all points", "resistance in the depth at measurement point x", or "bottomless resistance at measurement point x".

- **Diastasis recti abdominis bulging:** The participant performed a 3-step sit-up test.[6] The participant was laying supine with straight legs on the flat bench. The test was rated as 0 if the participant was not able to perform a sit-up, as 1 if the participant was able to raise the upper torso to 40-degree angle from the bench with straight and secured legs, arms at the side of the body. The test was rated as 2 if the participant was able to perform the same task with the hands held behind the head and as 3 if the participant was able to perform a sit-up with the hands behind her head and hips and knees flexed and not secured. The participant had to hold the sit-up position for 5 seconds During this test, the physiotherapist observed whether the linea alba bulged during the movement (Image 1b).

References:

1. Mota P, Pascoal AG, Carita AI, Bo K. The Immediate Effects on Inter-rectus Distance of Abdominal Crunch and Drawing-in Exercises During Pregnancy and the Postpartum Period. J. Orthop. Sports Phys. Ther. 2015;45(10):781-788.
2. Chiarello CM, Falzone LA, McCaslin KE, Patel MN, Ulery KR. The effects of an exercise program on diastasis recti abdominis in pregnant women. Journal of Women's Health Physical Therapy. 2005;29(1):11-16.
3. Chiarello CM, McAuley JA. Concurrent validity of calipers and ultrasound imaging to measure interrecti distance. The Journal of orthopaedic and sports physical therapy. 2013;43(7):495-503.
4. Parker MA, Millar LA, Dugan SA. Diastasis Rectus Abdominis and Lumbo-Pelvic Pain and Dysfunction-Are They Related? Journal of Women's Health Physical Therapy. 2009;33(2):15-22.
5. Dalal K, Kaur A, Mitra M. Correlation between Diastasis Rectus Abdominis and Lumbopelvic Pain and Dysfunction. Indian Journal of Physiotherapy and Occupational Therapy. 2014;8(1):210-214.
6. Hills NF, Graham RB, McLean L. Comparison of Trunk Muscle Function Between Women With and Without Diastasis Recti Abdominis at 1 Year Postpartum. Phys. Ther. 2018;98(10):891.

For peer review only - http://bmjopen.bmj.com/site/about/guidelines.xhtml

Supplement 2: Clinical assessment of pelvic floor and abdominal muscles three months postpartum: An inter-rater reliability study

Clinical assessment protocol: Pelvic floor muscles

The pelvic floor muscles were assessed with the participant in the supine position, with the legs flexed and slightly abducted on a plinth, and a pillow under the head. Participants were assessed by observation and digital palpation. During observation of pelvic floor muscle contraction, the physiotherapist stood beside the bench, supporting the participant's legs with her hands and observing the movement of the perineum:

- **Involuntary contraction**: The participant was asked to cough forcefully; the movement of the perineum were rated as downwards/ perineal in-drawing/ no movement
- **Voluntary contraction**, the participant was given the verbal cue "contract your pelvic floor muscles like you want to prevent the escape of gas/urine". The movement of the perineum was rated as downwards/ perineal in-drawing/ no movement

Vaginal palpation of the pelvic floor muscles was performed by physiotherapists using examinations gloves and water-based lubricant. The index and middle finger were inserted 2–3 cm into the vagina with the palmar side directed to the caudal part of the vagina, palpating the pubovisceral portion of the levator ani.[1]

- **Involuntary contraction**: The participant was asked to forcefully cough three times. The absence or presence of a correct contraction, defined as a squeeze around the pelvic openings and an inward lift,[2] were rated.
- **Maximal voluntary contraction,** the participant was asked to contract the pelvic floor muscles. If the physiotherapist felt a correct contraction, the participant was encouraged to activate their pelvic floor muscles "as strong and as long you can". Of three maximal voluntary contractions, the strongest was rated on a 6-point

For peer review only

modified Oxford Scale. The scale was modified for the study with the aim to assess both the lifting and the squeezing component of the pelvic floor muscle function. The onset of the lifting component in the contraction were set as a criterion for being rated ≥3 on the scale (Figure 2).

***Figure 1**. The modified Oxford Scale used in this study*

**0**	No contraction
**1**	Partial contraction, "flicker"
**2**	Weak contraction, no correct lift
**3**	Good contraction, definite lift
**4**	Maximum contraction with lift, not able to hold longer than 5 seconds
**5**	Maximum contraction with lift, able to hold ___ seconds

The participants rested 15 seconds between the contractions. If a participant, despite several attempts and verbal cues, failed to squeeze and lift and was instead straining, the participant´s results were excluded from the statistical analysis of maximal voluntary contraction and pelvic floor muscle endurance.

- **Pelvic floor muscle endurance**, after 15 seconds of rest, the participant was asked to contract the pelvic floor muscles for as long as possible at approximately 50% of the previous contraction strength. Pelvic floor muscle endurance was rated as positive if the participant was able to hold this contraction for longer than 30 seconds.

For peer review only

Supplement 2: Clinical assessment of pelvic floor and abdominal muscles three months postpartum: An inter-rater reliability study

- **Voluntary relaxation**, the participant was given the verbal cue "try to relax your pelvic floor muscles, let the vagina get larger and go downwards". This function was rated as absent, partial, or complete.

References:

1. Kearney R, Miller JM, DeLancey JOL. Interrater reliability and physical examination of the pubovisceral portion of the levator ani muscle, validity comparisons using MR imaging. Neurourol. Urodyn. 2006;25(1):50-54.
2. Bo K, Sherburn M. Evaluation of female pelvic-floor muscle function and strength. Phys. Ther. 2005;85(3):269-282.

For peer review only

Section & Topic	No	Item	Reported on page #
**TITLE OR ABSTRACT**			
	**1**	Identification as a study of diagnostic accuracy using at least one measure of accuracy (such as sensitivity, specificity, predictive values, or AUC)	1
**ABSTRACT**			2
	**2**	Structured summary of study design, methods, results, and conclusions (for specific guidance, see STARD for Abstracts)	
**INTRODUCTION**			
	**3**	Scientific and clinical background, including the intended use and clinical role of the index test	3-5
	**4**	Study objectives and hypotheses	5
**METHODS**			
Study design	**5**	Whether data collection was planned before the index test and reference standard were performed (prospective study) or after (retrospective study)	5
Participants	**6**	Eligibility criteria	6
	**7**	On what basis potentially eligible participants were identified (such as symptoms, results from previous tests, inclusion in registry)	6
	**8**	Where and when potentially eligible participants were identified (setting, location and dates)	6
	**9**	Whether participants formed a consecutive, random or convenience series	6
Test methods	**10a**	Index test, in sufficient detail to allow replication	7, supplement 1+2
	**10b**	Reference standard, in sufficient detail to allow replication	N/A
	**11**	Rationale for choosing the reference standard (if alternatives exist)	N/A
	**12a**	Definition of and rationale for test positivity cut-offs or result categories of the index test, distinguishing pre-specified from exploratory	N/A
	**12b**	Definition of and rationale for test positivity cut-offs or result categories of the reference standard, distinguishing pre-specified from exploratory	N/A
	**13a**	Whether clinical information and reference standard results were available to the performers/readers of the index test	17
	**13b**	Whether clinical information and index test results were available to the assessors of the reference standard	N/A
Analysis	**14**	Methods for estimating or comparing measures of diagnostic accuracy	8-9
	**15**	How indeterminate index test or reference standard results were handled	8-9
	**16**	How missing data on the index test and reference standard were handled	N/A
	**17**	Any analyses of variability in diagnostic accuracy, distinguishing pre-specified from exploratory	8-9
	**18**	Intended sample size and how it was determined	5
**RESULTS**			
Participants	**19**	Flow of participants, using a diagram	N/A
	**20**	Baseline demographic and clinical characteristics of participants	10
	**21a**	Distribution of severity of disease in those with the target condition	N/A
	**21b**	Distribution of alternative diagnoses in those without the target condition	10
	**22**	Time interval and any clinical interventions between index test and reference standard	N/A
Test results	**23**	Cross tabulation of the index test results (or their distribution) by the results of the reference standard	N/A
	**24**	Estimates of diagnostic accuracy and their precision (such as 95% confidence intervals)	11, 14-15
	**25**	Any adverse events from performing the index test or the reference standard	10
**DISCUSSION**			
	**26**	Study limitations, including sources of potential bias, statistical uncertainty, and generalisability	16, 18-19
	**27**	Implications for practice, including the intended use and clinical role of the index test	20
**OTHER INFORMATION**			
	**28**	Registration number and name of registry	5-6
	**29**	Where the full study protocol can be accessed	21
	**30**	Sources of funding and other support; role of funders	21

For peer review only - http://bmjopen.bmj.com/site/about/guidelines.xhtml

STARD 2015

AIM

STARD stands for “Standards for Reporting Diagnostic accuracy studies”. This list of items was developed to contribute to the completeness and transparency of reporting of diagnostic accuracy studies. Authors can use the list to write informative study reports. Editors and peer-reviewers can use it to evaluate whether the information has been included in manuscripts submitted for publication.

EXPLANATION

A **diagnostic accuracy study** evaluates the ability of one or more medical tests to correctly classify study participants as having a **target condition.** This can be a disease, a disease stage, response or benefit from therapy, or an event or condition in the future. A medical test can be an imaging procedure, a laboratory test, elements from history and physical examination, a combination of these, or any other method for collecting information about the current health status of a patient.

The test whose accuracy is evaluated is called **index test.** A study can evaluate the accuracy of one or more index tests. Evaluating the ability of a medical test to correctly classify patients is typically done by comparing the distribution of the index test results with those of the **reference standard**. The reference standard is the best available method for establishing the presence or absence of the target condition. An accuracy study can rely on one or more reference standards.

If test results are categorized as either positive or negative, the cross tabulation of the index test results against those of the reference standard can be used to estimate the **sensitivity** of the index test (the proportion of participants *with* the target condition who have a positive index test), and its **specificity** (the proportion *without* the target condition who have a negative index test). From this cross tabulation (sometimes referred to as the contingency or “2x2” table), several other accuracy statistics can be estimated, such as the positive and negative **predictive values** of the test. Confidence intervals around estimates of accuracy can then be calculated to quantify the statistical **precision** of the measurements.

If the index test results can take more than two values, categorization of test results as positive or negative requires a **test positivity cut-off**. When multiple such cut-offs can be defined, authors can report a receiver operating characteristic (ROC) curve which graphically represents the combination of sensitivity and specificity for each possible test positivity cut-off. The **area under the ROC curve** informs in a single numerical value about the overall diagnostic accuracy of the index test.

The **intended use** of a medical test can be diagnosis, screening, staging, monitoring, surveillance, prediction or prognosis. The **clinical role** of a test explains its position relative to existing tests in the clinical pathway. A replacement test, for example, replaces an existing test. A triage test is used before an existing test; an add-on test is used after an existing test.

Besides diagnostic accuracy, several other outcomes and statistics may be relevant in the evaluation of medical tests. Medical tests can also be used to classify patients for purposes other than diagnosis, such as staging or prognosis. The STARD list was not explicitly developed for these other outcomes, statistics, and study types, although most STARD items would still apply.

DEVELOPMENT

This STARD list was released in 2015. The 30 items were identified by an international expert group of methodologists, researchers, and editors. The guiding principle in the development of STARD was to select items that, when reported, would help readers to judge the potential for bias in the study, to appraise the applicability of the study findings and the validity of conclusions and recommendations. The list represents an update of the first version, which was published in 2003.

More information can be found on http://www.equator-network.org/reporting-guidelines/stard.

cc BY SA

For peer review only - http://bmjopen.bmj.com/site/about/guidelines.xhtml

BMJ Open

Clinical assessment of pelvic floor and abdominal muscles three months postpartum: An inter-rater reliability study

Journal:	*BMJ Open*
Manuscript ID	bmjopen-2021-049082.R2
Article Type:	Original research
Date Submitted by the Author:	27-Jul-2021
Complete List of Authors:	Vesting, Sabine; Göteborgs universitet Institutionen för neurovetenskap och fysiologi, Department of Health and Rehabilitation<br>Olsen, Monika Fagevik; Göteborgs universitet Institutionen för neurovetenskap och fysiologi, Department of Health and Rehabilitation<br>Gutke, Annelie; Göteborgs universitet Institutionen för neurovetenskap och fysiologi, Department of Health and Rehabilitation<br>Rembeck, Gun; University of Gothenburg School of Public Health and Community Medicine<br>Larsson, Maria; Göteborgs universitet Institutionen för neurovetenskap och fysiologi, Department of Health and Rehabilitation
**Primary Subject Heading**:	Obstetrics and gynaecology
Secondary Subject Heading:	Rehabilitation medicine
Keywords:	Maternal medicine < OBSTETRICS, Urinary incontinences < UROLOGY, REHABILITATION MEDICINE, Urogynaecology < GYNAECOLOGY

SCHOLARONE™ Manuscripts

I, the Submitting Author has the right to grant and does grant on behalf of all authors of the Work (as defined in the below author licence), an exclusive licence and/or a non-exclusive licence for contributions from authors who are: i) UK Crown employees; ii) where BMJ has agreed a CC-BY licence shall apply, and/or iii) in accordance with the terms applicable for US Federal Government officers or employees acting as part of their official duties; on a worldwide, perpetual, irrevocable, royalty-free basis to BMJ Publishing Group Ltd ("BMJ") its licensees and where the relevant Journal is co-owned by BMJ to the co-owners of the Journal, to publish the Work in this journal and any other BMJ products and to exploit all rights, as set out in our licence.

The Submitting Author accepts and understands that any supply made under these terms is made by BMJ to the Submitting Author unless you are acting as an employee on behalf of your employer or a postgraduate student of an affiliated institution which is paying any applicable article publishing charge ("APC") for Open Access articles. Where the Submitting Author wishes to make the Work available on an Open Access basis (and intends to pay the relevant APC), the terms of reuse of such Open Access shall be governed by a Creative Commons licence – details of these licences and which Creative Commons licence will apply to this Work are set out in our licence referred to above.

Other than as permitted in any relevant BMJ Author's Self Archiving Policies, I confirm this Work has not been accepted for publication elsewhere, is not being considered for publication elsewhere and does not duplicate material already published. I confirm all authors consent to publication of this Work and authorise the granting of this licence.

For peer review only

Clinical assessment of pelvic floor and abdominal muscles three months postpartum: An inter-rater reliability study

Sabine Vesting, MSc [1, 2] 0000-0002-6523-1833

Monika Fagevik Olsén, PhD [1, 3+4] 0000-0002-0207-4105

Annelie Gutke, PhD [1] 0000-0003-3484-9769

Gun Rembeck, PhD [5-7] 0000-0003-0097-5150

Maria E.H. Larsson, PhD [1, 8] 0000-0002-1225-9736

Author information:

1 Department of Health and Rehabilitation, Unit of Physiotherapy, Institute of Neuroscience and Physiology, Sahlgrenska Academy, University of Gothenburg, Gothenburg, Sweden.

2 Närhälsan Gibraltar Rehabilitation, Gothenburg, Sweden.

3 Department of Physical Therapy and Occupational Therapy, Sahlgrenska University Hospital, Sweden

4 Department of Gastrosurgical Research & Education, Sahlgrenska Academy, Gothenburg University, Gothenburg, Sweden.

5 Region Västra Götaland, Research and Development Centre Södra Älvsborg, Borås, Sweden

6 Region Västra Götaland, Regionhälsan Borås Youth Centre, Borås, Sweden

7 Primary Health Care, School of Public Health and Community Medicine, Institute of Medicine, Sahlgrenska Academy, University of Gothenburg, Gothenburg, Sweden.

8 Region Västra Götaland Education, Research and Development Primary Health Care, Gothenburg, Sweden.

Corresponding author: Sabine Vesting, MSc., University of Gothenburg, Box 430, 405 30 Gothenburg, Sweden, mobile phone 0046-722095484, sabine.vesting@gu.se

Word count: Main document (4580), Abstract (299), Tables (5)

For peer review only - http://bmjopen.bmj.com/site/about/guidelines.xhtml

ABSTRACT

Objectives: Evaluation of the inter-rater reliability of clinical assessment methods for pelvic floor muscles and diastasis recti abdominis postpartum.

Design: A multi-center inter-rater reliability study.

Setting: Three primary care rehabilitation centers in Sweden.

Participants: A total of 222 participants were recruited via advertising at Swedish maternity care units and social media. Eligibility for participation included female gender, ≥18 years, at maximum three months after childbirth. Exclusion criteria were chronic pelvic girdle pain and/or low back pain and/or pelvic floor tear grade III/IV. At each center, 2 physiotherapists, with training and experience in pelvic floor assessment, assessed the 222 women according to a standardized protocol in random order.

Outcome measures: Inter-rater reliability of the assessment of pelvic floor muscle function (involuntary and voluntary contraction and voluntary relaxation) and diastasis recti abdominis (width, depth, and bulging).

Results: Vaginal palpation of maximal voluntary contraction revealed a kappa value of 0.69 [95% CI 0.62; 0.76]. Assessments of involuntary contraction, and voluntary relaxation yielded inconsistent results, with slight-to-moderate weighted kappa values ranging from 0.10–0.51. After two months of training in applying this method, diastasis recti abdominis width measured at the umbilicus by caliper yielded an ICC value of 0.83 [95% CI 0.76; 0.87]. Assessments of diastasis recti abdominis depth and bulging showed moderate kappa values, with reservation for some inconsistency between the centers.

Conclusions: Vaginal palpation of pelvic floor muscle strength is a reliable method for the postpartum muscle assessment. Additional research is needed to identify reliable assessment method for other pelvic floor muscle functions like involuntary contraction and voluntary

For peer review only - http://bmjopen.bmj.com/site/about/guidelines.xhtml

relaxation. With some training, a caliper is a reliable instrument for measuring the postpartum diastasis recti abdominis width. This study provides novel thoughts about how to measure diastasis recti abdominis depth and bulging.

Trial registration: Registered at clinicaltrial.gov, October 2018, ClinicalTrials.gov Identifier NCT03703804.

Keywords: childbirth, caliper, diastasis recti abdominis, inter-rater, palpation, pelvic floor muscles

For peer

STRENGTHS AND LIMITATIONS

1. This study provides a detailed description of clinically applicable assessment methods for postpartum muscle assessment in primary care.
2. This study is raising novel thoughts about the question how diastasis recti abdominis depth and bulging could be assessed postpartum.
3. The need of training and experience in the clinical assessment of the diastasis recti abdominis was underestimated before start of the study.
4. The used assessment methods were not validated for example by comparing the results with technical devices like ultrasound and/or pressure measurement.
5. The assessment methods were tested at three primary care rehabilitation centers, adding training and experience and maybe even continuity as important aspects for a reliable assessment

INTRODUCTION

Both the pelvic floor and the abdominal muscles are part of the stabilization system for the pelvic and spine[1] as well as the continence system.[2] Pregnancy and childbirth cause alterations in these muscle groups. During a vaginal delivery, the pelvic floor muscles overstretches up to

For peer review only - http://bmjopen.bmj.com/site/about/guidelines.xhtml

three times.[3] It takes approximately six months until muscles, nerves and the connective tissue are recovered from a vaginal delivery.[4,5] Childbirth is associated with pelvic floor traumas, such as perineal tears and levator ani injuries, which can lead to incontinence, pelvic organ prolapse, and decreased quality of life.[6,7] Another postpartum muscle alteration is a persistent separation of the two parts of the rectus abdominis, termed a diastasis recti abdominis. At 12 months postpartum, 33% of women exhibit a diastasis recti abdominis greater than the width of two fingers.[8] A diastasis recti abdominis is reportedly correlated with impaired quality of life, negative body image, and abdominal pain.[9,10]

Women are increasingly seeking help and advice regarding postpartum muscle alterations from physiotherapists at primary health care centers. Physiotherapists use various methods to assess the pelvic floor muscles and diastasis recti abdominis after pregnancy,[11,12] however, there is currently no gold standard. Clinically, vaginal palpation and observation are common methods to assess different pelvic floor muscle functions after childbirth. The International Continence Society defined that the pelvic floor muscle can be involuntarily and voluntarily contracted and can also be voluntarily relaxed.[13] However, there are no standardized assessment protocols or rating scales in the postpartum care, yet. One study reported the use of a Delphi scheme to identify the optimal protocol for assessing different pelvic floor muscle functions and tested their inter-rater reliability,[14] but these assessments were not performed in postpartum women.

Ultrasound assessment is the most reliable and valid method for measurement of the diastasis recti abdominis width.[15] However, most women who are concerned about their diastasis recti abdominis seek help at primary healthcare centers, where ultrasound is seldom available. About 96% of American physiotherapists specialized in women's health assess the diastasis recti abdominis width using the finger-width method,[8,16] which is imprecise due to finger-width variations[17] and has weaker inter-rater reliability than instrumental assessment

For peer review only - http://bmjopen.bmj.com/site/about/guidelines.xhtml

methods.[15] Less than 2% of American physiotherapists use calipers for the assessment of postpartum women.[16] Diastasis recti abdominis width assessment using a caliper is reported to be nearly as accurate as ultrasound assessment,[15,18] although the inter-rater reliability of this method has been tested in just one study.[19]

An experimental study suggests that the tendon between the two parts of the rectus abdominis—the linea alba— has less tension in a curl-up movement in women with diastasis recti abdominis.[20] These findings of Lee and Hodges were strengthened by a further study measuring the tension and stiffness in the linea alba.[21] Both research groups argue that the tension in the linea alba is an important factor for the load transfer in the core and might be one of the key components to explain why women with diastasis recti abdominis experience functional impairments. However, these studies were experimental, and their hypothesis is not validated yet. The assessments in these studies were performed by ultrasound [20] and shear-wave elastography.[21] For clinical practice, there are currently no reliable and clinically applicable assessment methods or rating scales for linea alba tension or stiffness. Hypothetical, the tension in the linea alba could be palpated, the inability to control intraabdominal pressure could be observed as diastasis recti abdominis bulging.[22]

In the present study, we aimed to evaluate the inter-rater reliability of different aspects of the clinical assessment of pelvic floor muscle and diastasis recti abdominis using observation, calipers, and palpation at three months postpartum.

MATERIAL AND METHODS

Study design and subjects

In total, 222 women from the Region Västra Götaland, Sweden were included to this reliability study. Based on the guidelines of Ko & Li, we aimed to assess at least 30 participants at each center.[23] Assessments were conducted at three primary care rehabilitation centers. The study

For peer review only - http://bmjopen.bmj.com/site/about/guidelines.xhtml

was registered at clinicaltrial.gov, October 2018, with the Identifier NCT03703804 and at the Research and Development Centre Gothenburg and Södra Bohuslän with the registration number 243071. All included women gave their written informed consent to participate. The study protocol was approved by the Swedish ethical review authority in Gothenburg in April 2018 (Dnr 088-18) and registered with the local data protection service, Närhälsan Gothenburg, in January 2019. This study is in line with the Helsinki declaration and the STARD (Standards for Reporting Diagnostic accuracy studies) checklist was used to report this study about assessment methods.

The women were invited to participate via advertising at antenatal and childcare centers, and social media. Inclusion criteria were age of ≥18 years, vaginal delivery or caesarean section within the past three months, and the ability to understand and respond in Swedish. Exclusion criteria were chronic pelvic girdle pain and/or low back pain (defined as self-reported pelvic or low back pain for over three months, not related to the last pregnancy) and/or pelvic floor tear grade III/IV.

The participants were contacted and booked for assessment at one of the three primary care rehabilitation centers in the Västra Götaland region between eight to twelve weeks after giving birth. Assessments were performed by six physiotherapists—two at each primary care rehabilitation center. Prior to the assessments, the participants completed a questionnaire about their age, body mass index (BMI), mode of delivery, number of delivered children, self-reported pelvic floor tears, most recent baby's birth weight, and the birth weights of previous children (if applicable).

The assessing physiotherapists had each completed a four-day (or longer) course in pelvic floor muscle assessment and treatment methods. The physiotherapists at primary care rehabilitation center 1 had two and nine years' experience in pelvic floor muscle assessment at start of the study, the physiotherapists at primary care rehabilitation center 2 had one and three

For peer review only - http://bmjopen.bmj.com/site/about/guidelines.xhtml

years' experience and the physiotherapists at primary care rehabilitation center 3 had both one year experience in pelvic floor muscle assessment at start of the study. All six physiotherapists work at primary care rehabilitation centers, part- of fulltime with women´s health. They had experience in palpating the diastasis recti abdominis with the finger-width method. However, similar to the low numbers of American physiotherapists using the caliper,[11] caliper measurement was new to all rates as it is unusual in Sweden and diastasis recti abdominis assessment is not part of the Swedish physiotherapy education. Therefore, all included physiotherapists were novices at measuring the diastasis recti abdominis by caliper, and on using the rating scales for depth and bulging. During the design phase of this study, four hours of training in diastasis recti abdominis measurement was planned.

Due to an insecurity among the physiotherapists in assessing and measuring the diastasis recti abdominis in the beginning of the study, the first two months of the study (63 measurements) were used for training and validation of the right technique for using the caliper and assessing depth and bulging. After these two months, the physiotherapists at all centers underwent additional training, comparing their results and discussing their technique. The whole validation process and the final assessment protocol after validation is available in Supplement 1.

Patient and public involvement

Patients and the public were not actively involved in the initial design of the study. However, the application of the caliper was tested in a pilot trial and the opinions of the participants about the application were considered for the design of the final assessment protocol (Supplement 1).

Clinical assessment of the pelvic floor muscles

The pelvic floor muscles were assessed with the participant in supine position on a flat bench, with the legs flexed and slightly abducted. The participants had a pillow under the head. The

For peer review only - http://bmjopen.bmj.com/site/about/guidelines.xhtml

assessed pelvic floor muscle functions were involuntary and voluntary contraction (by observation and vaginal palpation) and maximal voluntary contraction, pelvic floor muscle endurance and voluntary relaxation (by vaginal palpation). The detailed assessment protocol is shown in Supplement 2.

Clinical assessment of the diastasis recti abdominis

The assessment of the diastasis recti abdominis was conducted in supine position on a flat bench, without a pillow. The participants were in hook-lying position with their arms resting at their sides. The physiotherapists assessed diastasis recti abdominis width using an electronic digital caliper (150 mm, carbon fiber, accuracy ±0.2 mm, 24 se Sverige AB, Kalmar, Sweden). Diastasis recti abdominis depth and bulging were assessed by observation and palpation. All final measures or ratings were results of single assessments to avoid a fatigue effect. The step-wise explanation of caliper application and assessment of diastasis recti abdominis is explained in Supplement 1.

Upon completion of the assessment, the participants rested for 30 minutes in a sitting or lying position. After the 30-minute rest, the second physiotherapist conducted the same assessment as described above. The two physiotherapists at each site were assessing in random order, were blinded to each other's findings, and not allowed to talk about their assessments. The physiotherapist were also blinded to the participants background information.

Statistical analysis

Statistical analyses were performed using IBM SPSS statistical package version 25 (SPSS Inc., Chicago, IL). Descriptive statistics are presented as mean and standard deviation (SD)for ratio data, and as number and percentage for nominal and ordinal data. To calculate statistically significant differences between the three rehabilitation centers, we used the one-way ANOVA

For peer review only - http://bmjopen.bmj.com/site/about/guidelines.xhtml

test for ratio data, and the Kruskal-Wallis test for ordinal data and chi-squared test for categorical data. A p value of ≤0.05 was regarded as statistically significant.

All pelvic floor muscles functional measures were rated on ordinal scales, except for pelvic floor muscle endurance and involuntary contraction by palpation. Diastasis recti abdominis depth was also rated on an ordinal scale. Ratings on ordinal scales were evaluated by Cohen's weighted kappa values. Pelvic floor muscle endurance, involuntary contraction by palpation, and diastasis recti abdominis bulging were rated on nominal scales, and these ratings were evaluated by Cohen's kappa values. For interpretation of kappa values, we used the categories of Landis and Koch: <0.2, slight; 0.21–0.40, fair; 0.41–0.60, moderate; 0.61–0.80, substantial; and 0.81–1.0, almost perfect agreement.[24] Percentage agreement was calculated and presented for all nominal and ordinal data, and <60% agreement was defined as faulty agreement.[25]

For assessment of diastasis recti abdominis width, a continuous scale (in mm) was used. To evaluate the inter-rater reliability of the assessments on a continuous scale, we calculated the intraclass correlation coefficient (ICC) and 95% confidence interval (CI). ICC values were calculated in SPSS based on absolute agreement and a 2-way mixed effects model. ICC values of <0.50 indicate poor reliability, 0.50–0.75 indicate moderate reliability, 0.75–0.90 good reliability, and values of >0.90 indicate excellent reliability [23].

To further evaluate reliability, we calculated the standard error of measurements ($SEM = SD \times \sqrt{(1 - ICC)}$), which represent the typical error in a single measurement and the minimal detectable change ($MDC = SEM \times 1.96 \times \sqrt{2}$). For calculation of the standard error of measurements (SEM), we used the standard deviation (SD) from the scores of all subjects. SEM and MDC values are presented in mm.

For peer review only - http://bmjopen.bmj.com/site/about/guidelines.xhtml

RESULTS

A total of 222 women were assessed, with measurements conducted from September 2018 through February 2020. The mean age of the participants was 33.1 years (SD ±3.3) and the majority of women (61%) had delivered one child (Table 1). The participating women at primary care rehabilitation center 3 were significantly younger than the women at primary care rehabilitation center 2 and had significantly more children than the participants at primary care rehabilitation centers 1 and 2.

For peer review only

For peer review only - http://bmjopen.bmj.com/site/about/guidelines.xhtml

Table 1. Characteristics of the participating women at three months postpartum (N=222)

	Total (n = 222)	Primary care rehabilitation center 1 (n = 90)	Primary care rehabilitation center 2 (n = 103)	Primary care rehabilitation center 3 (n = 29)	*p* [a]
	Mean ±SD	*Mean* ±SD	*Mean* ±SD	*Mean* ±SD	
**Age in years**	33.1 ±3.3	32.6 ±3.5	33.8 ±2.9	32.0 ±3.8	**0.01**
**BMI**	24.5 ±3.0	24.4 ±3.1	24.3 ±2.9	25.1 ±3.2	0.42
**Neonatal birth weight**	3574.5 ±507.0	3517.7 ±533.7	3604.7 ±489.2	3641.1 ±484.0	0.59
	n (%)	n (%)	n (%)	n (%)	
**Delivery mode**					0.48
C-section	28 (13)	9 (10)	16 (16)	3 (10)	
Vaginal delivery	194 (87)	81 (90)	87(85)	26 (90)	
**Number of children**					**<0.01**
1	137 (61)	51 (57)	75 (73)	11 (30)	
2	74 (33)	33 (37)	26 (25)	15 (50)	
3	9 (4)	7 (6)	1 (1)	1 (3)	
4 or more	3 (1)	0 (0)	1 (1)	2 (7)	
**Self-reported perineal tear**					0.33
No tear	54 (24)	24 (27)	23 (22)	7 (24)	
First-degree perineal tear	52 (23	16 (18)	24 (23)	12 (41)	
Second-degree perineal tear /episiotomy	74 (33)	33 (38)	35 (34)	6 (20)	

n, number; BMI, body mass index; SD, standard deviation.

a) *p* value by one-way ANOVA test for ratio data, Kruskal-Wallis test for ordinal data and chi-squared test for categorical data.

Clinical assessment of the pelvic floor muscles

The evaluation of maximal voluntary contraction showed substantial agreement (weighted kappa value, 0.69 [95 % CI 0.62 to 0.76]), and assessment of pelvic floor muscle endurance showed moderate agreement (kappa value, 0.49 [95% CI 0.37 to 0.61]) (Table 2). Seven participants (3.3%) were excluded from the analyses of maximal voluntary contraction and

For peer review only - http://bmjopen.bmj.com/site/about/guidelines.xhtml

pelvic floor muscle endurance due to incorrect pelvic floor muscle contraction (straining). Assessment of voluntary contraction by observation showed moderate agreement, with a weighted kappa of 0.45 [95% CI 0.28 to 0.62]. Among all assessments, about 89% were rated as “perineal inward movement”.

Table 2. *Results of the clinical assessment of pelvic floor muscles via observation and vaginal palpation*

For peer review only

Parameters	Total group (n = 222)		Primary care rehabilitation center 1 (n = 90)		Primary care rehabilitation center 2 (n = 103)		Primary care rehabilitation center 3 (n = 29)	
	Kappa [95% CI]	PA %	Kappa [95% CI]	PA %	Kappa [95% CI]	PA %	Kappa [95% CI]	PA %
**Voluntary contraction**								
**Observation**	0.45 [0.28; 0.62]	90	0.40 [0.11; 0.70]	91	0.55 [0.33; 0.77]	90	0.29 [−0.13; 0.70]	86
**Vaginal palpation**								
**MVC[b]**	0.69 [0.62; 0.76]	71	0.70 [0.58; 0.82]	77	0.59 [0.46; 0.71]	67	0.67 [0.50; 0.84]	62
**PFM Endurance[b]**	0.49 [0.37; 0.61]	74	0.68 [0.53; 0.83]	84	0.27 [0.09; 0.45]	65	0.55 [0.20; 0.90]	83
**Involuntary contraction**								
**Observation**	0.10 [−0.02; 0.22]	57	0.20 [−0.01; 0.42]	77	0.11 [−0.05; 0.27]	48	0.38 [0.10; 0.67]	68
**Vaginal palpation**	0.51 [0.37; 0.65]	85	0.26 [-0.04; 0.56]	87	0.47 [0.25; 0.69]	85	0.47 [0.14; 0.80]	75
**Voluntary relaxation**								
**Vaginal palpation**	0.26 [0.15; 0.37]	57	0.30 [0.16; 0.50]	63	−0.08 [−0.23; 0.07]	45	0.56 [0.07; 1.02]	89

n, number; CI, confidence interval; PA, percentage agreement; MVC, maximal voluntary contraction; PFM, pelvic floor muscle.

b) reduced number of participants due to incorrect pelvic floor muscle contraction (straining): total group (*n* = 215), primary care rehabilitation center 1 (*n* = 88), primary care rehabilitation center 2 (*n* = 98), primary care rehabilitation center 3 (*n* = 29).

For peer review only - http://bmjopen.bmj.com/site/about/guidelines.xhtml

The assessment of involuntary contraction by observation exhibited slight agreement (weighted kappa value, 0.10 [95% CI −0.02 to 0.22]). About 70% of participants were rated as “downward movement”, and 9–11% as upward movement. Fair-to-moderate kappa values were found for evaluation of involuntary contraction by palpation. Over 80% of participants were rated as “absence of correct contraction”.

Ratings of voluntary relaxation showed large variations between different primary care rehabilitation centers, with weighted kappa values ranging from −0.08 to 0.56. The application of the scale significantly differed between primary care rehabilitation center 3 and primary care rehabilitation centers 1 + 2. At primary care rehabilitation center 3, the physiotherapists rated 25 of 29 assessments as showing complete voluntary relaxation. In contrast, at primary care rehabilitation centers 1 + 2, 10–12% of participants were rated as absent voluntary relaxation, 66% as partly relaxed, and 20–24% as complete voluntary relaxation. Primary care rehabilitation center 2 showed a negative kappa value of −0.08, indicating an agreement worse than expected or no agreement.[25]

Clinical assessment of the diastasis recti abdominis

Diastasis recti abdominis width, depth, and bulging were assessed in 159 women. The mean diastasis recti abdominis widths at three months postpartum was 25.9 mm at the umbilicus (Table 3). The diastasis recti abdominis widths measured at rehabilitation center 3 were significantly wider at all three measure points compared to rehabilitation centers 1 and 2 (p value<0.01). The DRA widths measured at the umbilicus was significantly wider in women with more than one child at rehabilitation center1 (p value= 0.04) but not at rehabilitation center 2+3.

For peer review only - http://bmjopen.bmj.com/site/about/guidelines.xhtml

Table 3. *Diastasis recti abdominis width at three months postpartum (in mm) measured with a caliper*

	Above the umbilicus<br>*Mean* ±SD<br>95% CI	At the umbilicus<br>*Mean* ±SD<br>95% CI	Below the umbilicus<br>*Mean* ±SD<br>95% CI
Total group (n = 159)	22.0 ±7.4<br>[20.9; 23.2]	25.9 ±7.2<br>[24.8; 27.1]	19.6 ±7.3<br>[18.4; 20.7]
Primipara (n=97)	20.73 ±6.0<br>[19.5;21.9]	24.3±5.7<br>[23.1; 25.4]	19.0 ±9.9<br>[17.6; 20.4]
Multipara (n= 62)	24.1±8.9<br>[21.9; 26.4]	28.7±8.5<br>[26.5; 30.8]	20.6±7.9<br>[18.6; 22.6]
Primary care rehabilitation center 1 (n = 61)	19.8 ±5.3<br>[18.5; 21.2]	24.1 ±6.5<br>[22.5; 25.8]	14.8 ±5.8<br>[13.3; 16.2]
Primipara (n=35)	18.7 ±4.5<br>[17.2; 20.3]	22.6 ±5.1<br>[20.9; 24.4]	13.7±5.1<br>[12.0; 15.5]
Multipara (n= 26)	21.3 ±6.0<br>[18.8; 23.9]	26.1 ±7.6<br>[23.1; 29.5]	16.2 ±6.4<br>[13.5; 18.8]
Primary care rehabilitation center 2 (n = 69)	20.6 ±4.6<br>[19.5; 21.7]	24.7 ±5.2<br>[23.5; 25.9]	21.1 ±4.6<br>[20.0; 22.2]
Primipara (n=51)	20.4 ±4.5<br>[19.1; 21.7]	24.3 ±4.6<br>[23.0; 25.6]	21.3 ±4.2<br>[20.1; 22.5]
Multipara (n= 18)	21.3 ±4.9<br>[18.9; 23.8]	25.9±6.6<br>[22.6; 29.1]	20.4 ±5.8<br>[17.6; 23.3]
Primary care rehabilitation center 3 (n = 29)	29.9 ±10.9<br>[25.8; 34.1]	32.8 ±9.0<br>[29.3; 36.2]	26.1 ±9.0<br>[22.7; 29.6]
Primipara (n=11)	28.6 ±9.4<br>[22.3; 34.9]	29.5 ±9.2<br>[23.3; 35.7]	25.0 ±11.0<br>[17.6; 32.4]
Multipara (n= 18)	30.7 ±11.9<br>[24.8; 36.6]	34.8 ±8.5<br>[30.5; 39.0]	26.9 ±7.8<br>[23.0; 30.7]

n, number; SD, standard deviation.

For peer review only

The measurement of diastasis recti abdominis width with a caliper showed good reliability when measured at the umbilicus and 4.5 cm below the umbilicus, and moderate reliability at 4.5 cm above the umbilicus (Table 4). For the total group, the SEM was between 4.05–4.75 mm, and the MDC was 11.23–13.17 mm. Sub-analysis of the different primary care rehabilitation centers revealed two negative outliers. At primary care rehabilitation center 2, assessment at 4.5 cm below the umbilicus showed an ICC value of 0.51 [95% CI: 0.20 to 0.70], which is at the lower boundary of the definition for moderate reliability. Assessment at 4.5 cm above the umbilicus at primary care rehabilitation center 3 showed much lower ICC values compared to the other values. An ICC value of 0.40 indicates low reliability.

Table 4. *Results of the clinical assessment of diastasis recti abdominis width with a caliper (in mm)*

Parameters	Total group (*n* = 159)	Primary care rehabilitation center 1 (*n* = 61)	Primary care rehabilitation center 2 (*n* = 69)	Primary care rehabilitation center 3 (*n* = 29)
	ICC [95% CI]<br>SEM; MDC	ICC [95% CI]<br>SEM; MDC	ICC [95% CI]<br>SEM; MDC	ICC [95% CI]<br>SEM; MDC
**Above the umbilicus**	0.73 [0.63; 0.80]<br>4.75; 13.17	0.78 [0.63; 0.87]<br>5.32; 14.75	0.60 [0.36; 0.75]<br>3.46; 9.59	0.40 [−0.32; 0.72]<br>8.30; 23.01
**At the umbilicus**	0.83 [0.76; 0.87]<br>4.05; 11.23	0.85 [0.75; 0.91]<br>3.29; 9.12	0.62 [0.39; 0.77]<br>4.34; 12.03	0.82 [0.61; 0.91]<br>4.93; 13.66
**Below the umbilicus**	0.80 [0.72; 0.85]<br>4.40; 12.20	0.75 [0.58; 0.85]<br>3.64; 10.09	0.51 [0.20; 0.70]<br>4.03; 11.17	0.74 [0.43; 0.88]<br>6.16; 17.07

n, number; ICC, intraclass correlation coefficient; CI, confidence interval; SEM, standard error of measurements; MDC, minimal detectable change.

The assessment of diastasis recti abdominis depth showed fair-to-moderate weighted kappa values ranging from 0.34–0.43 (Table 5). In the assessment of the diastasis recti abdominis depth, 21% were assessed as “good resistance at all points”, 67% as “resistance

For peer review only - http://bmjopen.bmj.com/site/about/guidelines.xhtml

in the depth", and 12% as "bottomless". Assessment of diastasis recti abdominis bulging in the 3-step sit-up test showed moderate agreement (kappa value, 0.51 [95% CI 0.29 to 0.73]). The diastasis recti abdominis bulging was rated in all participants, even those who were not able to perform a complete sit-up. According to Lo et al, the diastasis recti abdominis bulging occurs on exertion.[26] Our hypothesis was that all participants did their individual maximal exertion to perform a sit-up even if it was not resulting into a complete sit-up. However, the assessing physiotherapist had the rating option "cannot assess" in case of insecurity." The values primary care rehabilitation center 2 and 3 were lower than at primary care rehabilitation center and showed wide ranges in the 95% CI. Among the participants, about 81% were rated as "no bulging", 12% as "diastasis recti abdominis bulging", and 7% as "cannot assess".

Table 5. *Results of the clinical assessment of diastasis recti abdominis depth and bulging via observation and palpation*

	Total Group (n = 159)		Primary care rehabilitation center 1 (n = 61)		Primary care rehabilitation center 2 (n = 69)		Primary care rehabilitation center 3 (n = 29)	
	Kappa [95% CI]	PA %	Kappa [95% CI]	PA %	Kappa [95% CI]	PA %	Kappa [95% CI]	PA %
**Depth**	0.43 [0.29; 0.56]	69	0.37 [0.15; 0.59]	70	0.34 [0.11; 0.58]	71	0.36 [0.04; 0.69]	62
**Bulging**[c]	0.51 [0.29; 0.73]	88	0.77 [0.52; 1.02]	94	0.35 [0.04; 0.66]	83	0.36 [-0.16; 0.88]	88

n, number; CI, confidence interval; PA, percentage agreement.

c) The physiotherapists had the rating option of "cannot assess", and these assessments were excluded: Total group (n= 137); primary care rehabilitation center (n = 52); primary care rehabilitation center 2 (n= 59); and primary care rehabilitation center 3 (n = 26).

DISCUSSION

The main findings of this study are that physiotherapists managing women's health in primary care have reliable methods available to assess voluntary pelvic floor muscle contraction by vaginal palpation and measure diastasis recti abdominis width by caliper three months postpartum. On the other hand, the assessment of involuntary pelvic floor muscle contraction

For peer review only - http://bmjopen.bmj.com/site/about/guidelines.xhtml

by observation and voluntary pelvic floor muscle relaxation had kappa values with slight-to-fair agreement. Data with such low agreement are not useful for clinical practice or research.[25] Further investigations are needed to improve the clinical applicability and reliable assessment of these functions.

Clinical assessment of the pelvic floor muscles

Our present results showed weighted kappa values of 0.59–0.70 for the assessment of maximal voluntary contraction, which are higher compared to previous studies.[27-29] One explanation could be differences between the rating scales used for this muscle function. For example, in two prior studies,[27,28] only the squeezing and not the lifting component of the contraction was rated. Devreese *at al* discussed the importance of the lifting component of pelvic floor muscle strength, showing that incontinent women showed less inward movement than continent women.[30] Furthermore, the differences to our results could also be related to differences in the study population and smaller sample sizes in previous studies.

The assessment of pelvic floor muscle endurance showed moderate reliability. However, there were several inconsistencies in the pelvic floor muscle endurance data. Assessment of pelvic floor muscle endurance at primary care rehabilitation center 2 showed only fair reliability, with a kappa value of 0.27 [95% CI 0.09 to 0.45]. Our present results are lower compared with the findings of Devreese *et al*..[30] Notably, in the study of Devreese *et al*, a contraction longer than 10 seconds was rated as positive rather than a contraction longer than 30 seconds, as in the present study. Additionally, their study population was older and not three months postpartum. It may be more difficult to assess the exact point of time when the contraction is subsiding in a longer time period. It is also possible that postpartum women have, on average, a weaker contraction, making it more difficult to assess pelvic floor muscle endurance. Further research is needed to decide whether a pelvic floor muscle endurance of 10 seconds or 30 seconds is more clinically relevant.

For peer review only - http://bmjopen.bmj.com/site/about/guidelines.xhtml

Voluntary contraction via observation showed fair-to-moderate weighted kappa values. A prior MRI study reported that the average inward movement of the perineum is about 1 cm while sitting, and it is more than 2 cm in the supine position, according to Kegel in 1952.[31] It could be assumed that this large movement would be easy to observe, and a higher kappa value was expected. A previous study reported high inter-rater reliability in the observation of inward perineum movement.[14] Correspondingly, another study showed that inward perineal movement could be observed with a kappa value of 0.91 among continent women, and 0.93 among incontinent women- [30]

As factors other than pelvic floor muscle strength may contribute to urinary leakage postpartum,[32] it is important to assess other aspects of pelvic floor muscle function. Unfortunately, in our present study, we did not find a reliable method for assessing involuntary contraction by observation, and we demonstrated inconsistent findings for the assessment of involuntary contraction by palpation. Accordingly, another study reported only fair inter-rater reliability for the assessment of involuntary contraction by observation and palpation.[14] Further studies are needed to develop improved methods for the assessment and rating of these pelvic floor muscle functions in clinical practice.

When prescribing postpartum pelvic floor muscle training, it must be considered that some women have overactive, and possibly painful pelvic floor muscles. [33] It remains unclear whether women with hypertonic pelvic floor muscles should be advised to do pelvic floor muscle training. In clinical practice, physiotherapists recommend an individualized approach. Our present results showed that the rating of voluntary relaxation had slight-to-fair inter-rater reliability. Slieker-ten *et al.* [14] reported similar findings. Another study used a five-step rating scale for relaxation after contraction, and reported a correlation of 0.34 between two raters.[33] Even, Slieker-ten *et al.* recommend the addition of more rating steps to the scale, e.g., incomplete relaxation in their discussion. Regardless of whether women with hypertonic pelvic

For peer review only - http://bmjopen.bmj.com/site/about/guidelines.xhtml

floor muscles need more support in pelvic floor muscle training or the recommendation of no pelvic floor muscle training, there remains a need for better methods of assessing this condition.

Clinical assessment of the diastasis recti abdominis

Our results from the present study showed moderate-to-good reliability in measuring diastasis recti abdominis width using a caliper, after two months of training and calibrating of the method. The characteristics of the diastasis recti abdominis at three months postpartum measured with the caliper at rehabilitation centre 1+2 were comparable with the diastasis recti abdominis characteristics after pregnancy measured by ultrasound,[34] indicating these values are true values for this population. However, the two months of training and calibrating of the method via discussion and additional training were important components of this study. One bias identified in our additional training was that the accurate head lift of just 2–3 cm was an important factor. This observation is supported by the study of Mota *et al*. [35] which showed that the distance between the two parts of the rectus abdominis decreased during a sit-up movement.

There was some inconsistencies in the data, with two outliers at primary care rehabilitation centers 2 + 3. The SEM and MDC were higher at primary care rehabilitation center 3 compared to primary care rehabilitation centers 1 + 2 (Table 4). The MDC measured at 4.5 cm above the umbilicus was over 2 cm, raising doubt about whether these results have any clinical relevance. Taking into concern, that the measured DRA width at primary care rehabilitation centers 3 are significantly wider than at primary care rehabilitation centers 1+2, seen in both primi- and multiparous women, questions about if it is more difficult to measure a wider diastasis recti abdominis or if the measures are not accurate have to been raised. Comparing the results from rehabilitation center 3 with the normal values for primiparous women of Mota el al. [34], there is an indication for residual problems with test performance even after retraining. At primary care rehabilitation center 3, fewer than 30 participants were recruited during the study period of over one year. These results indicate that in addition to

For peer review only - http://bmjopen.bmj.com/site/about/guidelines.xhtml

training and experience, some continuity in measuring the diastasis recti abdominis width with a caliper is necessary for reliable assessment.

The SEM in our present study was about 4–5 mm and the MDC was about 11-13 mm, which is higher than in the comparable study of Benjamin *et al.*[19] However, just two participants showed an inter-recti distance greater than 22 mm in their study, in our study the mean diastasis recti abdominis width was 25.9 mm. Furthermore, Benjamin *et al* is not discussing how much experience and training the two investigating physiotherapists had in measuring diastasis recti abdominis width with a caliper.

We found fair-to-moderate weighted kappa values for the assessment of diastasis recti abdominis depth. To the best of our knowledge, there is no other study with which to compare these results. About 63–66% of the assessments were rated as “resistance in the depth”. The investigating physiotherapists hypothesized that “resistance in the depth” was felt and assessed as soon as the participants did not activate their deeper abdominal muscles during the head lift. Accordingly, Lee and Hodges[20] described the increased tension in the linea alba caused by activation of the deep abdominal muscles. If this is true, the assessment of diastasis recti abdominis depth is an assessment of the tension in the linea alba due to musculus transversus abdominis activation during a head lift. Another hypothesis could be that diastasis recti abdominis depth is caused by a reduced stability in the linea alba and a greater laxity in the tissue.[36]A next step could be to correlate the depth with the width- with the hypothesis that transversus abdominis activation increases the width[35] and decreases the depth. However, the results of our study showed just fair-to-moderate inter-rater reliability which makes it questionable if palpation is the right method for the clinical assessment of diastasis recti abdominis depth. Future studies must more precisely define the pre-activation of the deep abdominal muscles in the assessment of diastasis recti abdominis depth. Similar considerations have to be raised about abdominal muscle pre-activation and diastasis recti abdominis bulging

For peer review only - http://bmjopen.bmj.com/site/about/guidelines.xhtml

during a sit-up curl. Before implementation of this methods to clinical practice, further validation of the assessments of diastasis recti abdominis depth and bulging, for example a correlation between ultrasound/ shear-wave elastography and palpation, is needed

Strengths and limitations

One strength of this study was the large sample size and the quantity of different aspects of muscle function. A comparable study assessing different aspects of pelvic floor muscle function in women with and without pelvic floor disorders included only 41 participants.[14] A review about diastasis recti abdominis assessment methods included studies with 20–106 participants, and these studies only examined diastasis recti abdominis width.[15] Another strength of this study was that we were able to perform the same tests at three different centers in different parts of west Sweden. This makes our results transferable for different physiotherapists using the same methods.

The present study also had several limitations. One was that we lacked access to the participants' delivery records. Thus, the descriptive statistics and the calculate statistically significant differences between the three rehabilitation centers regarding age, BMI, mode of delivery, pelvic floor tearing, and highest birth weight were based on self-reported data from the participants. Another issue was the uneven cell distribution seen in over 50% of the rating scales tested in this study. In the literature, it is controversial whether a low kappa value can be explained by uneven cell distribution or low prevalence of a condition. [37]

Another limitation but also a strength of this study was the initial assumption that four hours of training in measuring the diastasis recti abdominis width with a caliper would be enough training for the six assessing physiotherapists. Due to this misjudgment of complexity using this method, a total of 63 measurements had to be excluded. On the other hand, many different obstacles, and sources of error for the measurements could be found through this initial phase and the added training. Due to the troubleshooting around the right application and

For peer review only - http://bmjopen.bmj.com/site/about/guidelines.xhtml

procedure while measuring the diastasis recti abdominis with a caliper, the procedure of this study might not 100% comparable with other studies. There are just a few existing studies measuring by caliper[18,38-41], several measurement positions like at rest, in a crunch position and in a modified curl-up were used, no consent about the right application method is found yet. In the training sessions to this study, we found it almost impossible to measure in resting, even if other studies[40,41] described the measurement in resting as reliable methods. At the same time, we registered that the higher the head lift/shoulder lift the less width was measurable compared to at rest which is in line with a study showing that an abdominal crunch is narrowing the inter-recti distance.[35] Our intention was to find an active position without missing the actual width of the diastasis recti abdominis. It would have been even better to validate these caliper measurements against ultrasound measurements. However, the aim of this study was to evaluate clinical applicable methods, which are easy to use and inexpensive tools for daily practice. Validation via ultrasound could be a next step.

Future research

Regarding the pelvic floor muscles, there remains a need for further research on how to assess and rate involuntary contraction, and voluntary relaxation, in the clinical assessment of women after pregnancy due to the high clinical relevance of this functions.

For the diastasis recti abdominis assessment, the depth and bulging have to be further defined and more knowledge about the behavior of the linea alba during muscle contraction and exertion is needed. For both pelvic floor muscle and diastasis recti abdominis assessment, we need to investigate the extent to which these values are clinically relevant for postpartum women—for example, if the assessment outcomes are associated with pain and dysfunction. Furthermore, we must determine a cut-off point for diastasis recti abdominis severity relative to pain and dysfunction. It will also be important to determine what training advice should be given to postpartum women based on the results of these examinations.

For peer review only - http://bmjopen.bmj.com/site/about/guidelines.xhtml

CONCLUSION

Women are increasingly requesting assessment of their pelvic floor muscles and diastasis recti abdominis after pregnancy. Our present results revealed moderate-to-substantial reliability for the assessment of maximal voluntary contraction and pelvic floor muscle endurance by vaginal palpation, considering both the lifting and squeezing component of the contraction. Furthermore, diastasis recti abdominis width can be measured by caliper, with a standard error of measurements of 4–5 mm and a minimal detectable change of about 1.2 cm. However, assessment using this instrument requires some training and experience. More research about the assessment of the involuntary contraction and voluntary relaxation of the pelvic floor muscles, as well as the diastasis recti abdominis depth and bulging is needed, before clinical implementation.

Acknowledgements

We thank Lisa Altvall, Ulrika Hansson, Anna-Lena Magnberg, Vanesa Rufete Bernal, Ute Jesberg, and Johanna Ekberg, who assessed the 222 women in the current study.

Competing Interests

The authors report no conflict of interest.

Funding statement

This study was funded by the Local Research and Development Council Gothenburg and Södra Bohuslän (Grant Number VGFOUGSB-762581) and The Healthcare Board, Region Västra Götaland (Grant Number VGFOUREG-751101).

Availability of data and materials

The dataset from this study is available via a reasonable request to the corresponding author.

For peer review only - http://bmjopen.bmj.com/site/about/guidelines.xhtml

Author statement

Each author of this paper meets the criteria for authorship. SV, MFO, AG, GR, and MEHL designed the study. SV collected the data, analyzed, and performed a first interpretation of the results, and drafted the article. MFO, AG, GR, and MEHL participated in the final interpretation of the results, and critically revised the article for important intellectual content. The final manuscript was seen and approved by all authors.

Consent for publication

The individuals shown in Image a+b gave their written informed consent to have these images published in a scientific publication.

List of Abbreviations

CI: Confidence interval; BMI: Body mass index; ICC: Intraclass correlation coefficient; MDC: Minimal detectable change; PA: Percentage agreement; SD: Standard deviation; SEM: Standard error of measurements

Ethical Approval Statement

This study was approved by the Swedish ethical review authority in Gothenburg in April 2018 (Dnr 088-18).

REFERENCES

1. Hodges PW, Sapsford R, Pengel LHM. Postural and respiratory functions of the pelvic floor muscles. *Neurourol. Urodyn.* 2007;26(3):362-371.
2. Smith MD, Coppieters MW, Hodges PW. Postural response of the pelvic floor and abdominal muscles in women with and without incontinence. *Neurourol. Urodyn.* 2007;26(3):377-385.

For peer review only - http://bmjopen.bmj.com/site/about/guidelines.xhtml

3. Lien K-C, Mooney B, DeLancey JOL, Ashton-Miller JA. Levator ani muscle stretch induced by simulated vaginal birth. *Obstet. Gynecol.* 2004;103(1):31-40.
4. Staer-Jensen J, Siafarikas F, Hilde G, Benth JS, Bo K, Engh ME. Postpartum recovery of levator hiatus and bladder neck mobility in relation to pregnancy. *Obstet. Gynecol.* 2015;125(3):531-539.
5. South MMT, Stinnett SS, Sanders DB, Weidner AC. Levator ani denervation and reinnervation 6 months after childbirth. *Am. J. Obstet. Gynecol.* 2009;200(5):519.e511-519.e517.
6. Cyr M-P, Kruger J, Wong V, Dumoulin C, Girard I, Morin M. Pelvic floor morphometry and function in women with and without puborectalis avulsion in the early postpartum period. *Am. J. Obstet. Gynecol.* 2017;216(3):274.e271-274.e278.
7. Memon H, Handa VL. Pelvic floor disorders following vaginal or cesarean delivery. *Curr. Opin. Obstet. Gynecol.* 2012;24(5):349-354.
8. Sperstad JB, Tennfjord MK, Hilde G, Ellström-Engh M, Bø K. Diastasis recti abdominis during pregnancy and 12 months after childbirth: prevalence, risk factors and report of lumbopelvic pain. *British Journal of Sports Medicine.* 2016.
9. Keshwani N, Mathur S, McLean L. Relationship Between Interrectus Distance and Symptom Severity in Women With Diastasis Recti Abdominis in the Early Postpartum Period. *Phys. Ther.* 2018;98(3):182-190.
10. Vieillefosse S, Conté C, Deffieux X. Symptom associated with diastasis recti in the early postpartum period. *European Journal of Obstetrics & Gynecology and Reproductive Biology.* 2019;234:e133-e133.
11. Keeler J, Albrecht M, Eberhardt L, Horn L, Donnelly C, Lowe D. Diastasis Recti Abdominis: A Survey of Women's Health Specialists for Current Physical Therapy Clinical Practice for Postpartum Women. *Journal of Women's Health Physical Therapy.* 2012;36(3):131-142.

For peer review only

For peer review only - http://bmjopen.bmj.com/site/about/guidelines.xhtml

12. Bo K, Sherburn M. Evaluation of female pelvic-floor muscle function and strength. *Phys. Ther.* 2005;85(3):269-282.
13. Messelink B, Benson T, Berghmans B, et al. Standardization of terminology of pelvic floor muscle function and dysfunction: report from the pelvic floor clinical assessment group of the International Continence Society. *Neurourol. Urodyn.* 2005;24(4):374-380.
14. Slieker-Ten Hove MCP, Pool-Goudzwaard AL, Eijkemans MJC, Steegers-Theunissen RPM, Burger CW, Vierhout ME. Face validity and reliability of the first digital assessment scheme of pelvic floor muscle function conform the new standardized terminology of the International Continence Society. *Neurourol. Urodyn.* 2009;28(4):295-300.
15. van de Water ATM, Benjamin DR. Measurement methods to assess diastasis of the rectus abdominis muscle (DRAM): A systematic review of their measurement properties and meta-analytic reliability generalisation. *Man. Ther.* 2016;21:41-53.
16. Keeler J, Albrecht M, Eberhardt L, Horn L, Donnelly C, Lowe D. Diastasis Recti Abdominis: A Survey of Women's Health Specialists for Current Physical Therapy Clinical Practice for Postpartum Women. *Journal of Women's Health Physical Therapy.* 2012;36(3):131-142.
17. Bursch SG. Interrater reliability of diastasis recti abdominis measurement. *Phys Ther.* 1987;67(7):1077-1079.
18. Barbosa S, de Sa RA, Coca Velarde LG. Diastasis of rectus abdominis in the immediate puerperium: correlation between imaging diagnosis and clinical examination. *Archives of gynecology and obstetrics.* 2013;288(2):299-303.
19. Benjamin DR, Frawley HC, Shields N, Georgiou C, Taylor NF. Establishing measurement properties in the assessment of inter-recti distance of the abdominal muscles in a postnatal women. *Musculoskeletal science & practice.* 2020;49:102202.

For peer review only - http://bmjopen.bmj.com/site/about/guidelines.xhtml

20. Lee D, Hodges PW. Behavior of the Linea Alba During a Curl-up Task in Diastasis Rectus Abdominis: An Observational Study. *The Journal of orthopaedic and sports physical therapy.* 2016;46(7):580.

21. Beamish N, Green N, Nieuwold E, McLean L. Differences in Linea Alba Stiffness and Linea Alba Distortion Between Women With and Without Diastasis Recti Abdominis: The Impact of Measurement Site and Task. *The Journal of orthopaedic and sports physical therapy.* 2019;49(9):656-665.

22. Lee DG, Lee LJ, McLaughlin L. Stability, continence and breathing: The role of fascia following pregnancy and delivery. *J. Bodyw. Mov. Ther.* 2008;12(4):333-348.

23. Koo TK, Li MY. A Guideline of Selecting and Reporting Intraclass Correlation Coefficients for Reliability Research. *J. Chiropr. Med.* 2016;15(2):155-163.

24. Landis JR, Koch GG. A One-Way Components of Variance Model for Categorical Data. *Biometrics.* 1977;33(4):671-679.

25. McHugh ML. Interrater reliability: the kappa statistic. *Biochemia medica.* 2012;22(3):276-282.

26. Lo T, Candido G, P J. Diastasis of the Recti abdominis in pregnancy: risk factors and treatment. *Physiother. Can.* 1999;51(1):32-44.

27. Bø K, Finckenhagen HB. Vaginal palpation of pelvic floor muscle strength: inter-test reproducibility and comparison between palpation and vaginal squeeze pressure. *Acta Obstet. Gynecol. Scand.* 2001;80(10):883-887.

28. Ferreira CH, Barbosa PB, de Oliveira Souza F, Antonio FI, Franco MM, Bo K. Inter-rater reliability study of the modified Oxford Grading Scale and the Peritron manometer. *Physiotherapy.* 2011;97(2):132-138.

29. Navarro Brazalez B, Torres Lacomba M, de la Villa P, et al. The evaluation of pelvic floor muscle strength in women with pelvic floor

For peer review only

dysfunction: A reliability and correlation study. *Neurourol. Urodyn.* 2018;37(1):269-277.

30. Devreese A, Staes F, De Weerdt W, et al. Clinical evaluation of pelvic floor muscle function in continent and incontinent women. *Neurourol. Urodyn.* 2004;23(3):190-197.
31. Bo K, Lilleas F, Talseth T, Hedland H. Dynamic MRI of the pelvic floor muscles in an upright sitting position. *Neurourol. Urodyn.* 2001;20(2):167-174.
32. Fritel X, Ringa V, Quiboeuf E, Fauconnier A. Female urinary incontinence, from pregnancy to menopause: a review of epidemiological and pathophysiological findings. *Acta Obstet. Gynecol. Scand.* 2012;91(8):901-910.
33. Reissing ED, Brown C, Lord MJ, Binik YM, Khalifé S. Pelvic floor muscle functioning in women with vulvar vestibulitis syndrome. *Journal of Psychosomatic Obstetrics & Gynecology.* 2005;26(2):107-113.
34. Mota P, Pascoal AG, Carita AI, Bo K. Normal width of the inter-recti distance in pregnant and postpartum primiparous women. *Musculoskeletal science & practice.* 2018;35:34-37.
35. Mota P, Pascoal AG, Carita AI, Bo K. The Immediate Effects on Inter-rectus Distance of Abdominal Crunch and Drawing-in Exercises During Pregnancy and the Postpartum Period. *J. Orthop. Sports Phys. Ther.* 2015;45(10):781-788.
36. Fiori F, Ferrara F, Gobatti D, Gentile D, Stella M. Surgical treatment of diastasis recti: the importance of an overall view of the problem. *Hernia.* 2020.
37. Vach W. The dependence of Cohen's kappa on the prevalence does not matter. *J. Clin. Epidemiol.* 2005;58(7):655-661.
38. Dalal K, Kaur A, Mitra M. Correlation between Diastasis Rectus Abdominis and Lumbopelvic Pain and Dysfunction. *Indian Journal of Physiotherapy and Occupational Therapy.* 2014;8(1):210-214.

For peer review only - http://bmjopen.bmj.com/site/about/guidelines.xhtml

39. Parker MA, Millar LA, Dugan SA. Diastasis Rectus Abdominis and Lumbo-Pelvic Pain and Dysfunction-Are They Related? *Journal of Women's Health Physical Therapy.* 2009;33(2):15-22.
40. Chiarello CM, McAuley JA. Concurrent Validity of Calipers and Ultrasound Imaging to Measure Interrecti Distance. *J. Orthop. Sports Phys. Ther.* 2013;43(7):495-503.
41. Boxer S, Jones S. Intra-rater reliability of rectus abdominis diastasis measurement using dial calipers. *Aust. J. Physiother.* 1997;43(2):109-114.

For peer review only

Supplement 1: Clinical assessment of pelvic floor and abdominal muscles three months postpartum: An inter-rater reliability study

Clinical assessment protocol: Diastasis recti abdominis

Due to the lack of information about how to apply the jaws of the caliper while measuring the diastasis recti abdominis, we conducted a pilot trial with ten participants before starting the study. The inside and the outside jaws of the caliper were used in random order.

The outside jaws of the caliper are designed to measure the outer boundaries of a firm subject. In the setting of this study, this would be the outer boundaries of the linea alba. The inside jaws of the caliper are designed to measure the inner boundaries of a firm subject, which in the present study would be the inner edges of the rectus abdominis. We found a mean difference of −2.32 mm (SD 2.47) between the inside and the outside jaws. Moreover, measuring with the inside jaws caused more discomfort for the participant, and more insecurity for the investigator regarding the subjective estimation of how much to draw in the inner edge of the rectus abdominis. Thus, it was decided to palpate the outer edges of the linea alba with the fingers, and to measure this distance with the outside jaws (Figure 1).

Figure 1. *Application of the caliper for diastasis recti abdominis measurement*

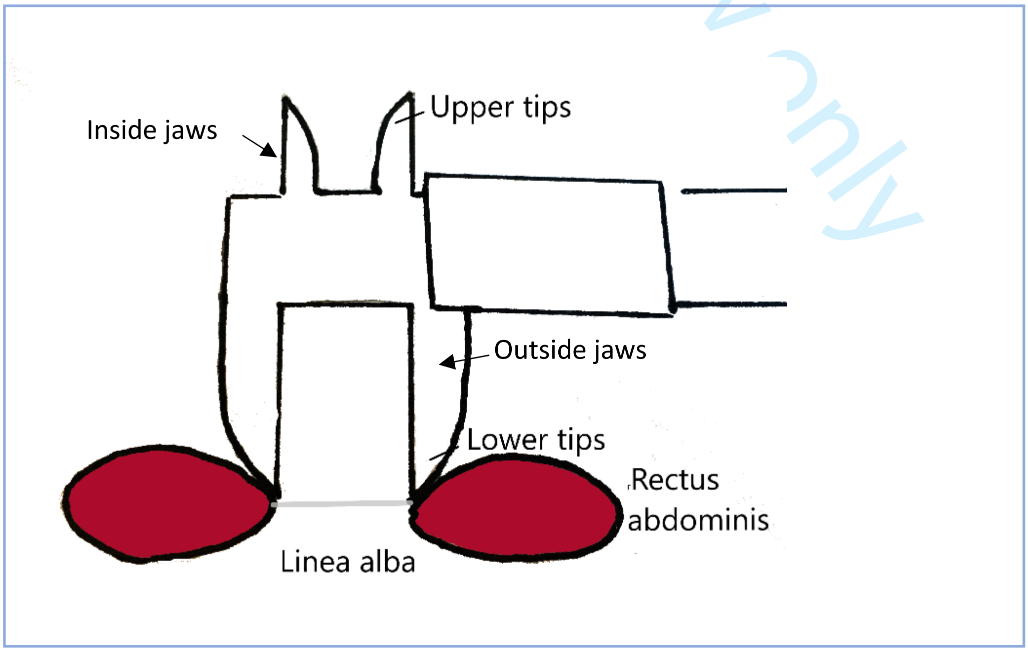

For peer review only

Supplement 1: Clinical assessment of pelvic floor and abdominal muscles three months postpartum: An inter-rater reliability study

Two months after start of the study in September 2018, the assessing physiotherapists expressed a strong uncertainty regarding the right technique for using the caliper. A pre-analysis of the data showed low-to-negative ICC values and large differences between the measurements. Thus, we conducted an additional training for all centers. The six physiotherapists underwent a calibration process in form of after each other assessing ten person`s abdominal muscles, comparing their results and discussing their technique. At this point in time, rehabilitation center 3 had not yet started their assessments. In the additional training, the assessing physiotherapists identified three important confounders while measuring:

1. Accurate lift of the head (2–3 cm) was an important factor for correct measurement. With less lift, there was no contraction in the abdominal muscles and it was almost impossible to find the inner edges of the *musculus rectus abdominis* and with a higher lift the distance between the two bellies of the *musculus recti abdominis* was decreasing which is in line with the study of Mota et al showing that an abdominal crunch is narrowing the inter-recti distance.[1]
2. Pre-activation in the deeper abdominal muscles impeded the measurement. Due to this the participants were encouraged to relax before lifting the head.
3. It was easier to palpate the junction between the rectus abdominis and linea alba during the eccentric movement of lowering the head.

<u>After the additional training, the assessment protocol was set as follows:</u>

The diastasis recti abdominis was assessed with the participant in the supine position on a flat bench, with the legs flexed, and their arms held at their side. The assessing physiotherapist used a water-soluble marker to mark the three measurement points: at the umbilicus, and at 4.5 cm above and 4.5 cm below the umbilicus.[2,3] For accurate assessment of the activated

For peer review only - http://bmjopen.bmj.com/site/about/guidelines.xhtml

Supplement 1: Clinical assessment of pelvic floor and abdominal muscles three months postpartum: An inter-rater reliability study

abdominal muscles, the participant had to lift her head 2–3 cm from the bench. Before the assessment began, the physiotherapist assured that the participant correctly lifted her head 2–3 cm, which was trained by several repetitions.

Diastasis recti abdominis width: The diastasis recti abdominis width was defined as the measured distance between the two parts of the rectus abdominis.

- Clinical assessment: The participant was asked to lift her head and then slowly lower it. As seen in other studies[4,5], this concentric and eccentric movement is helping the physiotherapist to palpate the outer edges of the linea alba with their index and middle finger, without examination gloves. To avoid pre-activation of the deeper abdominal muscle, the participants were asked to relax before performing the trained head lift of 2–3 cm. During this movement -resulting in an activation of the musculus rectus abdominis- the physiotherapist identified the distance between the two parts of the rectus abdominis with her fingers and measured this felt distance using the caliper (Image 1a). The same procedure was conducted at all three measurement points.

Image 1. a) *Measuring the diastasis recti abdominis width with the caliper.* ***b)*** *Observation of diastasis recti abdominis bulging*

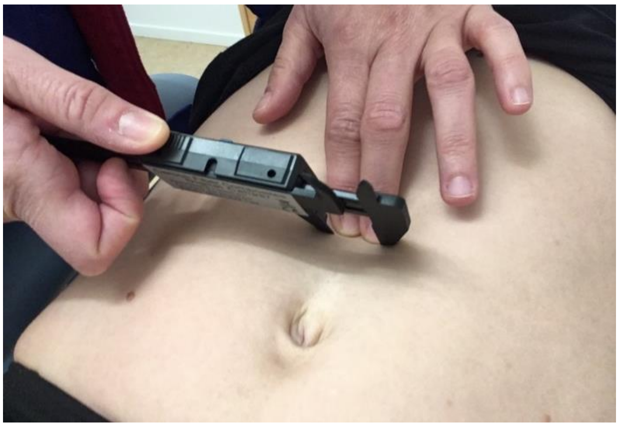

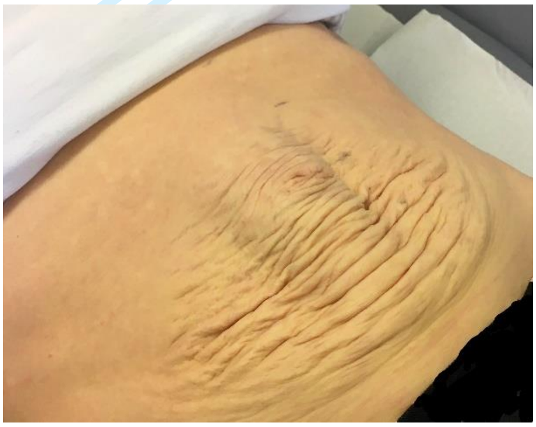

For peer review only - http://bmjopen.bmj.com/site/about/guidelines.xhtml

Supplement 1: Clinical assessment of pelvic floor and abdominal muscles three months postpartum: An inter-rater reliability study

Diastasis recti abdominis depth: The diastasis recti abdominis depth was defined as the palpated tension in the linea alba during a head lift.

- Clinical assessment: The participant was asked to repeat the exact same head lift of 2–3 cm. At all three measurement points, the physiotherapist palpated the tension in the linea alba (without adding pressure), and rated it as "good resistance at all points", "resistance in the depth at measurement point x", or "bottomless resistance at measurement point x".

Diastasis recti abdominis bulging: Diastasis recti abdominis bulging was defined as midline bulge on exertion[6], which is also seen in Image 1b.

- Clinical assessment: The participant performed a 3-step sit-up test.[7] The participant was laying supine with straight legs on the flat bench. The test was rated as 0 if the participant was not able to perform a sit-up, as 1 if the participant was able to raise the upper torso to 40-degree angle from the bench with straight and secured legs, arms at the side of the body. The test was rated as 2 if the participant was able to perform the same task with the hands held behind the head and as 3 if the participant was able to perform a sit-up with the hands behind her head and hips and knees flexed and not secured. The participant had to hold the sit-up position for 5 seconds. During this test, the physiotherapist observed whether the linea alba bulged during the attempt to do a sit-up. Diastasis recti abdominis bulging was defined as midline bulge on exertion[6], which is also seen in Image 1b.

References:

1. Mota P, Pascoal AG, Carita AI, Bo K. The Immediate Effects on Inter-rectus Distance of Abdominal Crunch and Drawing-in Exercises During Pregnancy and the Postpartum Period. *J. Orthop. Sports Phys. Ther.* 2015;45(10):781-788.

For peer review only - http://bmjopen.bmj.com/site/about/guidelines.xhtml

Supplement 1: Clinical assessment of pelvic floor and abdominal muscles three months postpartum: An inter-rater reliability study

2. Chiarello CM, Falzone LA, McCaslin KE, Patel MN, Ulery KR. The effects of an exercise program on diastasis recti abdominis in pregnant women. *Journal of Women's Health Physical Therapy.* 2005;29(1):11-16.
3. Chiarello CM, McAuley JA. Concurrent validity of calipers and ultrasound imaging to measure interrecti distance. *The Journal of orthopaedic and sports physical therapy*. 2013;43(7):495-503.
4. Parker MA, Millar LA, Dugan SA. Diastasis Rectus Abdominis and Lumbo-Pelvic Pain and Dysfunction-Are They Related? *Journal of Women's Health Physical Therapy*. 2009;33(2):15-22.
5. Dalal K, Kaur A, Mitra M. Correlation between Diastasis Rectus Abdominis and Lumbopelvic Pain and Dysfunction. *Indian Journal of Physiotherapy and Occupational Therapy*. 2014;8(1):210-214.
6. Lo T, Candido G, P J. Diastasis of the Recti abdominis in pregnancy: risk factors and treatment. *Physiother. Can.* 1999;51(1):32-44.
7. Hills NF, Graham RB, McLean L. Comparison of Trunk Muscle Function Between Women With and Without Diastasis Recti Abdominis at 1 Year Postpartum. *Phys. Ther*. 2018;98(10):891.

For peer review only

Supplement 2: Clinical assessment of pelvic floor and abdominal muscles three months postpartum: An inter-rater reliability study

Clinical assessment protocol: Pelvic floor muscles

The pelvic floor muscles were assessed with the participant in the supine position, with the legs flexed and slightly abducted on a plinth, and a pillow under the head. Participants were assessed by observation and digital palpation. During observation of pelvic floor muscle contraction, the physiotherapist stood beside the bench, supporting the participant's legs with her hands and observing the movement of the perineum:

- **Involuntary contraction**: The participant was asked to cough forcefully; the movement of the perineum were rated as downwards/ perineal in-drawing/ no movement
- **Voluntary contraction**, the participant was given the verbal cue "contract your pelvic floor muscles like you want to prevent the escape of gas/urine". The movement of the perineum was rated as downwards/ perineal in-drawing/ no movement

Vaginal palpation of the pelvic floor muscles was performed by physiotherapists using examinations gloves and water-based lubricant. The index and middle finger were inserted 2–3 cm into the vagina with the palmar side directed to the caudal part of the vagina, palpating the pubovisceral portion of the levator ani.[1]

- **Involuntary contraction**: The participant was asked to forcefully cough three times. The absence or presence of a correct contraction, defined as a squeeze around the pelvic openings and an inward lift,[2] were rated.
- **Maximal voluntary contraction,** the participant was asked to contract the pelvic floor muscles. If the physiotherapist felt a correct contraction, the participant was encouraged to activate their pelvic floor muscles "as strong and as long you can". Of three maximal voluntary contractions, the strongest was rated on a 6-point

For peer review only

modified Oxford Scale. The scale was modified for the study with the aim to assess both the lifting and the squeezing component of the pelvic floor muscle function. The onset of the lifting component in the contraction were set as a criterion for being rated ≥3 on the scale (Figure 2).

***Figure 1**. The modified Oxford Scale used in this study*

0	No contraction
1	Partial contraction, “flicker”
2	Weak contraction, no correct lift
3	Good contraction, definite lift
4	Maximum contraction with lift, not able to hold longer than 5 seconds
5	Maximum contraction with lift, able to hold ___ seconds

The participants rested 15 seconds between the contractions. If a participant, despite several attempts and verbal cues, failed to squeeze and lift and was instead straining, the participant´s results were excluded from the statistical analysis of maximal voluntary contraction and pelvic floor muscle endurance.

- **Pelvic floor muscle endurance**, after 15 seconds of rest, the participant was asked to contract the pelvic floor muscles for as long as possible at approximately 50% of the previous contraction strength. Pelvic floor muscle endurance was rated as positive if the participant was able to hold this contraction for longer than 30 seconds.

For peer review only

Supplement 2: Clinical assessment of pelvic floor and abdominal muscles three months postpartum: An inter-rater reliability study

- **Voluntary relaxation**, the participant was given the verbal cue "try to relax your pelvic floor muscles, let the vagina get larger and go downwards". This function was rated as absent, partial, or complete.

References:

1. Kearney R, Miller JM, DeLancey JOL. Interrater reliability and physical examination of the pubovisceral portion of the levator ani muscle, validity comparisons using MR imaging. Neurourol. Urodyn. 2006;25(1):50-54.
2. Bo K, Sherburn M. Evaluation of female pelvic-floor muscle function and strength. Phys. Ther. 2005;85(3):269-282.

For peer review only

Section & Topic	No	Item	Reported on page #
**TITLE OR ABSTRACT**			
	**1**	Identification as a study of diagnostic accuracy using at least one measure of accuracy (such as sensitivity, specificity, predictive values, or AUC)	1
**ABSTRACT**			2
	**2**	Structured summary of study design, methods, results, and conclusions (for specific guidance, see STARD for Abstracts)	
**INTRODUCTION**			
	**3**	Scientific and clinical background, including the intended use and clinical role of the index test	3-5
	**4**	Study objectives and hypotheses	5
**METHODS**			
Study design	**5**	Whether data collection was planned before the index test and reference standard were performed (prospective study) or after (retrospective study)	5
Participants	**6**	Eligibility criteria	6
	**7**	On what basis potentially eligible participants were identified (such as symptoms, results from previous tests, inclusion in registry)	6
	**8**	Where and when potentially eligible participants were identified (setting, location and dates)	6
	**9**	Whether participants formed a consecutive, random or convenience series	6
Test methods	**10a**	Index test, in sufficient detail to allow replication	7, supplement 1+2
	**10b**	Reference standard, in sufficient detail to allow replication	N/A
	**11**	Rationale for choosing the reference standard (if alternatives exist)	N/A
	**12a**	Definition of and rationale for test positivity cut-offs or result categories of the index test, distinguishing pre-specified from exploratory	N/A
	**12b**	Definition of and rationale for test positivity cut-offs or result categories of the reference standard, distinguishing pre-specified from exploratory	N/A
	**13a**	Whether clinical information and reference standard results were available to the performers/readers of the index test	17
	**13b**	Whether clinical information and index test results were available to the assessors of the reference standard	N/A
Analysis	**14**	Methods for estimating or comparing measures of diagnostic accuracy	8-9
	**15**	How indeterminate index test or reference standard results were handled	8-9
	**16**	How missing data on the index test and reference standard were handled	N/A
	**17**	Any analyses of variability in diagnostic accuracy, distinguishing pre-specified from exploratory	8-9
	**18**	Intended sample size and how it was determined	5
**RESULTS**			
Participants	**19**	Flow of participants, using a diagram	N/A
	**20**	Baseline demographic and clinical characteristics of participants	10
	**21a**	Distribution of severity of disease in those with the target condition	N/A
	**21b**	Distribution of alternative diagnoses in those without the target condition	10
	**22**	Time interval and any clinical interventions between index test and reference standard	N/A
Test results	**23**	Cross tabulation of the index test results (or their distribution) by the results of the reference standard	N/A
	**24**	Estimates of diagnostic accuracy and their precision (such as 95% confidence intervals)	11, 14-15
	**25**	Any adverse events from performing the index test or the reference standard	10
**DISCUSSION**			
	**26**	Study limitations, including sources of potential bias, statistical uncertainty, and generalisability	16, 18-19
	**27**	Implications for practice, including the intended use and clinical role of the index test	20
**OTHER INFORMATION**			
	**28**	Registration number and name of registry	5-6
	**29**	Where the full study protocol can be accessed	21
	**30**	Sources of funding and other support; role of funders	21

For peer review only - http://bmjopen.bmj.com/site/about/guidelines.xhtml

cc BY SA

STARD 2015

AIM

STARD stands for “Standards for Reporting Diagnostic accuracy studies”. This list of items was developed to contribute to the completeness and transparency of reporting of diagnostic accuracy studies. Authors can use the list to write informative study reports. Editors and peer-reviewers can use it to evaluate whether the information has been included in manuscripts submitted for publication.

EXPLANATION

A **diagnostic accuracy study** evaluates the ability of one or more medical tests to correctly classify study participants as having a **target condition.** This can be a disease, a disease stage, response or benefit from therapy, or an event or condition in the future. A medical test can be an imaging procedure, a laboratory test, elements from history and physical examination, a combination of these, or any other method for collecting information about the current health status of a patient.

The test whose accuracy is evaluated is called **index test.** A study can evaluate the accuracy of one or more index tests. Evaluating the ability of a medical test to correctly classify patients is typically done by comparing the distribution of the index test results with those of the **reference standard**. The reference standard is the best available method for establishing the presence or absence of the target condition. An accuracy study can rely on one or more reference standards.

If test results are categorized as either positive or negative, the cross tabulation of the index test results against those of the reference standard can be used to estimate the **sensitivity** of the index test (the proportion of participants *with* the target condition who have a positive index test), and its **specificity** (the proportion *without* the target condition who have a negative index test). From this cross tabulation (sometimes referred to as the contingency or “2x2” table), several other accuracy statistics can be estimated, such as the positive and negative **predictive values** of the test. Confidence intervals around estimates of accuracy can then be calculated to quantify the statistical **precision** of the measurements.

If the index test results can take more than two values, categorization of test results as positive or negative requires a **test positivity cut-off**. When multiple such cut-offs can be defined, authors can report a receiver operating characteristic (ROC) curve which graphically represents the combination of sensitivity and specificity for each possible test positivity cut-off. The **area under the ROC curve** informs in a single numerical value about the overall diagnostic accuracy of the index test.

The **intended use** of a medical test can be diagnosis, screening, staging, monitoring, surveillance, prediction or prognosis. The **clinical role** of a test explains its position relative to existing tests in the clinical pathway. A replacement test, for example, replaces an existing test. A triage test is used before an existing test; an add-on test is used after an existing test.

Besides diagnostic accuracy, several other outcomes and statistics may be relevant in the evaluation of medical tests. Medical tests can also be used to classify patients for purposes other than diagnosis, such as staging or prognosis. The STARD list was not explicitly developed for these other outcomes, statistics, and study types, although most STARD items would still apply.

DEVELOPMENT

This STARD list was released in 2015. The 30 items were identified by an international expert group of methodologists, researchers, and editors. The guiding principle in the development of STARD was to select items that, when reported, would help readers to judge the potential for bias in the study, to appraise the applicability of the study findings and the validity of conclusions and recommendations. The list represents an update of the first version, which was published in 2003.

More information can be found on http://www.equator-network.org/reporting-guidelines/stard.

For peer review only - http://bmjopen.bmj.com/site/about/guidelines.xhtml
